# Two natural products from the seeds of *Citrus reticulata* Blanco (Rutaceae) inhibit estrogen biosynthesis by regulating the PI3K-aromatase pathway

**DOI:** 10.3389/fphar.2025.1583409

**Published:** 2025-06-12

**Authors:** Xing Yang, Xi Chen, Chunqiao Shi, Qian Zhang, Qian Liu, Chunyan Zhou, Fawu Dong, Jinsong Su, Deming Liu, Yi Zhang

**Affiliations:** ^1^ Chengdu University of Traditional Chinese Medicine, Chengdu, Sichuan, China; ^2^ Chongqing Clinical Research Center for Dermatology, Chongqing Key Laboratory of Integrative Dermatology Research, Key Laboratory of External Therapies of Traditional Chinese Medicine in Eczema, Department of Dermatology, Chongqing Traditional Chinese Medicine Hospital/the First Affiliated Hospital of Chongqing College of Traditional Chinese Medicine, Chongqing, China; ^3^ Department of General Surgery, Chongqing Traditional Chinese Medicine Hospital/the First Affiliated Hospital of Chongqing College of Traditional Chinese Medicine, Chongqing, China; ^4^ Yunnan Key Laboratory of Southern Medicinal Utilization, Yunnan University of Traditional Chinese Medicine, Kunming, China

**Keywords:** the seeds of *Citrus reticulata* Blanco (Rutaceae), aromatase, callyspongidipeptide A, Hesperetin 7-*O-β-D*-glucopyranoside, aromatase inhibitors

## Abstract

**Introduction:**

The seeds of Citrus reticulata Blanco (Rutaceae) (SCR), a traditional Chinese medicine derived from Citrus, is known for its diverse bioactivities, including potential anti-breast cancer effects, but the mechanism of action remains unclear.

**Methods:**

This study aims to elucidate the active ingredients of SCR and their mechanisms of action on estrogen biosynthesis. A comprehensive phytochemical analysis, employing various chromatographic techniques, led to the isolation of 26 compounds from SCR.

**Results:**

The effects of these compounds on estrogen biosynthesis were evaluated in human ovarian granulosa-like KGN cells, which play a crucial role in the progression of hormone-dependent breast cancers. Network pharmacology analysis revealed that SCR may influence breast cancer development by modulating phosphorylation-related biological processes and the PI3K/AKT pathway. Among the isolated compounds, Callyspongidipeptide A (Calp) and Hesperetin 7-O-β-D-glucopyranoside (Hesp) exhibited significant inhibitory effects on estrogen biosynthesis. Calp and Hesp selectively regulated the expression of aromatase (Aro) PI.3 and P2 promoters via the PI3K/AKT pathway, inhibiting Aro mRNA and protein expression.

**Discussion:**

These findings provide novel insights into the chemopreventive potential of SCR and support its role in the development of therapies aimed at reducing the risk of hormone-related cancers.

## Graphical Abstract

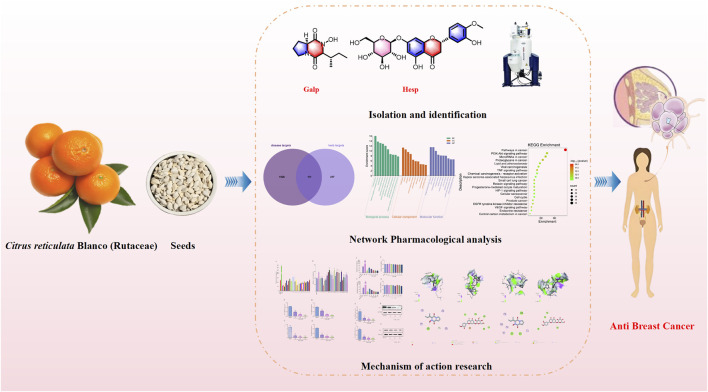

## 1 Introduction

Cancer represents an important global health concern, with breast cancer emerging as the most prevalent form of cancer among women, accounting for approximately 2.3 million new cases each year worldwide (Nolan et al., 2023). It has overtaken lung cancer as the most diagnosed cancer, highlighting its widespread effect (Sung et al., 2021; Roheel et al., 2023; Qian et al., 2023). Breast cancer is classified into three primary categories: estrogen receptor–positive (ER+) breast cancer, human epidermal growth factor receptor 2-positive (HER2+) breast cancer, and triple-negative breast cancer (TNBC) (Gradishar et al., 2024). Its etiology is complex, involving a myriad of factors including genetic predisposition, hormonal fluctuations, and lifestyle choices (Roberts et al., 2023). Although surgical resection remains the cornerstone of breast cancer treatment, a notable shift toward integrating drug-assisted systemic therapies has been seen due to advancements in medical technology and evolving treatment philosophies. Current clinical treatments incorporate chemotherapy, endocrine therapy, surgical resection, and targeted therapies. However, endocrine therapy is increasingly scrutinized for its tolerability and adverse side effects (Escala et al., 2020; Barzaman et al., 2020).

Numerous studies have shown that the development of breast cancer is closely linked to the levels of estrogens, such as estradiol, progesterone, and prolactin, with Estradiol (E2) playing a particularly important role (Contreras et al., 2019; Hu et al., 2019). Approximately 75% of breast cancer cases are estrogen-dependent, underscoring the critical involvement of estrogen, especially E2, in the disease’s pathogenesis (Mouridsen et al., 2003). Estrogens are steroid hormones that exert their effects predominantly through estrogen receptors, influencing various physiological processes through either transcriptional or nontranscriptional mechanisms (Özdemir et al., 2018; Bhatia and Thareja, 2024). The biosynthesis of E2 is critically regulated by Aromatase (Aro), an enzyme whose activity is essential for the conversion of androgens into estrogens (Rižner, 2013; Means et al., 1989; Ratre et al., 2020). The Aro gene, situated on chromosome 15q21.2, contains 10 exons, with the first exon unique in its role in tissue-specific expression (Chen et al., 2009; Lippman, 2018). Endocrine therapies, particularly Aro inhibitors, have been pivotal in the context of ER+ breast cancer. The combination of ovarian function suppression with either tamoxifen or Aro inhibitors has become a standard treatment for premenopausal ER+ patients, considerably reducing recurrence risk by 35%–48% in younger patients (Mahboobifard et al., 2022). Third-generation Aro inhibitors, such as letrozole, are now utilized as first-line treatments for postmenopausal women. As the third-generation Aro inhibitors directly target Aro to inhibit its catalytic activity and lacking tissue selectivity, they achieve good therapeutic effects but also cause adverse reactions in up to about 50% of patients, which are increasingly gaining attention. These side effects are mostly mild to moderate and can lead to symptoms such as hot flashes, insomnia, mood changes, hyperlipidemia, and osteoporosis. Approximately 25% of patients discontinue treatment due to intolerance. Therefore, it is of great significance to develop new aromatase inhibitors based on the transcriptional regulation and post-translational modifications of Aro.

Given this backdrop, exploring natural compounds with potential anti-Aro activity has become imperative. China, which is known as the world citrus resource bank, boasts an extensive array of citrus species cultivated across numerous provinces with a production capacity that leads worldwide (Mao et al., 2024). However, the disposal of the seeds of *Citrus reticulata* Blanco (Rutaceae) (SCR) as waste represents an environmental concern and an untapped resource (Xian et al., 2021). The traditional applications of SCR in Chinese medicine suggest its potential in treating various ailments, including cancer, attributed to its anti-inflammatory, analgesic, and antiviral properties (Chen et al., 2021). Although previous phytochemical analyses have identified limonoids, flavonoids, and volatile oils as key constituents of SCR, a comprehensive understanding of its active components against breast cancer remains elusive. Currently, research on the antibreast cancer effects of orange seeds mainly focuses on promoting apoptosis in breast cancer cells.

This study aims to elucidate further the chemical composition of SCR, focusing on identifying novel compounds that inhibit Aro activity, thereby offering potential lead compounds for the medicine.

## 2 Experimental

### 2.1 General experimental procedures

IR (KBr) spectra were obtained using a Thermo Scientific Nicolet iS10 spectrophotometer manufactured by Thermo Fisher Scientific in Massachusetts, United States UV spectra were measured on a Shimadzu UV-2401PC spectrophotometer, produced by Shimadzu in Kyoto, Japan. 1D and 2D NMR spectra were recorded on a Bruker AV 600, DRX 500, or AM-400 spectrometer, all made by Bruker in Bremerhaven, Germany, with TMS serving as the internal standard. Chemical shifts (*δ*) are expressed in ppm and referenced to the solvent signals. ESIMS analysis was conducted on a Shimadzu LC–MS-IT-TOF mass spectrometer, also from Kyoto, Japan, while HR-ESI-MS was recorded using an Agilent 6,200 series TOF instrument manufactured by Agilent Technologies in Palo Alto, United States. Semipreparative HPLC was performed using an Agilent 1260 Series system equipped with a C18 reversed-phase column (5 μm; 9.4 mm × 250 mm), supplied by Agilent Technologies in Palo Alto, United States. Column chromatography was carried out on silica gel of 200–300 mesh and 300–400 mesh, sourced from Qingdao Haiyang Chemical Co. Ltd. in Qingdao, People’s Republic of China. Additionally, Lichroprep RP-18 (40–63 μm) from Merck in Darmstadt, Germany, and Sephadex LH-20 gel (40–70 μm) from Amersham Pharmacia Biotech AB in Uppsala, Sweden, were utilized. Precoated silica gel GF254 plates were provided by Qingdao Haiyang Chemical Plant.

### 2.2 Plant material

The SCR were collected from Binchuan County, Dali City, Yunnan Province, China, and authenticated by Professor Hongzhe Li of the Yunnan University of Chinese Medicine. A voucher specimen (D2021-001) has been deposited at the Yunnan Key Laboratory of Southern Medicinal Utilization, located at Yunnan University of Chinese Medicine in Kunming, China.

### 2.3 Extraction and isolation

The SCR (29.5 kg) was extracted five times with 85% EtOH by heating under reflux. The combined EtOH residue was suspended in water and then extracted with ethyl acetate (EtOAc, 977.5 g). The aqueous extract (600 g) was separated on D101 macroporous absorption resin chromatography, eluted with EtOH/H_2_O gradients (0:10, 5:10, 8.5:10) to obtain a 50% EtOH fraction (211.3 g) and an 85% EtOH fraction (20.8 g). The 85% EtOH fraction was further separated on silica gel CC (200–300 mesh) using a gradient mixture of CH_3_Cl/MeOH (from 1:0 to 0:1) to afford eight fractions (Fr. 1-Fr. 8). Fr. 2 (100.8 mg) was subjected to RP-C18 gel chromatography with gradient elution of MeOH/H_2_O (10%–60%) to yield five subfractions (Fr.2.1-Fr.2.5). Fr.2.3 was further purified by a Sephadex LH-20 column eluted with MeOH to yield compound 1 (3.5 mg). Fr. 3 (950.4 mg) was separated by MCI column chromatography with gradient elution of MeOH/H_2_O (10%–60%) to give six fractions (Fr.3.1-Fr.3.6). Fr.3.2 was further purified by a Sephadex LH-20 column eluted with CH_3_Cl/MeOH (1:1) to obtain compound 4 (100.0 mg). Fr.3.5 was further purified by a Sephadex LH-20 column eluted with MeOH to yield compound 22 (5.5 mg). Fr. 4 (670.8 mg) was subjected to RP-C18 gel chromatography with gradient elution of MeOH/H_2_O (10%–60%) to yield five subfractions (Fr.4.1-Fr.4.5). Fr.4.3 was further purified by a Sephadex LH-20 column eluted with MeOH to yield compounds 14 (10.5 mg), 15 (10.5 mg), and 16 (10.5 mg). Fr.4.5 was further purified by HPLC using an isocratic elution of ACN/H_2_O (25:75) to obtain compounds 2 (4.5 mg) and 7 (9.5 mg). Fr. 5 (560.5 mg) was further separated by MCI column chromatography with gradient elution of MeOH/H_2_O (10%–60%) to give six fractions (Fr.5.1-Fr.5.6). Fr.5.1 was further purified by HPLC using an isocratic elution of ACN/H_2_O (30:70) to obtain compounds 12 (15.0 mg) and 13 (15.0 mg). Fr.5.2 was subjected to silica gel CC (200–300 mesh) with an isocratic elution of petroleum ether/CH_3_Cl/MeOH (5:1) and was finally purified by a Sephadex LH-20 column eluted with MeOH to yield compounds 21 (3.5 mg) and 23 (2.5 mg). Fr.5.4 was further purified by a Sephadex LH-20 column eluted with MeOH and was finally purified by HPLC using an isocratic elution of ACN/H_2_O (23:77) to obtain compounds 18 (20.5 mg), 19 (7.5 mg), and 20 (5.8 mg). Fr. 6 (780.5 mg) was further separated by MCI column chromatography with gradient elution of MeOH/H_2_O (10%–60%), followed by purification on a Sephadex LH-20 column eluted with MeOH, and was finally purified by HPLC using an isocratic elution of ACN/H_2_O (28:72) to obtain compounds 24 (7.2 mg) and 25 (7.5 mg).

The ethyl acetate fraction was separated on silica gel CC (200–300 mesh) using a gradient mixture of petroleum ether and acetone (ranging from 1:0 to 0:1) as the eluent, yielding eight fractions (Fr. 1-Fr. 8). Fr. 3 (105.5 g) was then subjected to RP-C18 gel chromatography with gradient elution of MeOH/H_2_O (30%–80%), resulting in eight subfractions (Fr.3.1-Fr.3.8). Fr.4.2 was further purified by elution on a Sephadex LH-20 column using MeOH as the solvent, yielding compound 3 (950.0 mg). Fr.4.4 was subjected to silica gel CC (200–300 mesh) with isocratic elution using a mixture of petroleum ether and acetone (3:1). It was then further purified by elution on a Sephadex LH-20 column using MeOH as the solvent, yielding compound 26 (3.5 mg). Fr.4.6 was further purified by elution on a Sephadex LH-20 column using MeOH as the solvent, resulting in the isolation of compound 8 (10.0 mg). Fr. 4 (135.5 g) was also subjected to RP-C18 gel chromatography with gradient elution of MeOH/H_2_O (30%–80%), yielding eight subfractions (Fr.4.1-Fr.4.8). Fr.4.3 was subjected to silica gel CC (200–300 mesh) with isocratic elution using a mixture of petroleum ether and acetone (2:1). It was then further purified by elution on a Sephadex LH-20 column using MeOH as the solvent, yielding compounds 9 (7.0 mg), 11 (10.5 mg), and 17 (20.0 mg). Fr.4.6 was further purified by HPLC using an isocratic elution of ACN/H_2_O (30:70), resulting in the isolation of compounds 5 (8.5 mg) and 6 (7.5 mg). Finally, Fr.4.8 was further purified by elution on a Sephadex LH-20 column using MeOH as the solvent, yielding compound 10 (5.5 mg).

### 2.4 Network pharmacological analysis of SCR

First, we used the compounds obtained from the above isolation as active ingredients. Subsequently, we obtained the canonical SMILES representations of the compounds from PubChem (https://pubchem.ncbi.nlm.nih.gov/). Potential targets were identified by inputting these representations into Swiss Target Prediction (http://www.swisstargetprediction.ch/). The analysis was confined to *Homo sapiens*, utilizing a probability threshold of greater than 0.01 as the screening criterion.

Genes linked to inflammation were obtained from the Uniprot database (https://www.uniprot.org/) and the Drug Bank database (https://go.drugbank.com/). In these databases, a search for “breast cancer” was conducted, with “*Homo sapiens*” specified as the target species.

Enrichment analyses for Gene Ontology (GO) and Kyoto Encyclopedia of Genes and Genomes (KEGG) pathways were performed using Metascape (https://metascape.org). The GO enrichment analysis included BPs, MFs, and CCs. The results were subsequently imported and visualized on a bioinformatics platform (https://www.bioinformatics.com.cn/) to examine signaling pathways associated with critical molecular biological processes and key targets. The development of a component-target-pathway graph involves the integration of the top 10 pathways identified as enriched by the KEGG with the incorporation of relevant targets into the Cytoscape platform.

### 2.5 Cell culture and regents

The KGN cell were purchased from the Cell Bank of Chinese Academy of Science (Shanghai, China). They were cultured in DMEM/F-12 media containing 10% FBS, 100 U/mL penicillin, and 0.1 mg/mL streptomycin, and placed in a 37°C incubator with 95% CO_2_ and 5% air.

### 2.6 Cell viability

Plate KGN cells in the logarithmic growth phase at a density of 3 × 10^3^ cells per well in 96-well plates, and incubate overnight. Once the cells were fully attached, add compounds 1–26 (40 μM) in each well, and continue to culture for 48 h. Add 10% CCK-8 reagent to each well, incubate at 37°C for 1 h, and measure the absorbance at 450 nm.

### 2.7 The effect of compounds 1–26 on E2 biosynthesis

KGN cells were inoculated into 24-well plates at a density of 1 × 10^4^ cells/well and incubated overnight. Once the cells had fully attached, compounds 1-26 (40 μM), forskolin, and letrozole were added to each well. After the cells were cultured for an additional 24 h, testosterone (Test, 10 nM) was added to each well and cultured for another 24 h. The cell supernatant was collected and stored at −20°C. E2 level was detected in accordance with the manufacturer’s protocol (Elabscience, Wuhan, China).

### 2.8 qRT-PCR

The KGN cells were inoculated on 6-well plates at a density of 2 × 10^4^ cells/well, and incubated overnight. After 48 h treatment with different dose of compounds 1 and 2, total RNA was extracted using TRIzol regant, and was reverse transcribed into cDNA using RT-PCR SuperMix. Equal amounts of cDNA from each group were used to perform RT-qPCR experiments using TransStart Tip Green qPCR SuperMix. The following primer pairs were used: P Ⅰ.1 Forward (5′-CCA​CCC​ATG​GCA​AAT​TCC​ATG​GCA-3′) and Reverse (5′-GGT​GGA​CCT​GAC​CTG​CCG​TCT​AGA-3′), P Ⅰ.3 Forward (5′-GTC​TAA​AGG​AAC​CTG​AGA​CTC​TAC-3′) and Reverse (5′-ACG​ATG​CTG​GTG​ATG​TTA​TAA​TGT-3′), P Ⅰ.4 Forward (5′-CAC​TGG​TCA​GCC​CAT​CAA-3′) and Reverse (5′-ACG​ATG​CTG​GTG​ATG​TTA​TAA​TGT-3′), P Ⅱ Forward (5′-CCC​TTT​GAT​TTC​CAC​AGG​AC-3′) and Reverse (5’ -CCC​TTT​GAT​TTC​CAC​AGG​AC-3′), Aro Forward (5′-ATC​CTC​AAT​ACC​AGG​TCC​TGG​C-3′) and Reverse (5′-AGA​GAT​CCA​GAC​TCG​CAT​GAA​TTC​T-3′), and glyceraldehyde 3-phosphatedehydrogenase (GAPDH) Forward (5′-CCA​CCC​ATG​GCA​AAT​TCC​ATG​GCA-3′) and Reverse (5′-GGT​GGA​CCT​GAC​CTG​CCG​TCT​AGA-3′). The relative expression level of Aro and its promoters were calculated using the 2^−ΔΔCT^ method and normalized to the house-keeping gene GAPDH.

### 2.9 Western blotting

KGN cells were seed on 6-well plates at a density of 2 × 10^4^ cells/well and incubated overnight. After 48 h of treatment with different doses of compounds 1 and 2, the cells were washed three times with precooled PBS and lysed in RIPA buffer. Total protein content was detected using the BCA assay kit. Equal amounts of protein from each group were loaded into SDS-PAGE and transferred onto PVDF membranes. Each membrane was incubated with corresponding primary antibody overnight, with GAPDH serving as the control.

### 2.10 Molecular docking

Perform virtual validation of the core target using AutoDock Vina software. The structure of the core target protein is downloaded from PDB (https://www.rcsb.org/). Use PyMOL software to remove water molecules and ligands from the protein. A binding energy of less than −5 kcal/mol indicates good affinity between the compound and the target. Visualization is conducted using PyMOL and proteins.plus (https://proteins.plus/).

### 2.11 Statistical analysis

All data are presented as mean ± standard deviation. Data were analyzed using one-way analysis of variance with GraphPad Prism 7.0 software. A significance level of *P* < 0.05 was considered statistically significant.

## 3 Results

### 3.1 Structural elucidation

**FIGURE 1:**
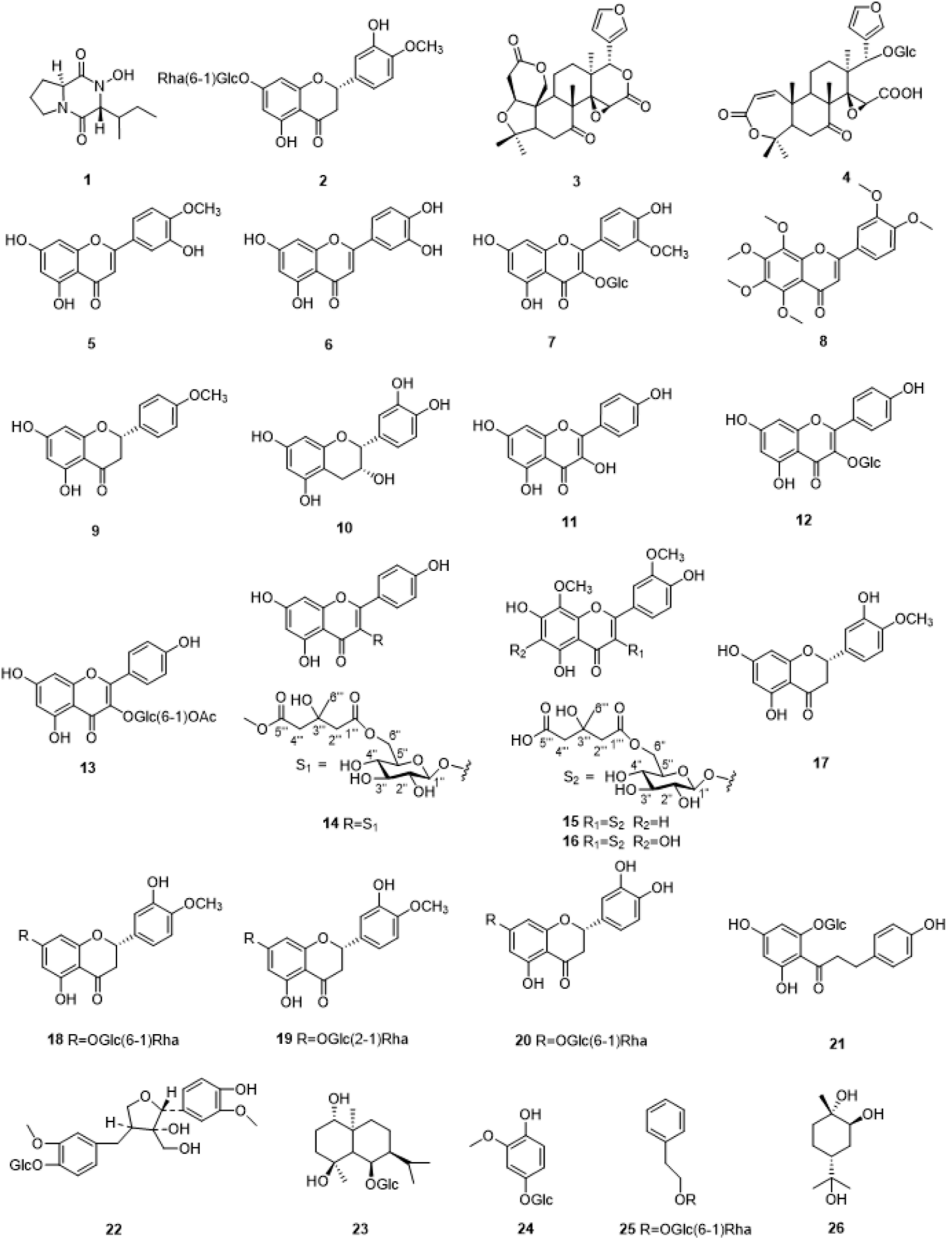
The chemical structures of compounds1-26 isolated from SCR.

26 compounds were isolated from SCR (Figure 1), including Callyspongidipeptide A (Calp, 1) (Yang et al., 2009), Hesperetin 7-*O*-*β*-*D*-glucopyranoside (Hesp, 2) (Shimoda et al., 2008), Limonin (3) (Yang et al., 2024), Obacunone 17-*O*-*β*-*D*-glucopyranoside (4) (Sawabe et al., 1999), Diosmetin (5) (Zuo et al., 2013), Luteolin (6) (Zhu et al., 2019), Isoquercetin (7) (Kazuma et al., 2003), Nobiletin (8) (Dong et al., 2018), Isosakuranetin (9) (Zhang et al., 2006), Epicatechin (10) (Li et al., 2023), Kaempferol (11) (Li et al., 2008), Kaempferol-3-*O*-*β*-*D*-glucopyranoside (12) (Hari et al., 2003), Kaempferol-3-*O*-(6″-*O*-acetyl)-*β*-*D*-glucopyranoside (13) (Foo et al., 2000), Kaempferol-3-*O*-glucosyl-6″-*O*-pentadionic acid (14) (Min et al., 2021), 5, 7,4′-trihydroxy-8,3′-dimethoxyflavone-3-*O*-6″-3-hydroxyl-3-methylglutaroyl)-*β*-*D*-glucop-yranoside (15) (Deng et al., 2020), 5, 7, 4′-trihydroxy-6, 8, 3′-dimethoxyflavone-3-*O*-6″-3-hydroxyl-3-methylglutaroyl)-*β*-*D*-glucopyranoside (16) (Chen et al., 2019), Hesperitin (17) (Liu et al., 2016), Hesperidin (18) (Zou et al., 2020), Neohesperidin (19) (Liu et al., 2019), Eriodictioside (20) (Peng et al., 2021), Phlorizin (21) (Wang M. M. et al., 2012), 8-Hydroxypinoresinol-4′-*O*-*β*-*D*-Glucopyranoside (22) (Kitajima et al., 2003; Wang Q. H. et al., 2012), Pumilaside A (23) (Wang M. M. et al., 2012), 4-hydroxy-2-methoxyphenol-1-*O*-*β*-*D*-glucopyranoside (24) (Su et al., 2012), Phenylethyl-rutinoside (25) (Su et al., 2012), Trans-p-menthane-1*α*, 2*β*, 8-triol (26) (Jung et al., 2001). These compounds were identified by comparison of their spectroscopic data with those reported in the literature (Supplementary Material).

### 3.2 Spectroscopic data for compounds Calp and Hesp

#### 3.2.1 Callyspongidipeptide A (Calp, 1)

White Powder, ^1^H-NMR (CD_3_OD, 400 MHz), *δ*: 4.22 (1H, t, *J* = 7.8 Hz, H-9), 4.08 (1H, t, *J* = 2.3 Hz, H-6), 3.60-3.53, 3.52-3.49 (2H, m, H-3), 2.35-2.02, 1.98-1.91 (2H, m, H-5), 2.20-2.16 (1H, m, H-10), 2.06-2.02, 1.98-1.91 (2H, m, H-4),1.49-1.43, 1.37-1.31 (2H, m, H-11), 1.07 (3H, t, *J* = 6.9 Hz, H-12), 0.95 (3H, t, *J* = 6.9 Hz, H-13); ^13^C-NMR (CD_3_OD, 100 MHz), *δ*: 172.4 (s, C-1), 46.2 (t, C-3), 23.2 (t, C-4), 29.6 (t, C-5), 61.3 (d, C-6), 167.6 (s, C-7), 60.0 (d, C-9), 37.1 (d, C-10), 25.5 (d, C-11), 15.5 (q, C-12), 12. 6 (q, C-13).

#### 3.2.2 Hesperetin 7-*O*-*β*-*D*-glucopyranoside (Hesp, 2)

Yellow powder, ^1^H-NMR (DMSO-d_6_, 400 MHz) *δ*: 12.0 (1H, s, 5-OH), 9.1 (1H, s, 3′-OH), 6.95 (1H, d, *J* = 8.6 Hz, H-5′), 6.93 (1H, s, H-2′), 6.91 (1H, d, *J* = 8.6 Hz, H-6′), 6.14 (1H, d, *J* = 2.3 Hz, H-8), 6.12 (1H, d, *J* = 2.3 Hz, H-6), 5.52 (1H, dd, *J* = 12.2, 3.1 Hz, H-2), 3.77 (3H, s, H-OCH_3_), 3.42 (1H, dd, *J* = 17.4, 12.0 Hz, H-3a), 2.77 (1H, dd, *J* = 17.4, 3.0 Hz, H-3b); ^13^C-NMR (DMSO-d_6_, 100 MHz) *δ*: 78.5 (C-2), 42.1 (C-3), 197.1 (C-4), 162.7 (C-5), 96.5 (C-6), 165.2 (C-7), 95.5 (C-8), 162.7 (C-9), 103.3 (C-10), 130.9 (C-1′), 114.1 (C-2′), 146.5 (C-3′), 148.0 (C-4′), 112.0 (C-5′), 117.9 (C-6′), 55.7 (C-OCH_3_), 99.6 (C-1″), 73.0 (C-2″), 77.1 (C-3″), 69.5 (C-4″), 76.3 (C-5″), 60.6 (C-6″).

### 3.3 Collection targets of breast cancer

**FIGURE 2:**
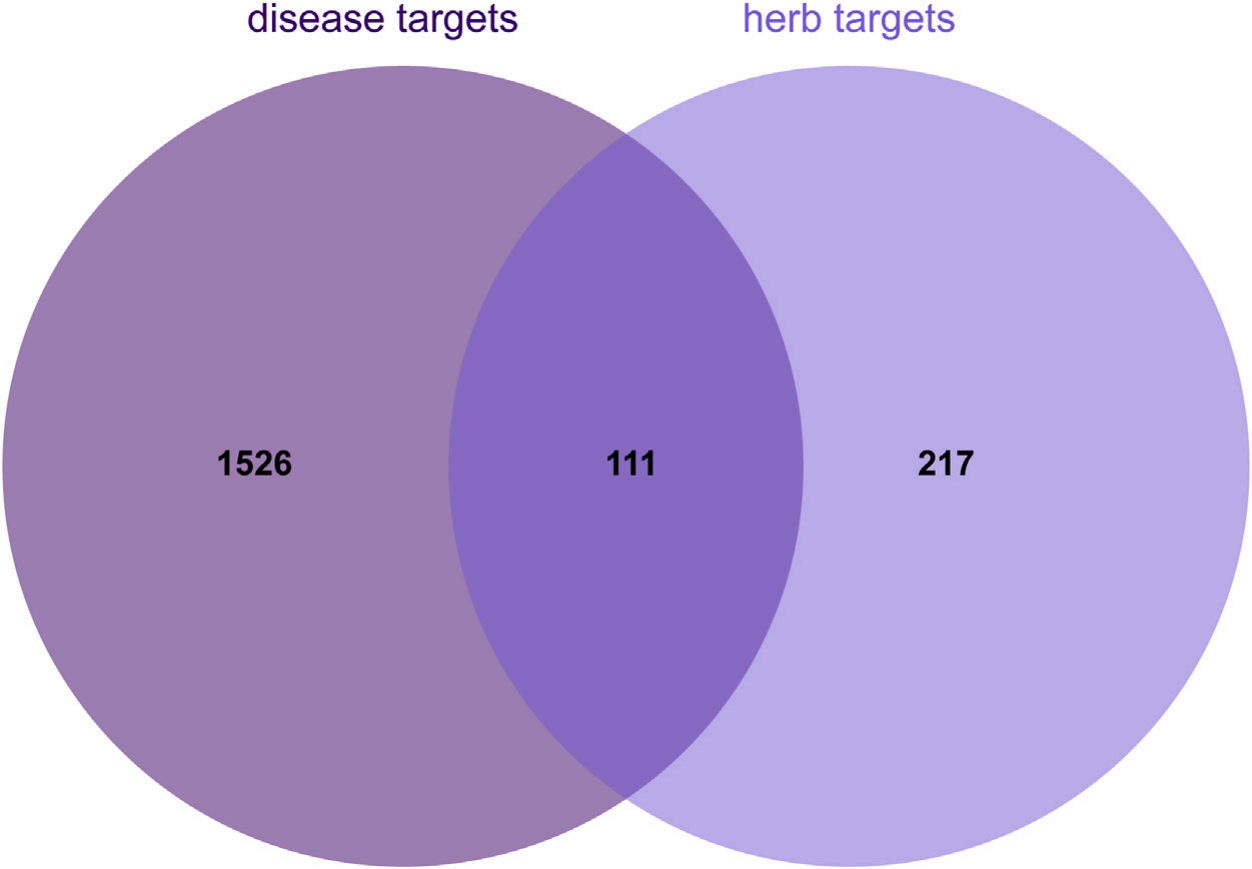
Venn diagram illustrating the overlap between herb and disease targets.

By using the online platform Draw Venn Diagram to find the intersection between the screened ingredient targets and disease targets, and plotting a Venn diagram, we obtained 174 targets for the therapeutic effect of SCR in breast cancer (Figure 2).

### 3.4 GO and KEGG pathway enrichment analysis

**FIGURE 3:**
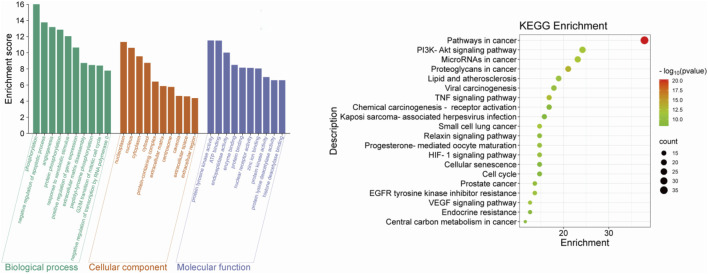
The top 10 GO terms and top 20 KEGG pathway of herb genes.

**FIGURE 4:**
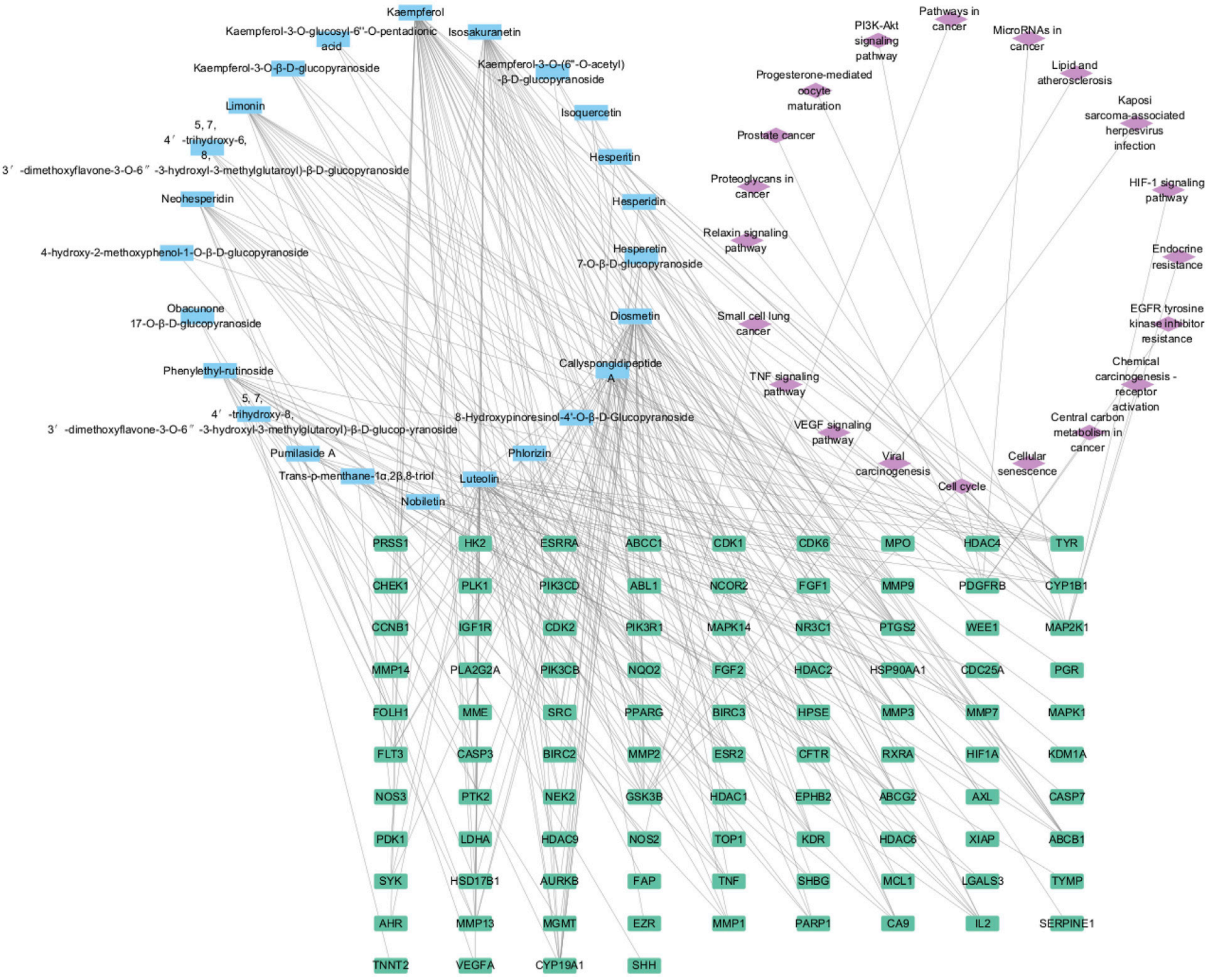
The component-target-pathway network.

The findings from the enrichment analysis revealed a total of 164 BPs, 314 CCs, 211 MFs, and 188 KEGG pathways, with a significance level of *P* < 0.01. The illustration presents enriched GO, KEGG categories and component-target-pathway (Figures 3, 4). Molecular functions encompass various activities such as phosphorylation, negative regulation of apoptotic process, and angiogenesis, among others. The cellular components identified nucleoplasm, nucleus, and cytoplasm. Additionally, the biological processes involve responses to protein tyrosine kinase activity, ATP binding, and the endopeptidase activity, among other functions. The ten most prevalent KEGG pathways are presented, which include pathways related to cancer, Phosphatidylinositol 3-Kinase/Akt (PI3K/AKT) signaling pathway, Lipid and atherosclerosis, and Viral carcinogenesis, among others (Supplementary Table S1).

Through KEGG and GO enrichment analyses, it has been found that the anti-breast cancer effect of SCR primarily influences the phosphorylation of the PI3K/AKT pathway, thereby affecting the progression of breast cancer. Follicle-stimulating hormone can promote the proliferation and differentiation of granulosa cells in preantral and antral follicles, activate Aro within granulosa cells, convert androgen produced by theca cells into estrogen, and increase the level of estrogen in the body. Most breast cancer cells have estrogen receptors on their surface, and E2, as a major form of estrogen, can bind to these receptors, activating intracellular signaling pathways and thereby promoting the growth and proliferation of breast cancer cells. Therefore, we further investigated the effect of citrus seed on E2 secretion to explore its mechanism of action in anti-breast cancer. Therefore, we next explore whether SCR regulates estrogen biosynthesis by modulating the PI3K-AKT pathway.

### 3.5 Calp and Hesp inhibit E2 biosynthesis in KGN cells

**FIGURE 5:**
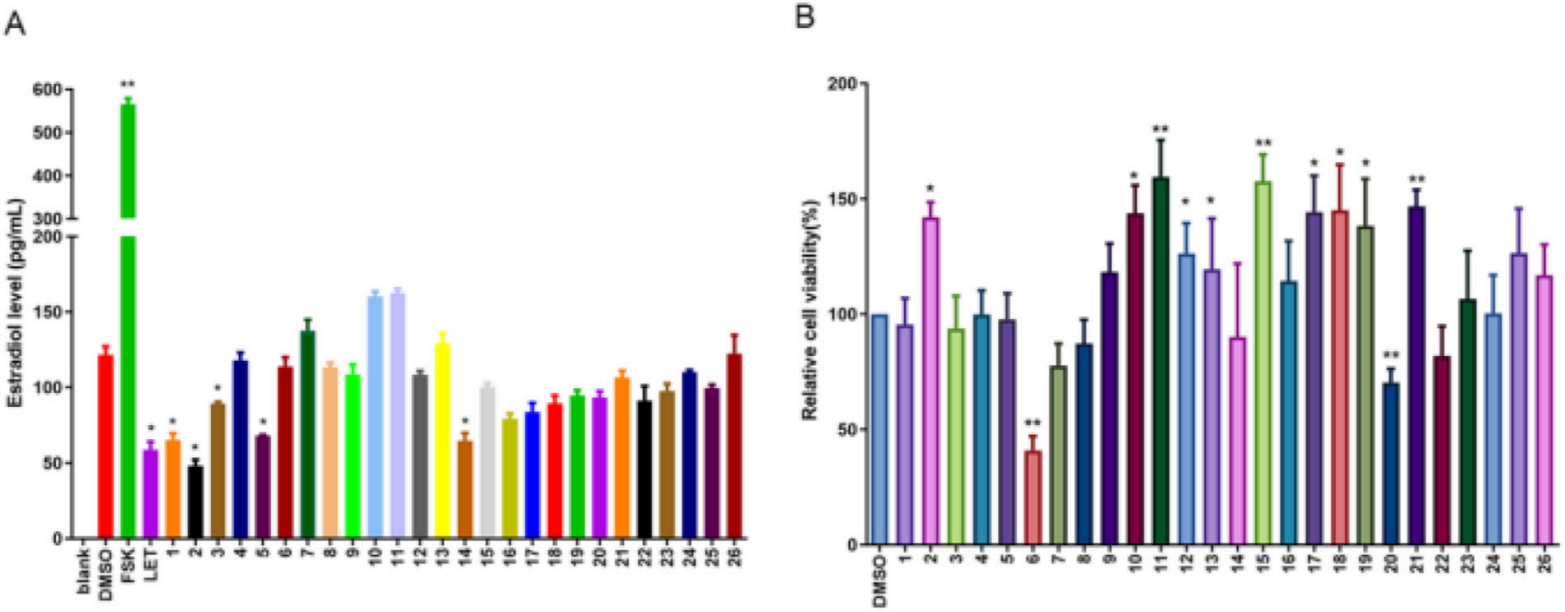
The effect of compound 1-26 on E2 biosynthesis in KGN cells. The E2 levels in KGN cells after 48 h compound 1-26 treatment **(A)**. The effect of compound 1-26 on KGN cell proliferation **(B)**. *p < 0.05, compared with DMSO group; **p < 0.01, compared with DMSO group.

**FIGURE 6:**
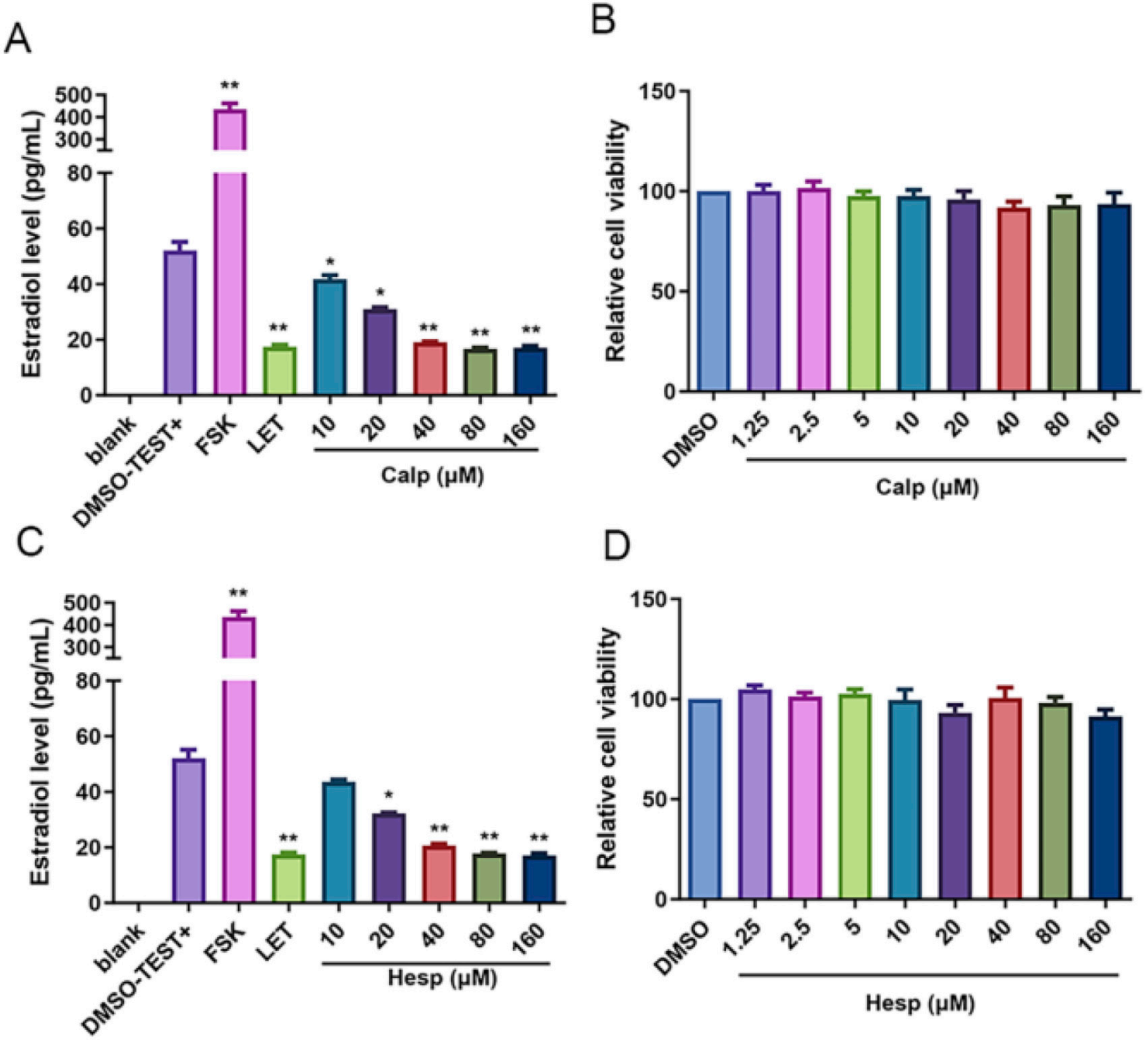
Calp and Hesp exert inhibitory effect on E2 synthesis in a dose-dependent manner. The impact of various concentrations of Calp **(A)** and Hesp **(C)** on E2. The impact of various concentrations of Calp **(B)** and Hesp **(D)** on KGN cells proliferation. **p < 0.01, compared with DMSO group.

Aro, as a pivotal rate-limiting enzyme in the E2 synthesis pathway, plays an integral role in the conversion of androgens into E2. It is notably expressed in KGN cells, a human granulosa-like tumor cell line that is commonly used for studying estrogen synthesis due to its high Aro activity. However, these cells lack the necessary substrates for endogenous estrogen production. We supplemented KGN cells with Test, providing the substrate necessary for estrogen synthesis, to overcome this limitation and establish a robust *in vitro* screening system for Aro inhibitors. Upon incubation with Test, as depicted in Figure 5A, we observed a significant reduction in E2 biosynthesis when KGN cells were treated with Calp and Hesp. These compounds effectively diminished the conversion of Test into E2, suggesting the existence of a potent inhibitory effect on Aro activity. Importantly, the CCK8 proliferation assays conducted after 48 h of treatment revealed that at the tested concentrations, Calp and Hesp did not induce significant cytotoxicity to KGN cells (Figure 5B). This finding indicates that the inhibition of E2 synthesis by these compounds is not due to cell death. Further analysis demonstrated that Calp and Hesp exerted their anti-Aro effects in a dose-dependent manner (Figures 6A,C). The higher the concentration of these compounds, the more pronounced the inhibition of E2 synthesis, and none of the concentrations used were cytotoxic to KGN cells (Figures 6B,D). This dose dependency underscores the potential of Calp and Hesp as selective Aro inhibitors.

### 3.6 Calp and Hesp inhibit Aro promoter expression

**FIGURE 7:**
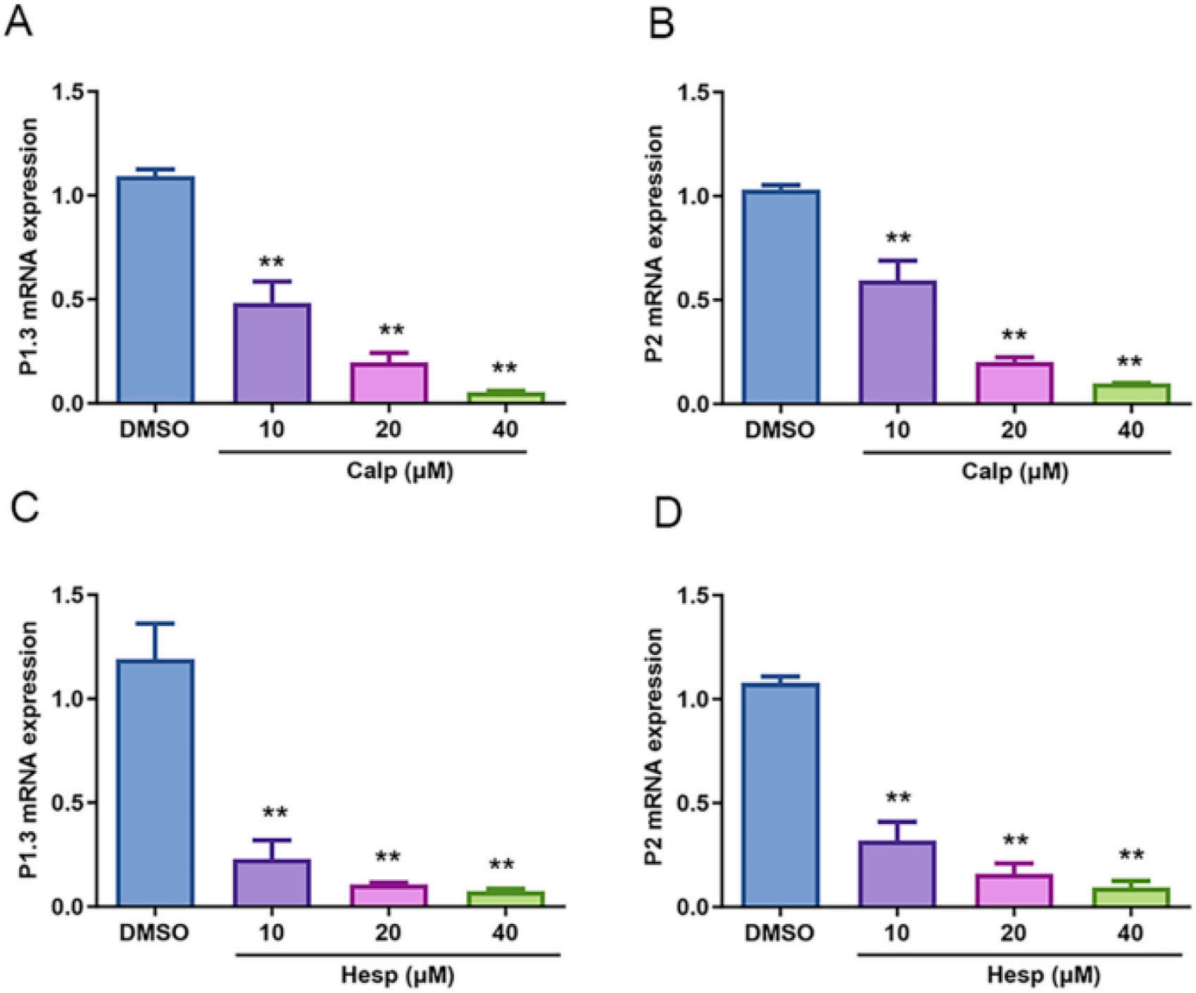
Calp and Hesp downregulate the mRNA levels of PI.3 and P2. Different concentrations of Calp inhibited the mRNA expression of PI.3 **(A)** and P2 **(B)**. Different concentrations of Hesp inhibited the mRNA expression of PI.3 **(C)** and P2 **(D)**. **p < 0.01, compared with DMSO group.

The regulation of Aro expression is intricately linked to its promoter, which varies across different tissues, with heightened expression observed in the ovary, placenta, adipose tissue, and breast. Specifically, PI.3, which is predominantly expressed in adipose tissue and mammary glands, showed a notable decrease in expression with increasing concentrations of Calp and Hesp. Similarly, P2, which is known for its expression in the ovary and hormone-related cancers, was also significantly inhibited. Given this tissue-specific regulation, we further investigated how Calp and Hesp influence Aro expression at the transcriptional level. The treatment of KGN cells with Calp and Hesp (10–40 μM) over a 48 h period revealed the significant downregulation of PI.3 and P2 mRNA (Figures 7A–D). This targeted promoter suppression indicates that Calp and Hesp exert their effects by regulating Aro transcriptional activity.

### 3.7 Calp and Hesp inhibit Aro expression

**FIGURE 8:**
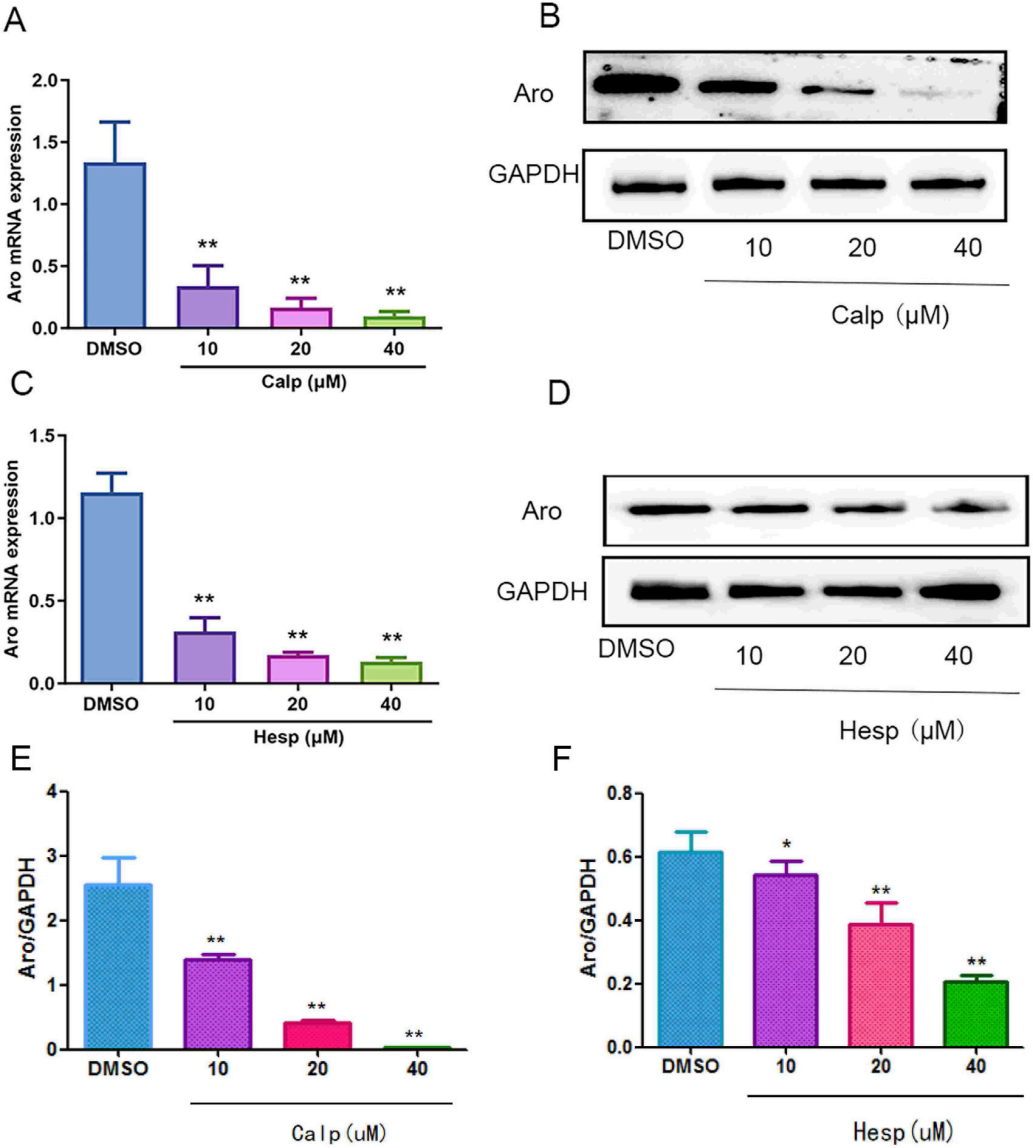
The effects of Calp and Hesp on the expression level of Aro. Calp **(A)** and Hesp **(C)** downregulate the mRNA level of Aro. Calp **(B)** and Hesp **(D)** inhibit the expression of Aro in KGN cells. Calp **(E)** and Hesp **(F)** Gray value analysis of Western blotting **p < 0.01, compared with DMSO group.

Continuing our investigation into the effects of Calp and Hesp on Aro regulation, we assessed their impact on both mRNA and protein levels of Aro in KGN cells. Our qRT-PCR analysis, as shown in Figures 8A,C, demonstrated a clear dose-dependent suppression of Aro mRNA expression by both Calp and Hesp. As shown in Figures 8B,D–F, confirmed that treatment with Calp and Hesp significantly diminished Aro protein expression in KGN cells. These results further demonstrate that Calp and Hesp inhibit Aro expression by regulating Aro promoters.

### 3.8 Molecular docking analysis and verification

**FIGURE 9:**
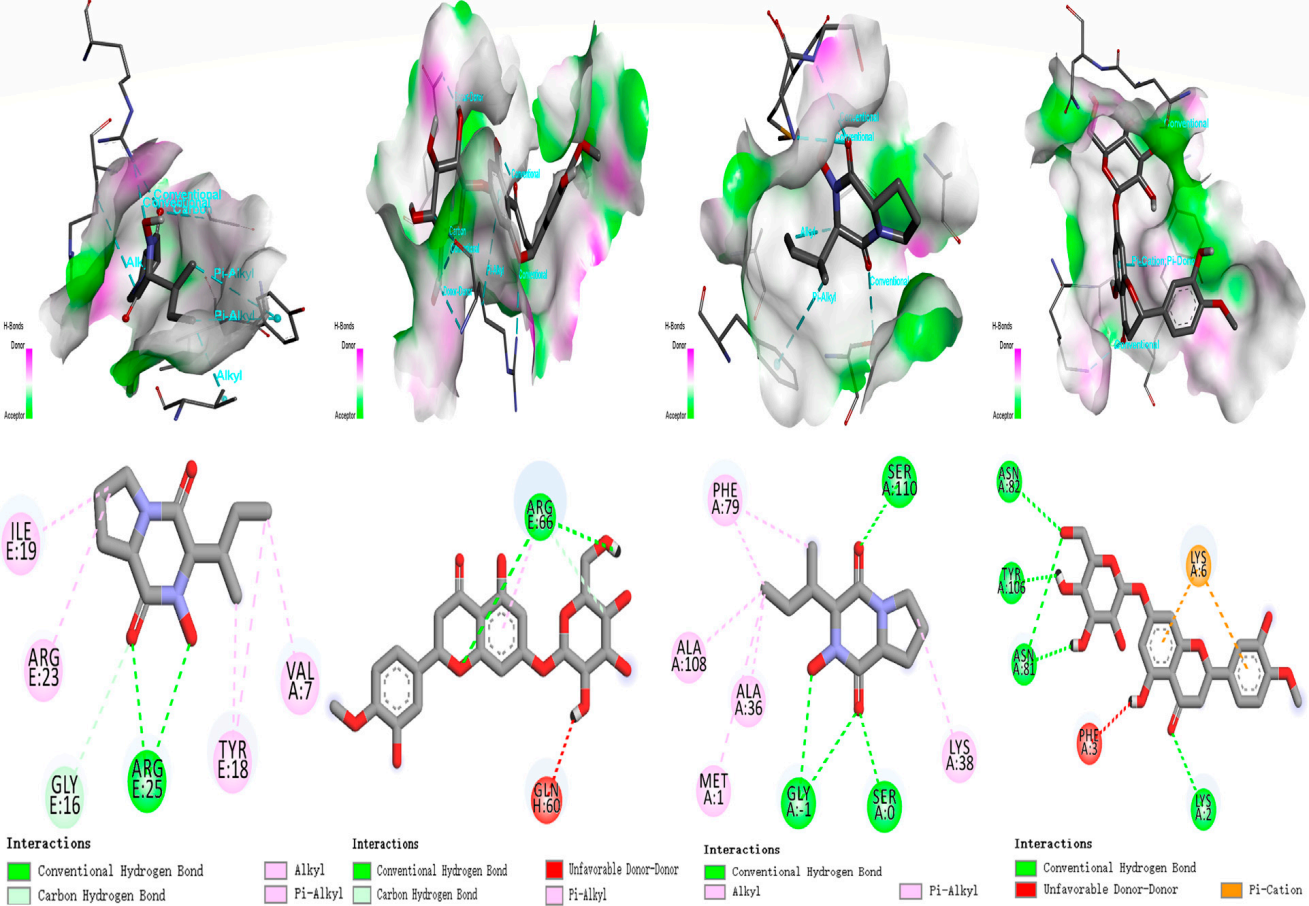
Molecular docking result for the targets. **(A)** Calp; Hesp - PI3K (6hog). **(B)** Calp; Hesp - AKT (2 × 18).

**FIGURE 10:**
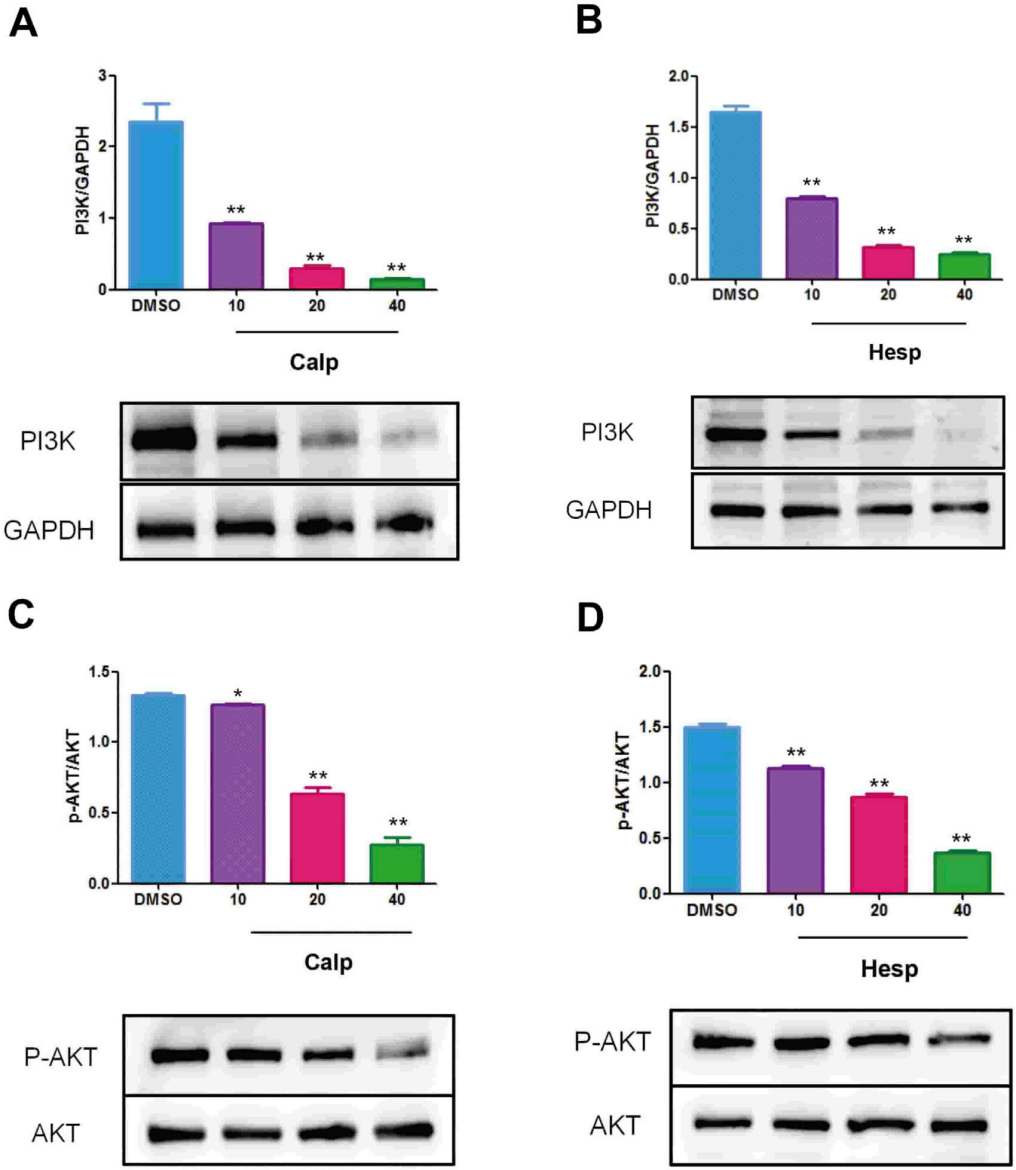
Calp **(A)** and Hesp **(B)** inhibit the expression of PI3K in KGN cells. Calp **(C)** and Hesp **(D)** inhibit the expression of p-AKT in KGN cells. **p < 0.01, compared with DMSO group.

The receptor proteins were PI3K (6hog) and AKT (2 × 18), and their 3D structure files were downloaded from the PDB database. PyMOL 2.3.0 software was used to check the protein structure for docking. The ligand small molecule is Calp and Hesp. Employ Auto Dock Vina (version 1.1.2) to perform docking of the compounds with the target. Calp forms hydrogen bonds with the Tyr, Gly, Arg, and Val amino acid residues in PI3K, with bond lengths of 7, 16, 18, 19, 23, and 25. Hesp forms hydrogen bonds with the Phe, Asn, Lys, and Glu amino acid residues in PI3K, with bond lengths of 2, 6, 79, 81, 82, and 100. Calp forms hydrogen bonds with the Ser, Gly, Ala, Met, and Phe amino acid residues in AKT, with bond lengths of 1, 36, 38, 79, 108, and 110. Hesp forms hydrogen bonds with the Arg, Thr, and Lys amino acid residues in AKT, with bond lengths of 58, 66, 91, and 111. All molecular docking binding energies are less than −5 kJ/mol, indicating strong binding free energy capabilities. Further experimental validation revealed that compounds Calp and Hesp inhibit the PI3K and phosphorylation of AKT (p-AKT). We propose that SCR inhibits estrogen biosynthesis via modulating the PI3K-aromatase pathway, thereby mitigating breast cancer progression (Figures 9, 10; Supplementary Table S2).

## 4 Discussion

Breast cancer is a malignant tumor that originates from the epithelial cells of the mammary gland. Estrogen is a steroid hormone in the human body that plays an important role in reproduction, immunity, bones, the cardiovascular system, and the central nervous system (Tang et al., 2019; Pater et al., 2020). There are three main types of estrogen, with the most active being E2, followed by estrone, and the least active being estriol. Their biological effects are primarily exerted by binding to estrogen receptors, thereby activating their transcriptional or non-transcriptional activities. With the advancement and development of society and the economy, the incidence and mortality rates of many diseases such as breast cancer, ovarian cancer, endometrial cancer, cardiovascular diseases, osteoporosis, and parkinson’s disease have shown a sharp upward trend. The onset, progression, and deterioration of these diseases are all related to the disorder of estrogen synthesis and metabolism. According to the 2020 global cancer data, the incidence rate of breast cancer in women has surpassed that of lung cancer, becoming the most common type of cancer in diagnostics, with 2,300,000 new cases globally, accounting for 11.7% of all new cases. In recent years, with the development of molecular biology, breast cancer is typically classified based on molecular features in clinical practice. According to the molecular characteristics of breast cancer, it can be roughly divided into three types: ER+, HER2+, and TNBC types. Clinical research has found that approximately 70% of breast cancers exhibit hormone receptor positivity, and the mainstream treatment for this type of breast cancer is endocrine therapy. Clinically, it has been proven that endocrine therapy is effective in treating ER + breast cancer. Aromatase inhibitors are the primary medications used in endocrine therapy. Currently, three generations of aromatase inhibitors have been developed. The first generation comprises aminoglutethimide. The second generation encompasses fadrozole, 4-hydroxyandrostenedione, exemestane, and formestane, among others. The third generation includes anastrozole and letrozole, among other compounds (Bell et al., 2020). However, these drugs directly target Aro, lack tissue specificity, about 50% of adverse effects have also attracted increasing attention. The side effects such as hot flashes, insomnia, mood changes, hyperlipidemia, and osteoporosis, are mainly mild to moderate. Approximately 25% of patients discontinue treatment due to intolerance (Zhou et al., 1997). Therefore, there is a need to identify compounds that can inhibit Aro expression at the transcriptional or post-translational level.

In this study, we examined the effects of 26 compounds isolated from SCR on E2 biosynthesis in KGN cells, found that Calp and Hesp significantly inhibited E2 biosynthesis. CCK8 assays confirmed that the doses of Calp and Hesp did not have cytotoxic effects on KGN cells. Aro is a key rate-limiting enzyme in E2 biosynthesis. Aro exons Ⅱ-Ⅹ are located in the coding region, whereas exon Ⅰ is situated in the 5′-untranslated region. Exon Ⅰ plays a crucial role in the tissue-specific expression of the Aro. The upstream region of the Aro gene contains some promoters that regulate the tissue-specific expression of Aro. These tissue-specific promoters include promoter PⅠ.1 (primarily expressed in placenta), PⅠ.3 (expressed in adipose tissue and mammary glands), PⅠ.4 (extragonadal sites, such as the main promoter in adipose tissue), and P2 (expressed in adipose tissue, breast cancer, ovarian cancer, endometrial cancer, containing cAMP response elements). In premenopausal women, under the stimulation of gonadotropic hormones, Aro in human ovarian tissue is primarily produced by the ovarian-specific promoter P2. Therefore, we further investigated the effects of Calp and Hesp on the Aro promoter. The qRT-PCR results indicated that Calp and Hesp significantly inhibited PI.3 and P2 expression in a dose-dependent manner. In addition, we found Calp and Hesp decreased Aro mRNA expression. After 48 h Calp and Hesp treatment, WB experimental results showed Aro expression was significantly downregulated. Utilizing network pharmacology and molecular docking techniques, the therapeutic targets and pathways of SCR in treating breast cancer have been elucidated. SCR potentially inhibits breast cancer by modulating core targets such as platelet-derived growth factor receptor, beta polypeptide, protein tyrosine kinase 2, and threonine kinase 1, which in turn affect pathways including the PI3K/AKT pathway, epidermal growth factor receptor pathway, and tumor necrosis factor pathway. Although compounds Calp and Hesp exhibited favorable docking scores with PI3K/AKT, their reliance on rigid conformation models and oversimplification of solvent effects and entropic contributions often fail to account for target flexibility and environmental effects, which may compromise prediction accuracy, thus necessitating further experimental validation (Paggi et al., 2024).

**FIGURE 11:**
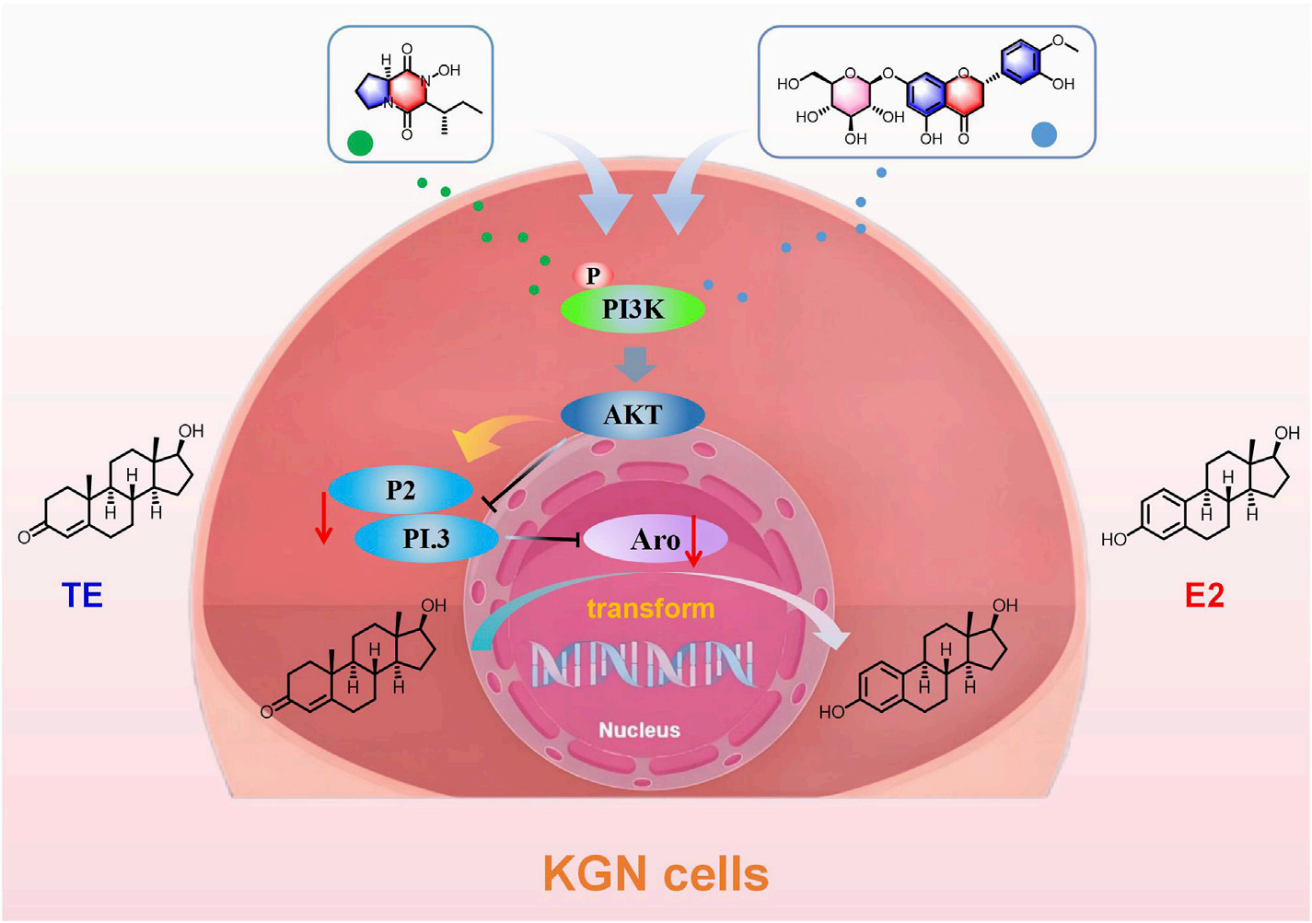
The mechanism of SCR inhibiting aromatase in plate KGN cells.

Aro is a key rate-limiting enzyme in the body that catalyzes the conversion of androgens to estrogens, playing a crucial role in the development of breast cancer. Targeting Aro has thus become an important therapeutic approach in the treatment of breast cancer. In this study, we isolated 26 compounds from SCR, mainly including limonoid and flavonoids. Compounds 1, 14, 15 and 16 were isolated from SCR for the first time. We found that Calp and Hesp inhibit Aro expression by selectively regulating the expression of its PI.3 and P2 promoters, ultimately suppressing the biosynthesis of estrogen (Figure 11).

SCR primarily contains flavonoids and limonoids. Some of these flavonoid compounds exhibit Pan-assayinterference compounds (PAINS) substructures characteristics. We further analyze whether Calp and Hesp belong to PAINS. PAINS often encompass certain reactive groups, such as Michael acceptors, quinones, and specific nitrogen-containing heterocycles, which are associated with non-specific reactivity and assay interference (Bolz et al., 2021). These structural features are indicative of compounds that may exhibit promiscuous binding and interference in various screening assays (Bolz et al., 2021). Calp, a cyclic dipeptide composed of cyclo-((S)-Pro-8-hydroxy-(R)-Ile), primarily contains a proline residue. Its structure is characterized by peptide bonds and hydroxyl groups, which typically engage in specific hydrogen bonding interactions with target proteins (Yang et al., 2009). Similarly, Hesp is a flavonoid with a structure that includes a flavanone skeleton substituted with hydroxyl groups and a *β*-*D*-glucopyranoside moiety (Shimoda et al., 2008). The hydroxyl groups and glycosidic linkages in this compound are generally involved in forming hydrogen bonds and other specific interactions with biological targets. Neither Calp nor Hesp possesses the typical PAINS features, such as the aforementioned reactive groups. Their structural motifs suggest a higher propensity for specific interactions with targets rather than non-specific assay (Bolz et al., 2021). In subsequent studies, we will further investigate the mechanisms by which Calp and Hesp inhibit aromatase through computational analysis (Magalhães et al., 2021), fluorescence quenching assays, thiol reactivity assays, surface plasmon resonance (Gao et al., 2025) and thermal shift assays (Caputo et al., 2025).

The discovery of natural compounds like Calp and Hesp from SCR with potential anti-breast cancer properties presents a significant opportunity. Calp and Hesp did not show cytotoxic effects in preliminary assays, indicating a potential safe profile for SCR. Their effectiveness depends heavily on bioavailability, necessitating further studies on absorption, distribution, metabolism, and excretion characteristics. Further discover new aromatase inhibitors from traditional Chinese medicine to promote the treatment of breast cancer.

## 5 Conclusion

Our comprehensive investigation of the phytochemical composition of SCR led to the isolation of 26 distinct compounds. Network pharmacology analysis revealed that SCR may influence breast cancer development by modulating phosphorylation-related biological processes and the PI3K/AKT pathway. Notably, Calp and Hesp exhibited significant anti-aromatase activity, effectively inhibiting estrogen biosynthesis in KGN cells. Further analysis confirmed that SCR regulates estrogen biosynthesis via the PI3K/AKT pathway.

The identification of Calp and Hesp as potent natural Aro inhibitors highlights the therapeutic potential of bioactive compounds in SCR. This study underscores the value of integrating traditional knowledge with modern scientific methodologies, new approach for exploring the therapeutic potential of traditional Chinese medicine in breast cancer treatment.

## Data Availability

The datasets presented in this study can be found in online repositories. The names of the repository/repositories and accession number(s) can be found in the article/Supplementary Material.

## Abbreviations

Calp, Callyspongidipeptide A; SCR, The seeds of *Citrus reticulata* Blanco (Rutaceae); TNBC, Triple-negative breast cancer; Aro, Aromatase; E2, Estradiol; ER+, Estrogen receptor–positive; HER2+, Human epidermal growth factor receptor 2–positive; Hesp, Hesperetin 7-*O*-*β*-*D*-glucopyranoside; GO, Gene Ontology; KEGG, Kyoto Encyclopedia of Genes and Genomes; GAPDH, Glyceraldehyde 3-phosphatedehydrogenase; PI3K/AKT, Phosphatidylinositol 3-Kinase/Akt; PAINS, Pan-assayinterference compounds.
