# Supplementary material for: Two natural products from the seeds of Citrus reticulata Blanco (Rutaceae) inhibit estrogen biosynthesis by regulating the PI3K-aromatase pathway
Source: Front Pharmacol. 2025 Jun 12;16:1583409. doi: 10.3389/fphar.2025.1583409 (PMC12198254; doi:10.3389/fphar.2025.1583409)
Supplement: Supplementary file 1 [file DataSheet1.pdf]

Contents of Supporting Information

Figure S1. ^{1}H NMR of Callyspongidipeptide A (1).. 1

Figure S2. ^{13}C NMR of Callyspongidipeptide A (1).. 1

Figure S3. ^{1}H NMR of Hesperetin 7-*O*-*β*-*D*-glucopyranoside (2).. 2

Figure S4. ^{13}C NMR of Hesperetin 7-*O*-*β*-*D*-glucopyranoside (2).. 2

Figure S5. ^{1}H NMR of Limonin (3).. 3

Figure S6. ^{13}C NMR of Limonin (3).. 3

Figure S7. ^{1}H NMR of Obacunone 17-*O*-*β*-D-glucopyranoside (4).. 4

Figure S8. ^{13}C NMR of Obacunone 17-*O*-*β*-D-glucopyranoside (4).. 4

Figure S9. ^{1}H NMR of Diosmetin (5)..5

Figure S10. ^{13}C NMR of Diosmetin (5).. 5

Figure S11. ^{1}H NMR of Luteolin (6).. 6

Figure S12. ^{13}C NMR of Luteolin (6).. 6

Figure S13. ^{1}H NMR of Isoquercetin (7)..7

Figure S14. ^{13}C NMR of Isoquercetin (7)..7

Figure S15. ^{1}H NMR of Nobiletin (8)..8

Figure S16. ^{13}C NMR of Nobiletin (8)..8

Figure S17. ^{1}H NMR of Isosakuranetin (9).. 9

Figure S18. ^{13}C NMR of Isosakuranetin (9).. 9

Figure S19. ^{1}H NMR of Epicatechin (10)..10

Figure S20. ^{13}C NMR of Epicatechin (10)..10

Figure S21. ^{1}H NMR of Kaempferol (11)..11

Figure S22. ^{13}C NMR of Kaempferol (11)..11

Figure S23. ^{1}H NMR of Kaempferol-3-*O*-*β*-*D*-glucopyranoside (12)....................................12

Figure S24. ^{13}C NMR of Kaempferol-3-*O*-*β*-*D*-glucopyranoside (12)....................................12

Figure S25. ^{1}H NMR of Kaempferol-3-*O*-(6''-*O*-acetyl) -*β*-*D*-glucopyranoside (13).......... 13

Figure S26. ^{13}C NMR of Kaempferol-3-*O*-(6''-*O*-acetyl) -*β*-*D*-glucopyranoside (13)......... 13

Figure S27. ^{1}H NMR of Kaempferol-3-*O*-glucosyl-6''-*O*-pentadionic acid (14)..................14

Figure S28. ^{13}C NMR of Kaempferol-3-*O*-glucosyl-6''-*O*-pentadionic acid (14)................ 14

Figure S29. ^{1}H NMR of 5, 7, 4′-trihydroxy-8, 3′-dimethoxyflavone-3-*O*-6″-3-hydroxyl-3-m -ethylglutaroyl)-*β*-*D*-glucopyranoside (15)..15

Figure S30. ^{13}C NMR NMR of 5, 7, 4′-trihydroxy-8, 3′-dimethoxyflavone-3-*O*-6″-3-hydro -xyl-3-methylglutaroyl)-*β*-*D*-glucopyranoside (15)...15

Figure S31. ^{1}H NMR of 5, 7, 4′-trihydroxy-6, 8, 3′-dimethoxyflavone-3-*O*-6″-3-hydroxyl-3 -methylglutaroyl)-*β*-*D*-glucopyranoside (16)..16

Figure S32. ^{13}C NMR NMR of 5, 7, 4′-trihydroxy-6, 8, 3′-dimethoxyflavone-3-*O*-6″-3-hyd -roxyl-3methylglutaroyl)-*β*-*D*-glucopyranoside (16)..16

Figure S33. ^{1}H NMR of Hesperitin (17)...17

Figure S34. ^{13}C NMR of Hesperitin (17)...17

Figure S35. ^{1}H NMR of Hesperidin (18)...18

Figure S36. ^{13}C NMR of Hesperidin (18)..18
Figure S37. ^{1}H NMR of Neohesperidin (19)...19
Figure S38. ^{13}C NMR of Neohesperidin (19)..19
Figure S39. ^{1}H NMR of Eriodictioside (20)..20
Figure S40. ^{13}C NMR of Eriodictioside (20)...20
Figure S41. ^{1}H NMR of Phlorizin (21)..21
Figure S42. ^{13}C NMR of Phlorizin (21)..21
Figure S43. ^{1}H NMR of 8-Hydroxypinoresinol-4'-*O*-*β*-*D*-Glucopyranoside (22)................22
Figure S44. ^{13}C NMR of 8-Hydroxypinoresinol-4'-*O*-*β*-*D*-Glucopyranoside (22)..............22
Figure S45. ^{1}H NMR of Pumilaside A (23)...23
Figure S46. ^{13}C NMR of Pumilaside A (23)... 23
Figure S47. ^{1}H NMR of 4-hydroxy-2-methoxyphenol-1-*O*-*β*-*D*-glucopyranoside (24).......24
Figure S48. ^{13}C NMR of 4-hydroxy-2-methoxyphenol-1-*O*-*β*-*D*-glucopyranoside (24)......24
Figure S49. ^{1}H NMR of Phenylethyl-rutinoside (25)..25
Figure S50. ^{13}C NMR of Phenylethyl-rutinoside (25)...25
Figure S51. ^{1}H NMR of Trans-*p*-menthane-1*α*,2*β*,8-triol (26).. 26
Figure S52. ^{13}C NMR of Trans-*p*-menthane-1*α*,2*β*,8-triol (26)... 26
Table 1. KEGG enrichment results..33
Table 2. The Vina Scores for docking...33

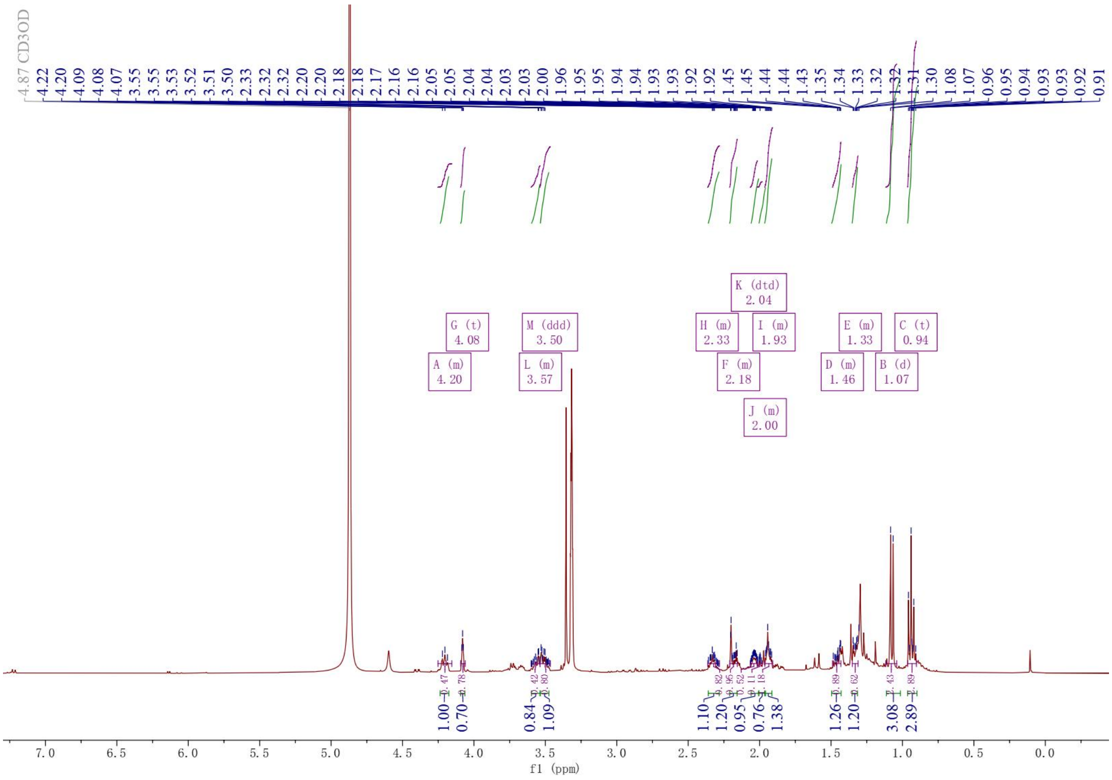

Figure S1. ^{1}H **NMR (Bruker AV-400, 400 MHz,** CD_3OD**) of Callyspongidipeptide A (1)**

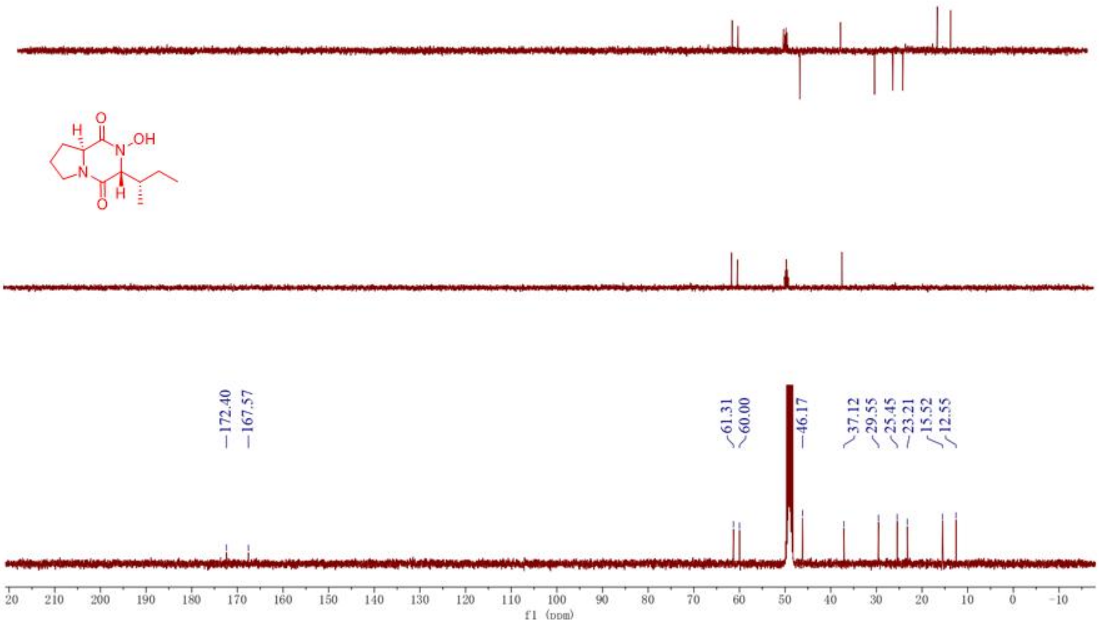

Figure S2. ^{13}C **NMR (Bruker AV-400, 100 MHz,** CD_3OD**) of Callyspongidipeptide A (1)**

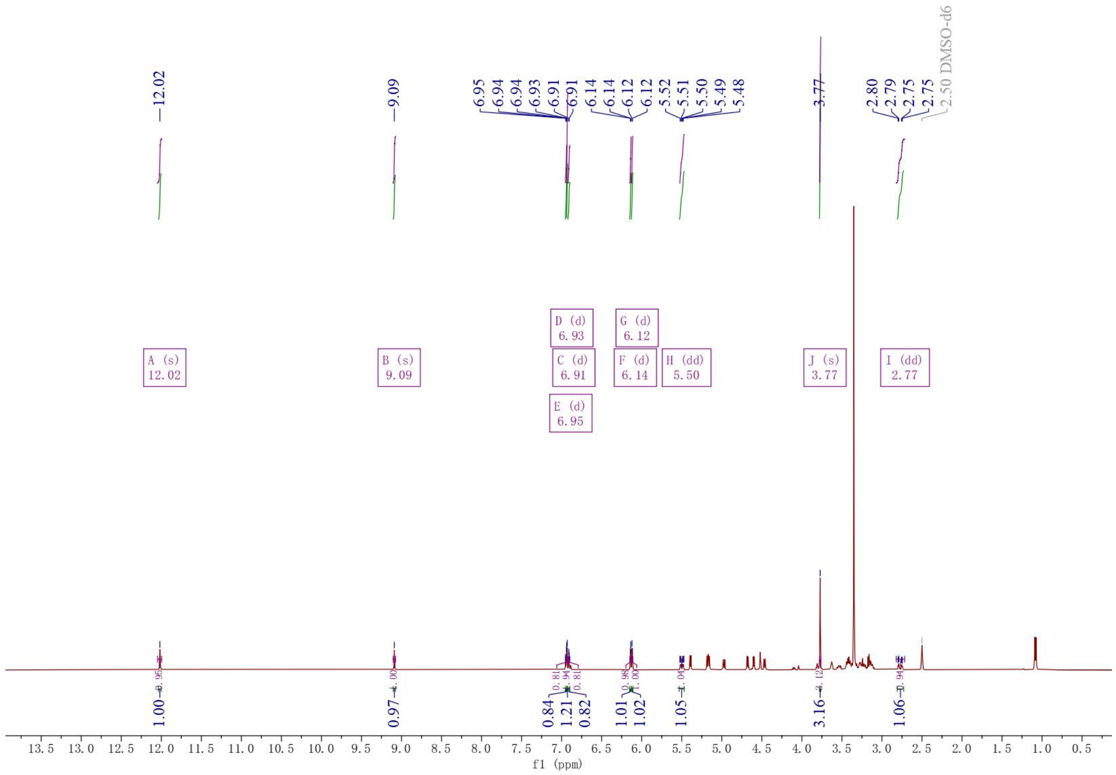

Figure S3. **^{1}H NMR (Bruker AV-400, 400 MHz, (DMSO-d6) of Hesperetin 7-*O*-*β*-*D*-glucopyranoside (2)**

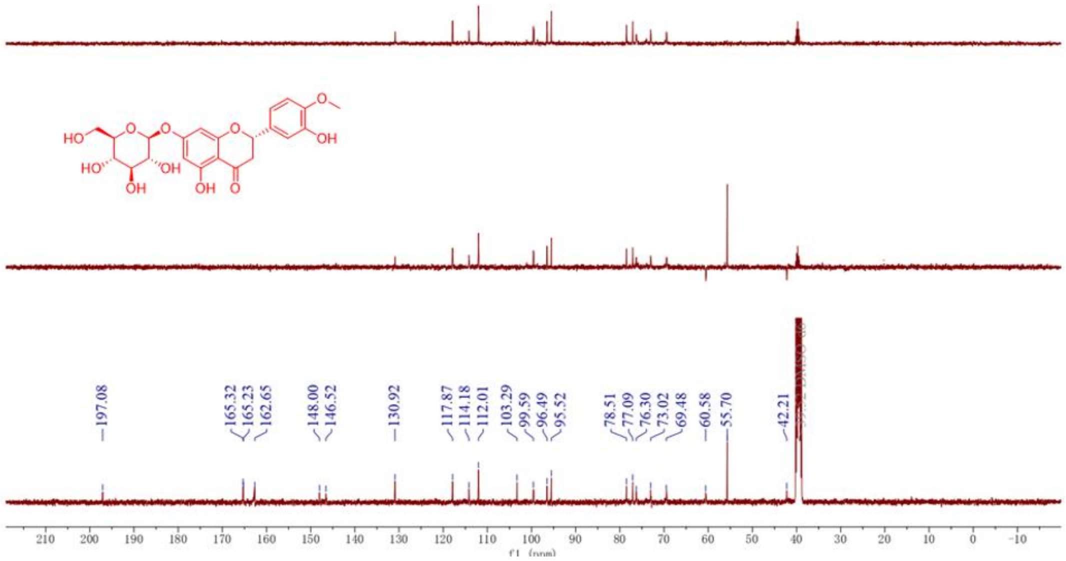

Figure S4. **^{13}C NMR (Bruker AV-400, 100 MHz, DMSO-d6) of Hesperetin 7-*O*-*β*-*D*-glucopyranoside (2)**

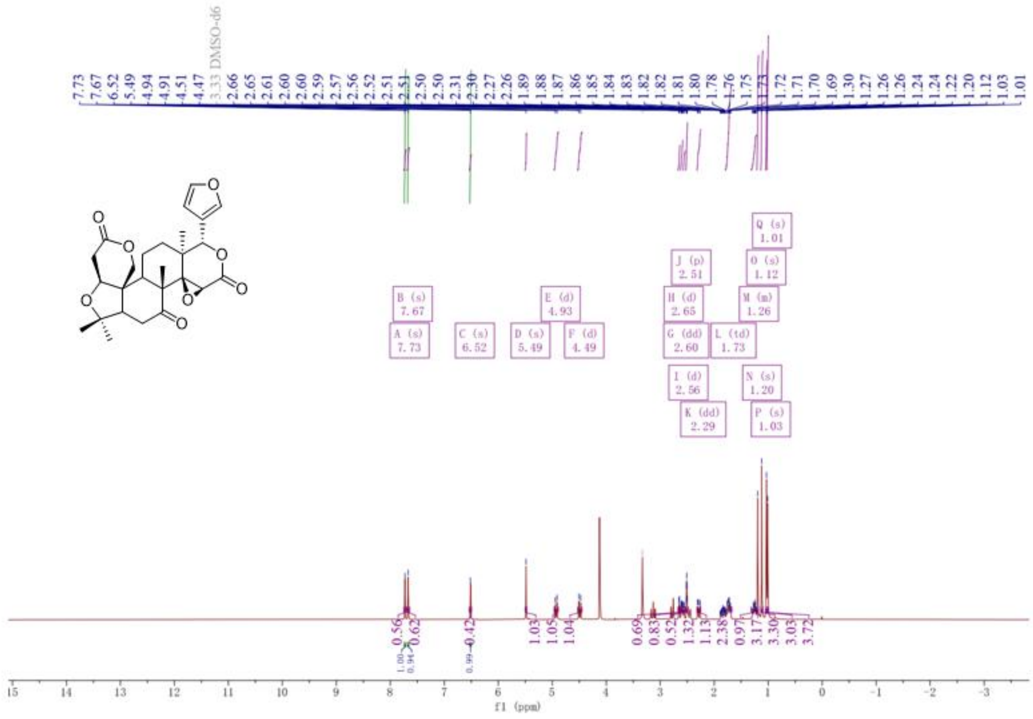

Figure S5. ^{1}H NMR (Bruker AV-400, 400 MHz, (DMSO-d6) of Limonin (3)

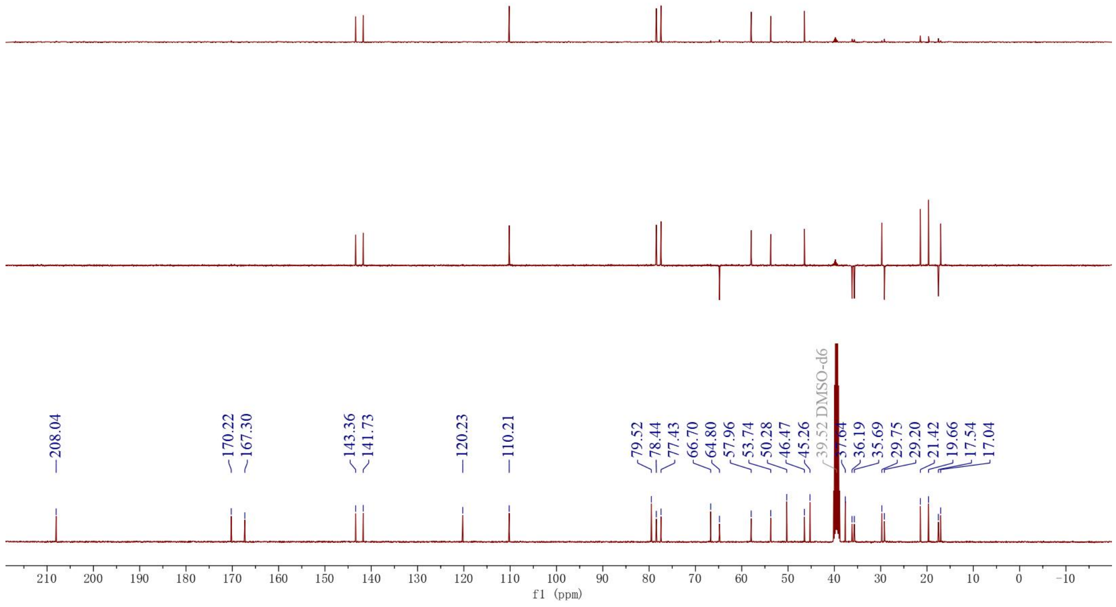

Figure S6. ^{13}C NMR (Bruker AV-400, 100 MHz, DMSO-d6) of Obacunone 17-*O*-*β*-D-glucopyranoside (3)

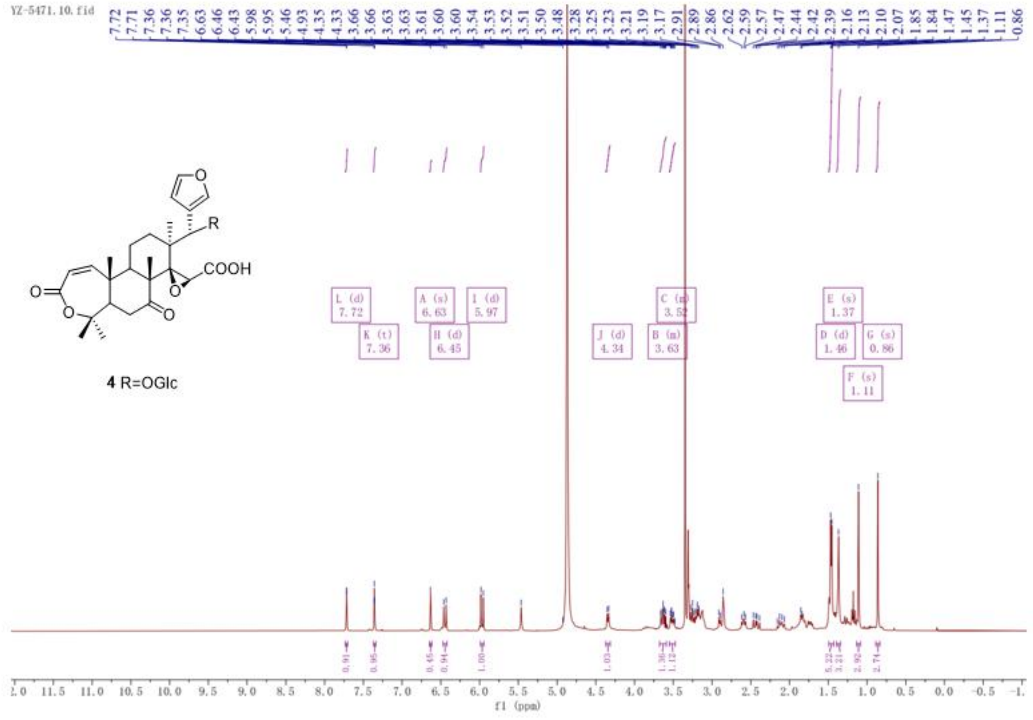

Figure S7. ^{1}H NMR (Bruker AV-400, 400 MHz, CD_3OD) of Callyspongidipeptide A (4)

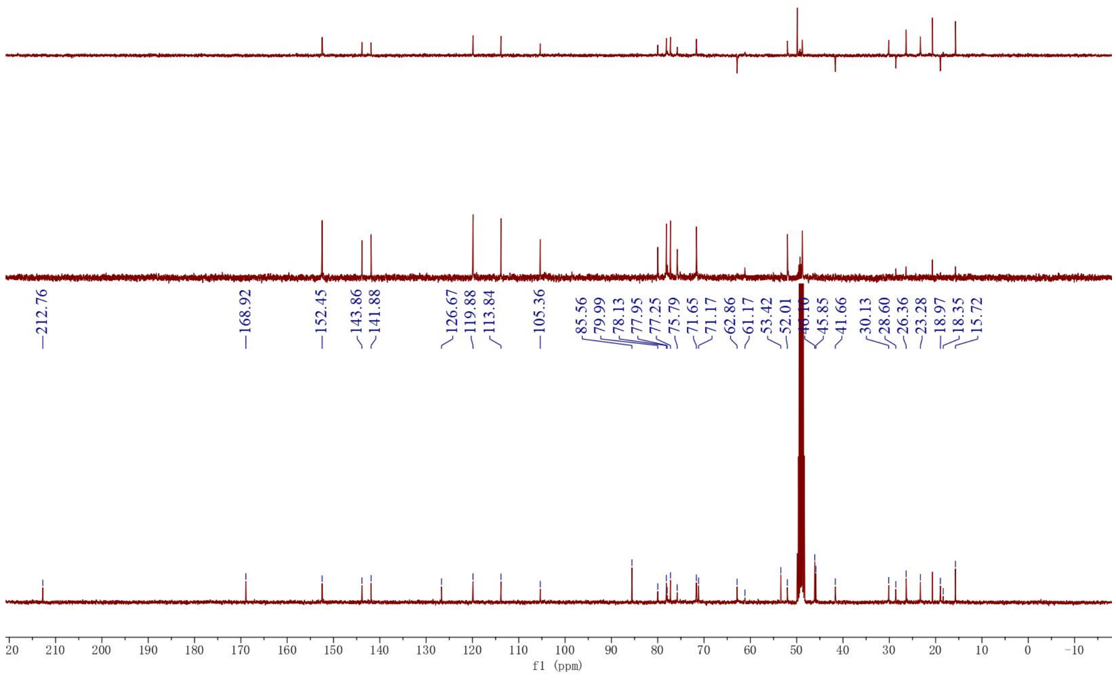

Figure S8. ^{13}C NMR (Bruker AV-400, 100 MHz, CD_3OD) of Obacunone 17-*O*-*β*-D-glucopyranoside (4)

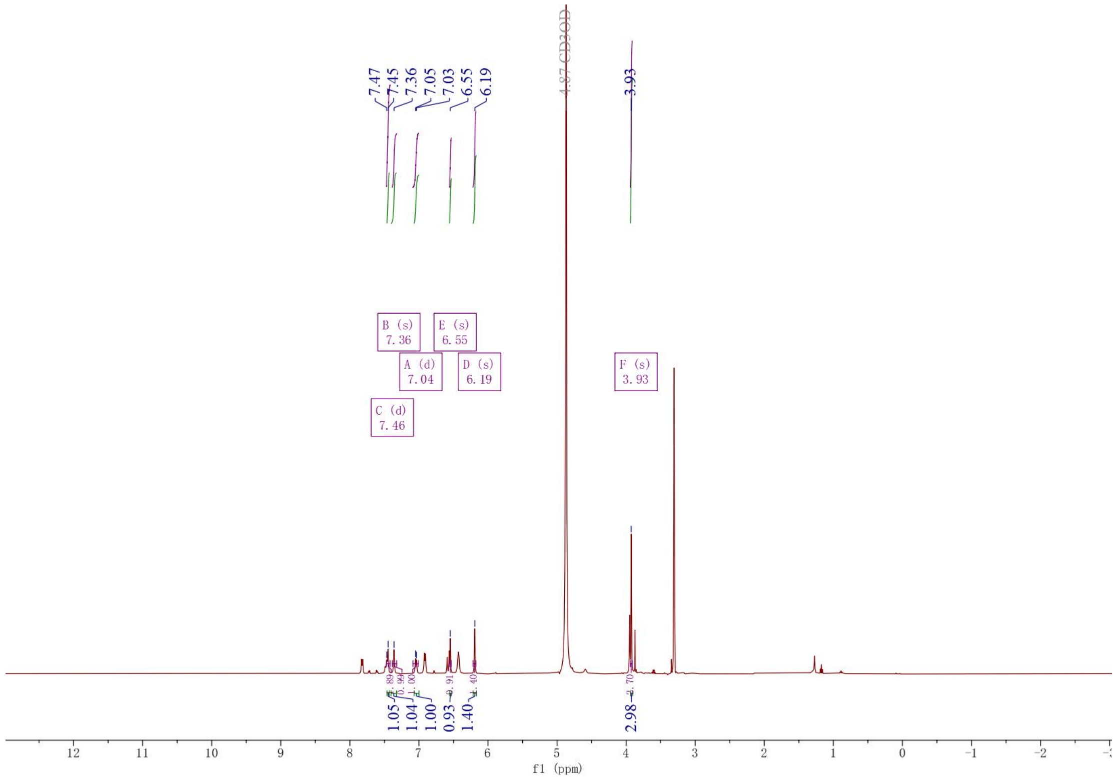

Figure S9. ¹H NMR (Bruker AV-400, 400 MHz, CD_3OD) of Diosmetin (5)

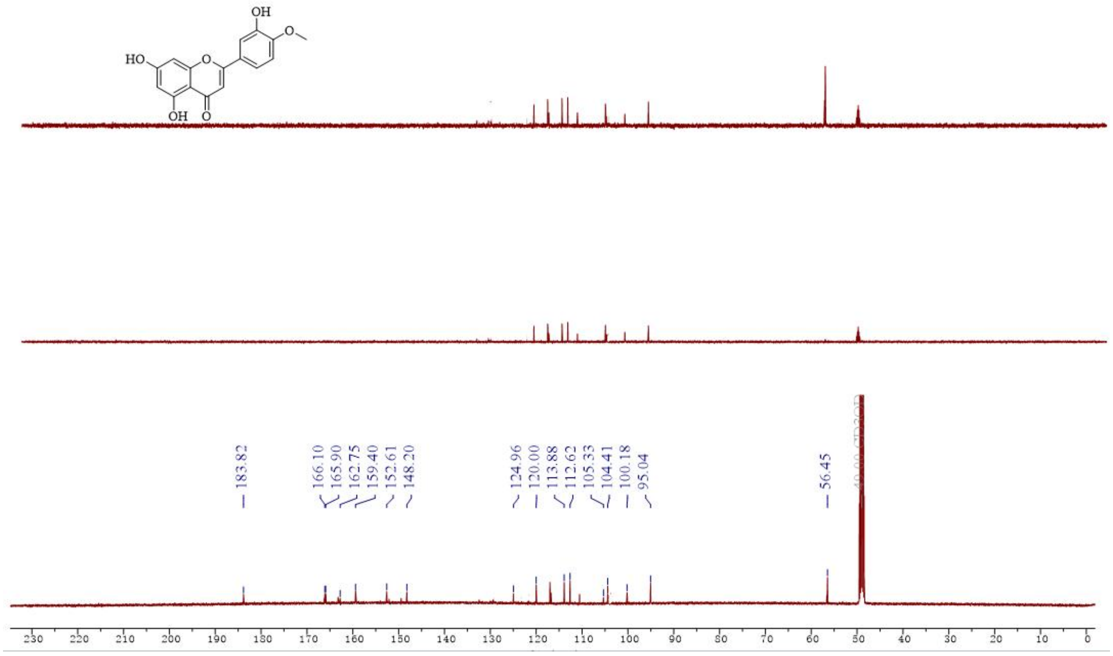

Figure S10. ¹³C NMR (Bruker AV-400, 100 MHz, CD_3OD) of Diosmetin (5)

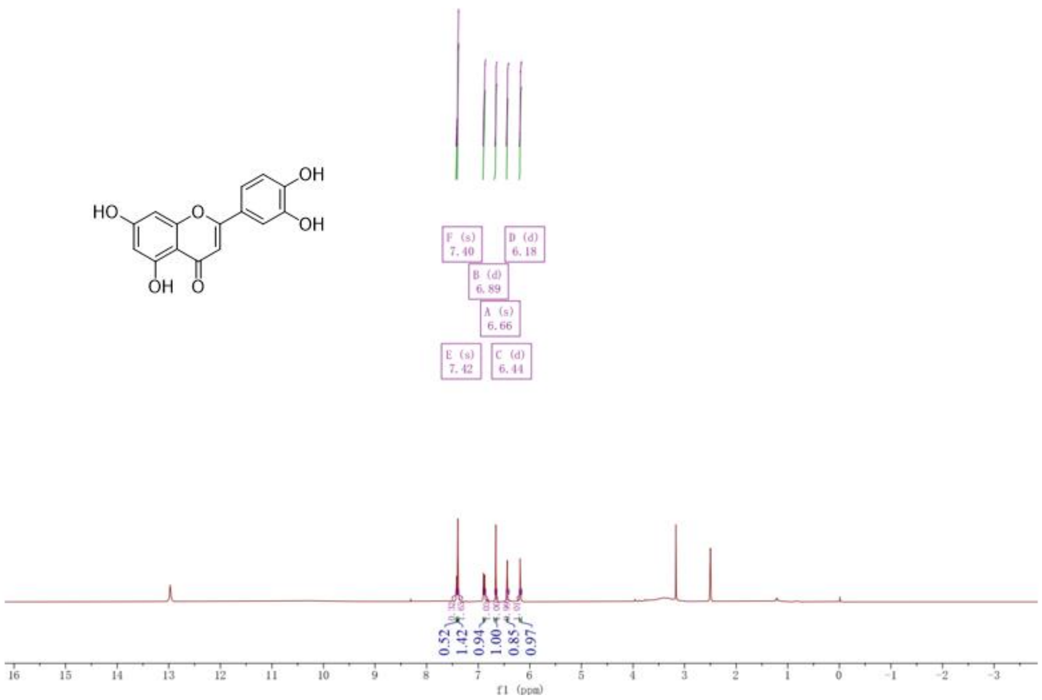

Figure S11. ^{1}H NMR (Bruker AV-400, 400 MHz, CD_3OD) of Luteolin (6)

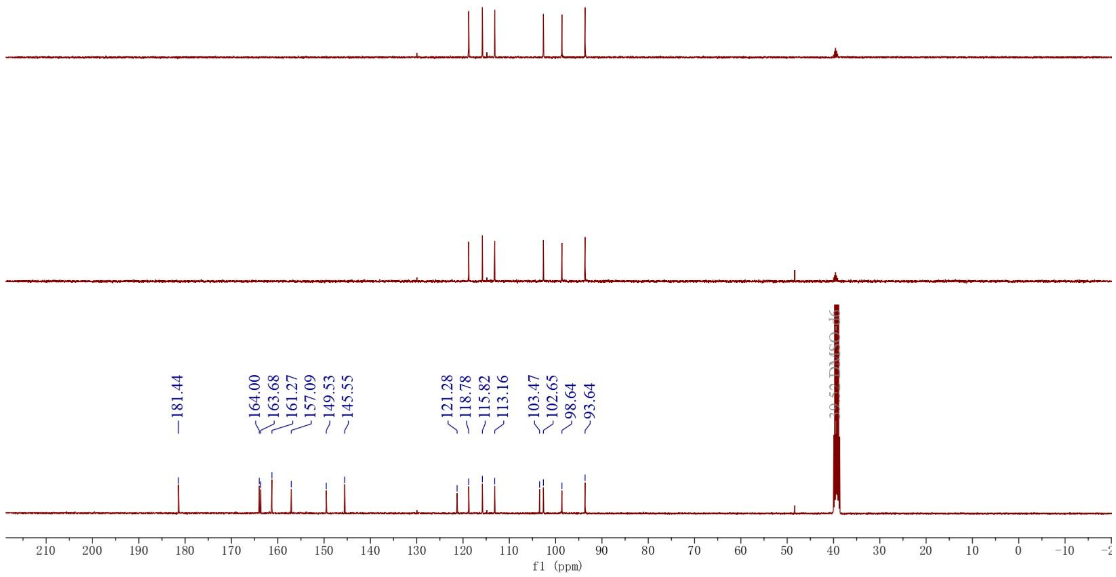

Figure S12. ^{13}C NMR (Bruker AV-400, 100 MHz, CD_3OD) of Luteolin (6)

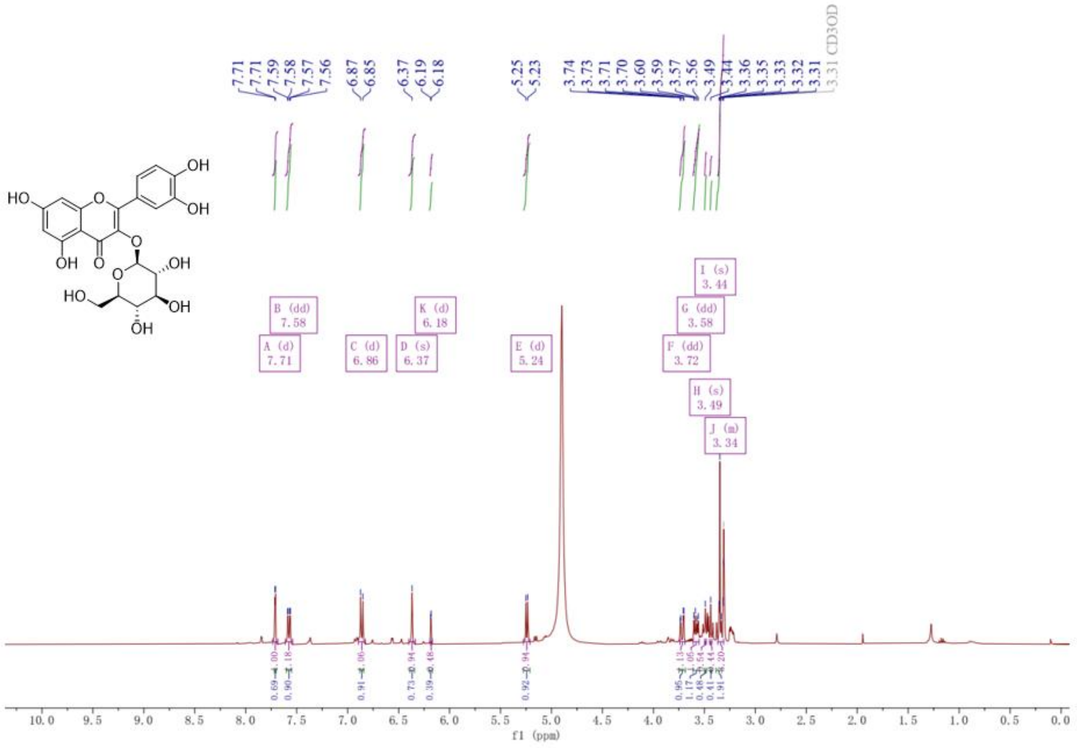

Figure S13. ^{1}H NMR (Bruker AV-400, 400 MHz, CD_3OD) of Isoquercetin (7)

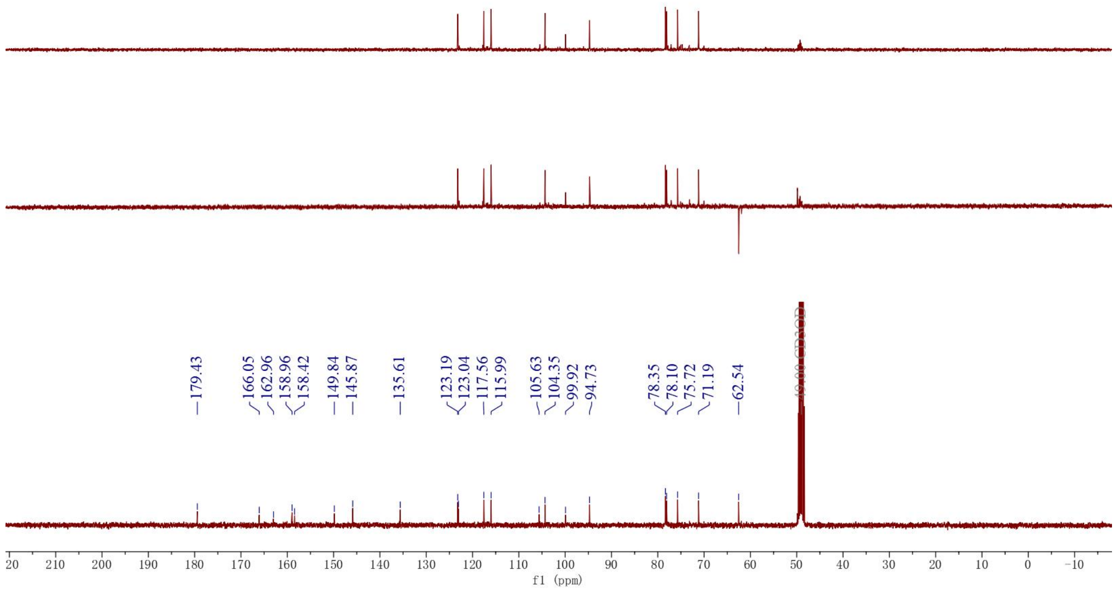

Figure S14. ^{13}C NMR (Bruker AV-400, 100 MHz, CD_3OD) of Isoquercetin (7)

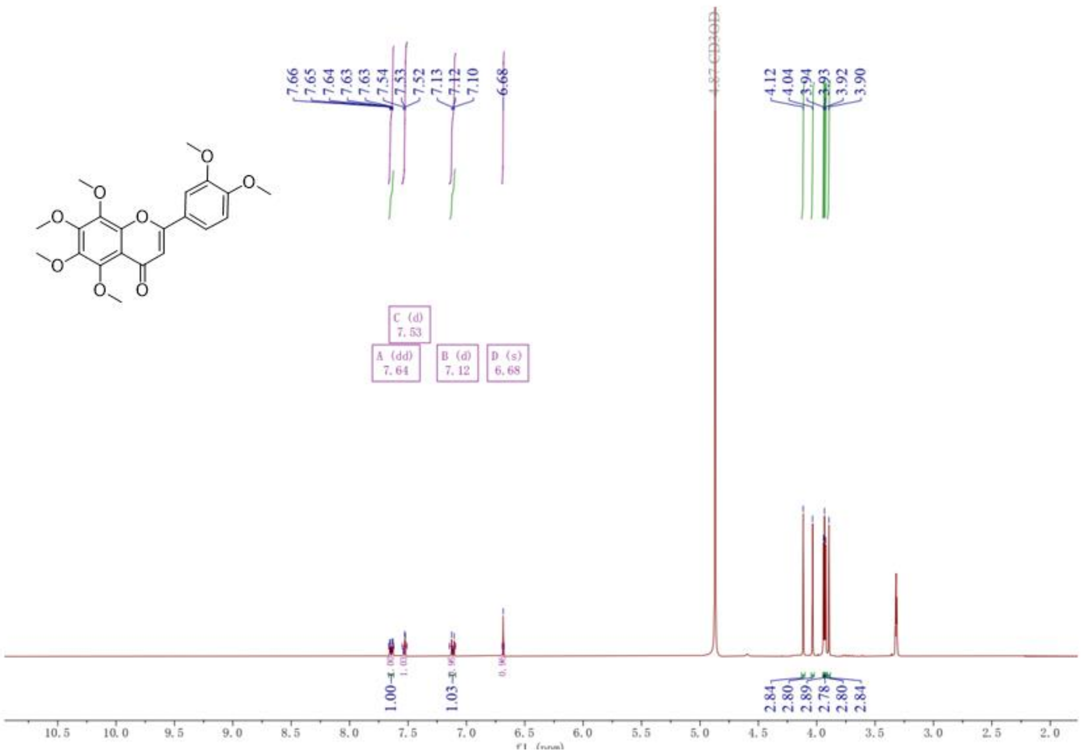

Figure S15. ¹H NMR (Bruker AV-400, 400 MHz, CD_3OD) of Nobiletin (8)

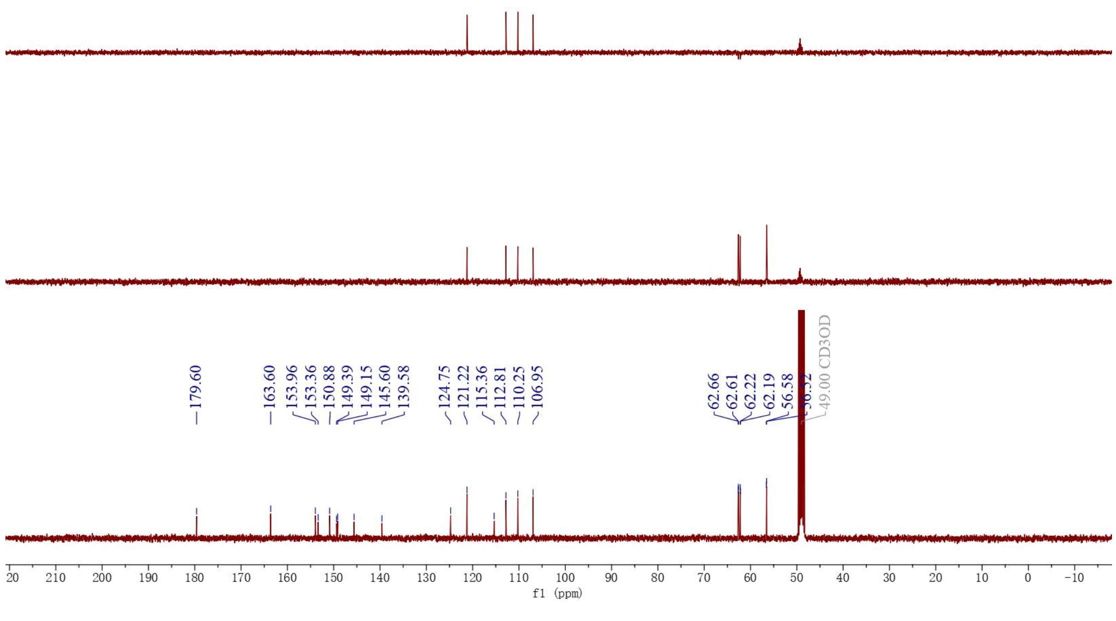

Figure S16. ¹³C NMR (Bruker AV-400, 100 MHz, CD_3OD) of Nobiletin (8)

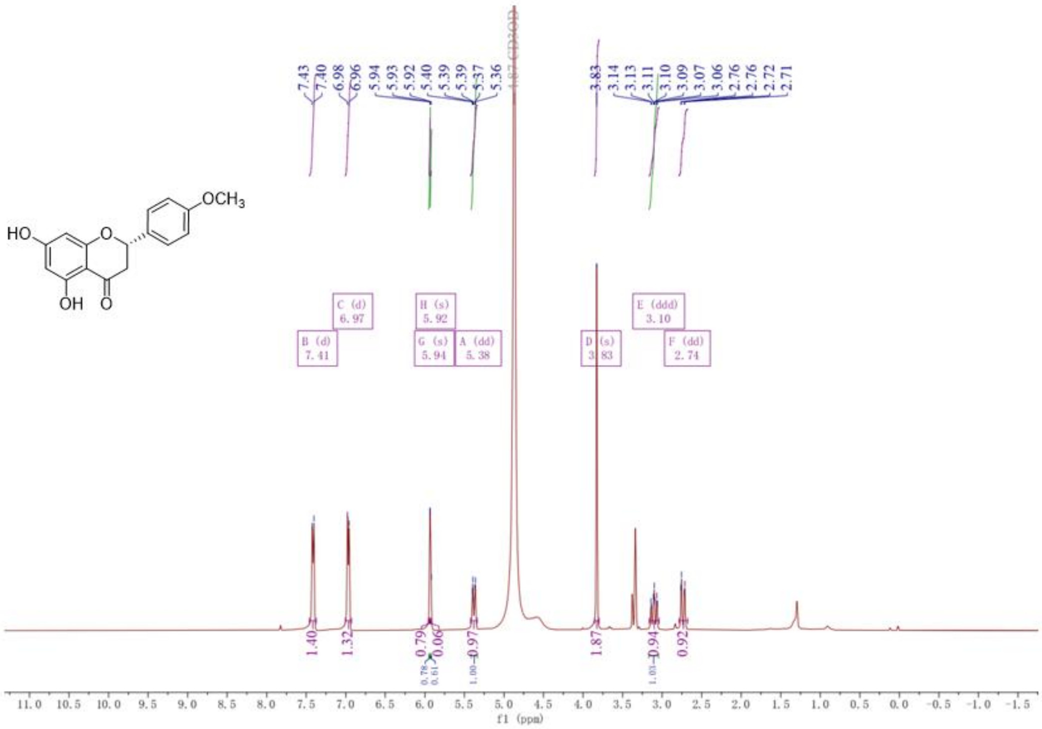

Figure S17. ^{1}H NMR (Bruker AV-400, 400 MHz, CD3OD) of Isosakuranetin (9)

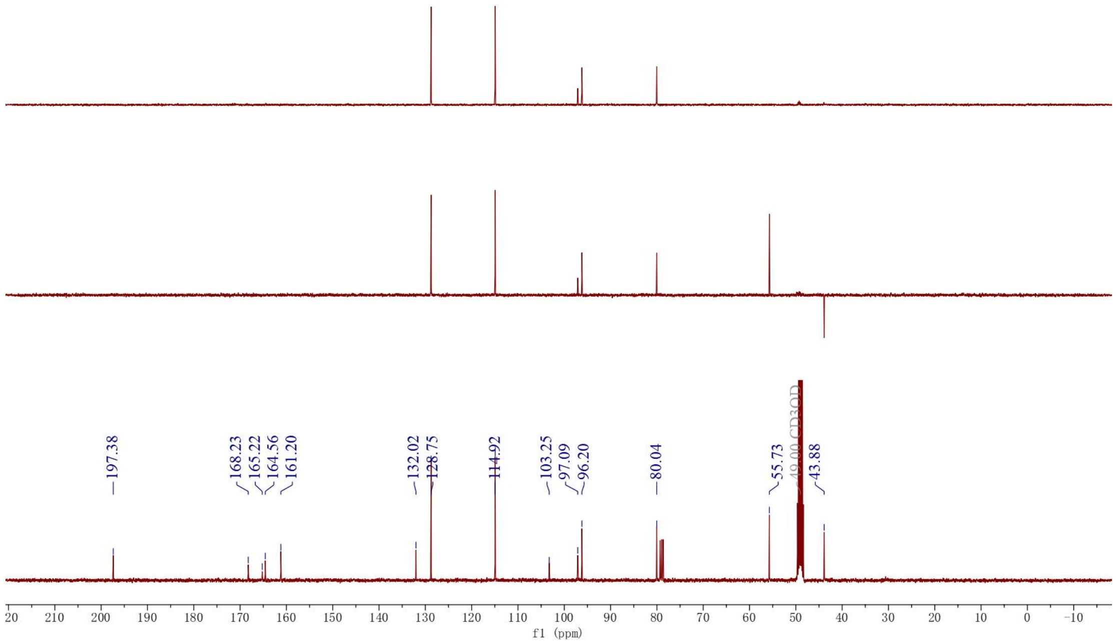

Figure S18. ^{13}C NMR (Bruker AV-400, 100 MHz, CD3OD) of Isosakuranetinn (9)

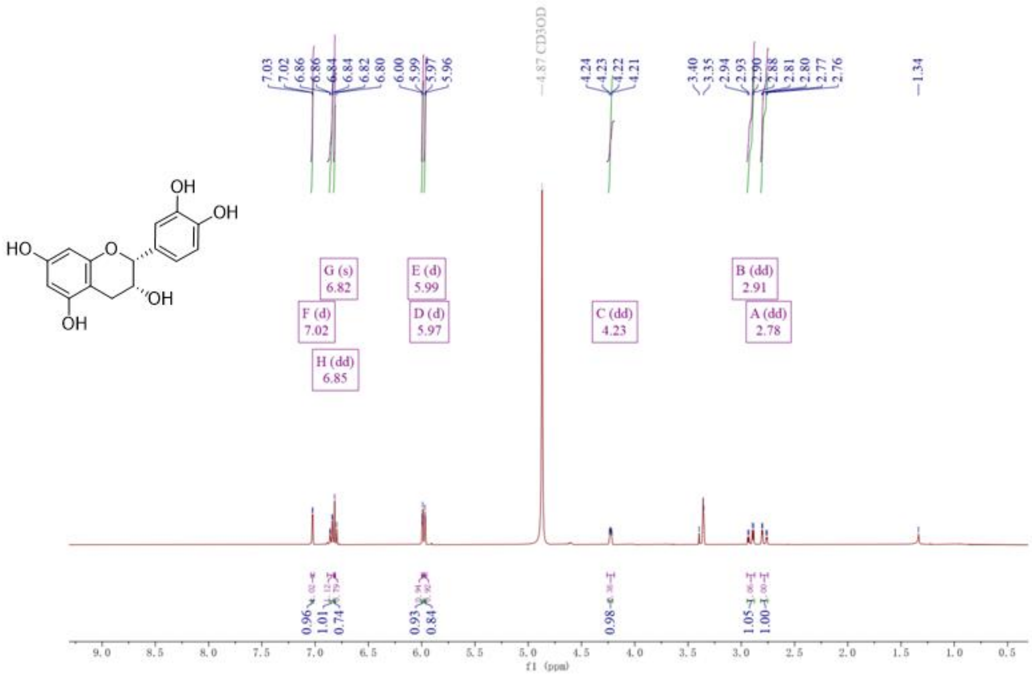

Figure S19. ^{1}**H NMR (Bruker AV-400, 400 MHz,** CD_3OD**) of Epicatechin (10)**

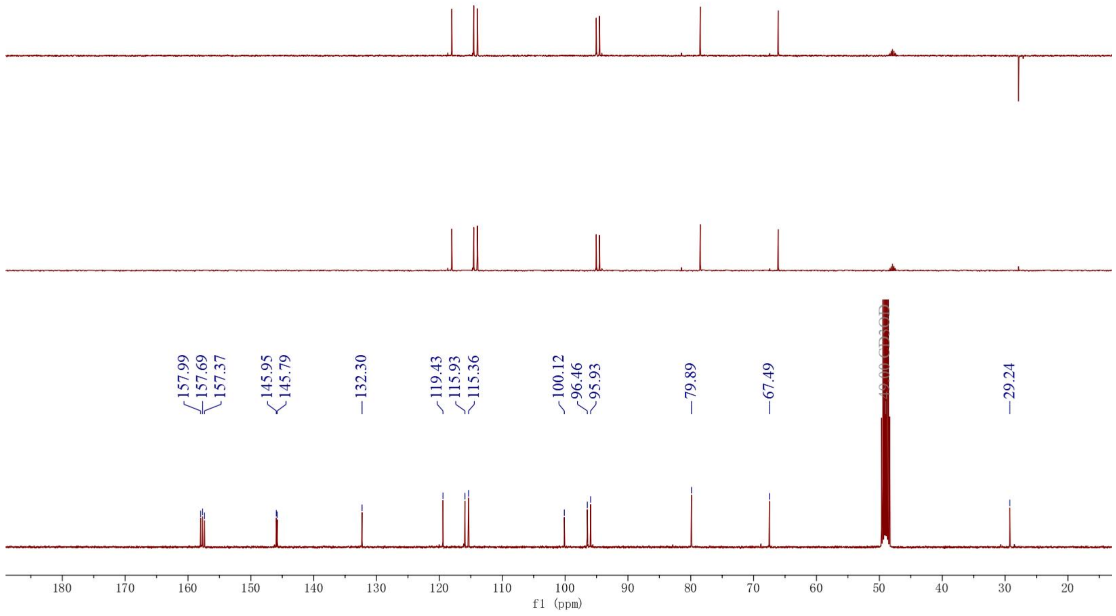

Figure S20. ^{13}**C NMR (Bruker AV-400, 100 MHz,** CD_3OD**) of Epicatechin (10)**

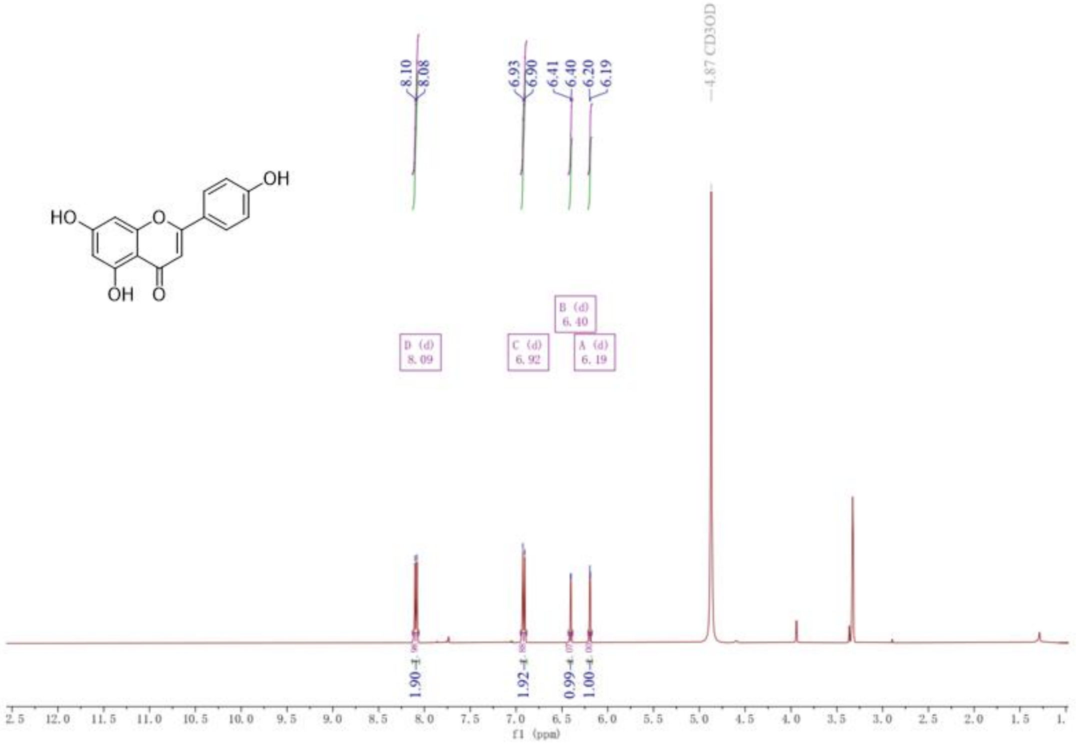

Figure S21. ^{1}H NMR (Bruker AV-400, 400 MHz, CD_3OD) of Kaempferol (11)

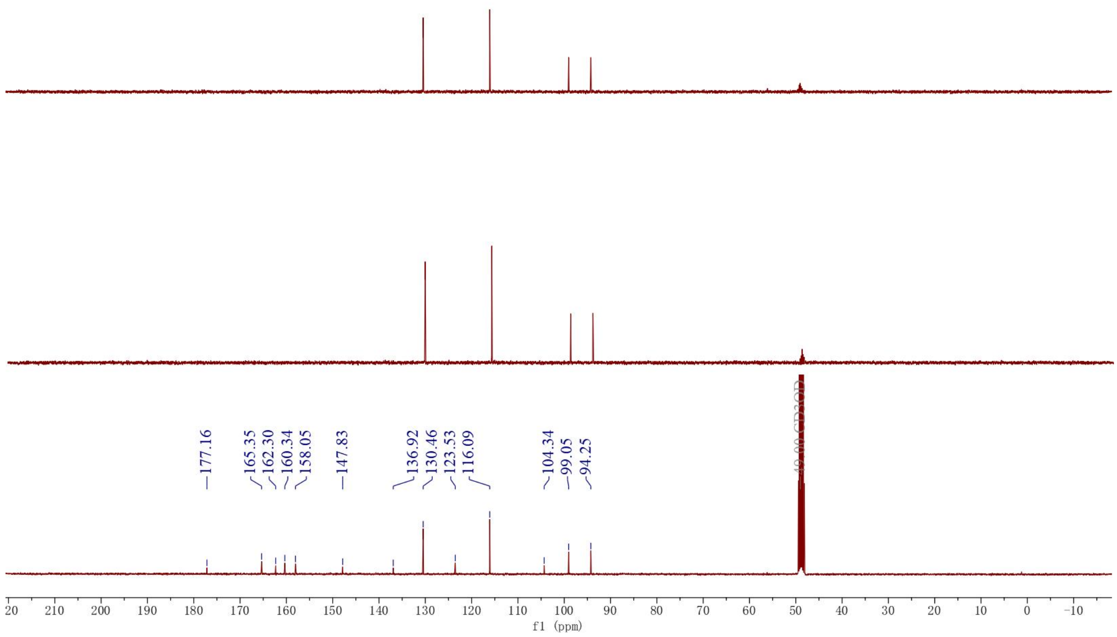

Figure S22. ^{13}C NMR (Bruker AV-400, 100 MHz, CD_3OD) of Kaempferol (11)

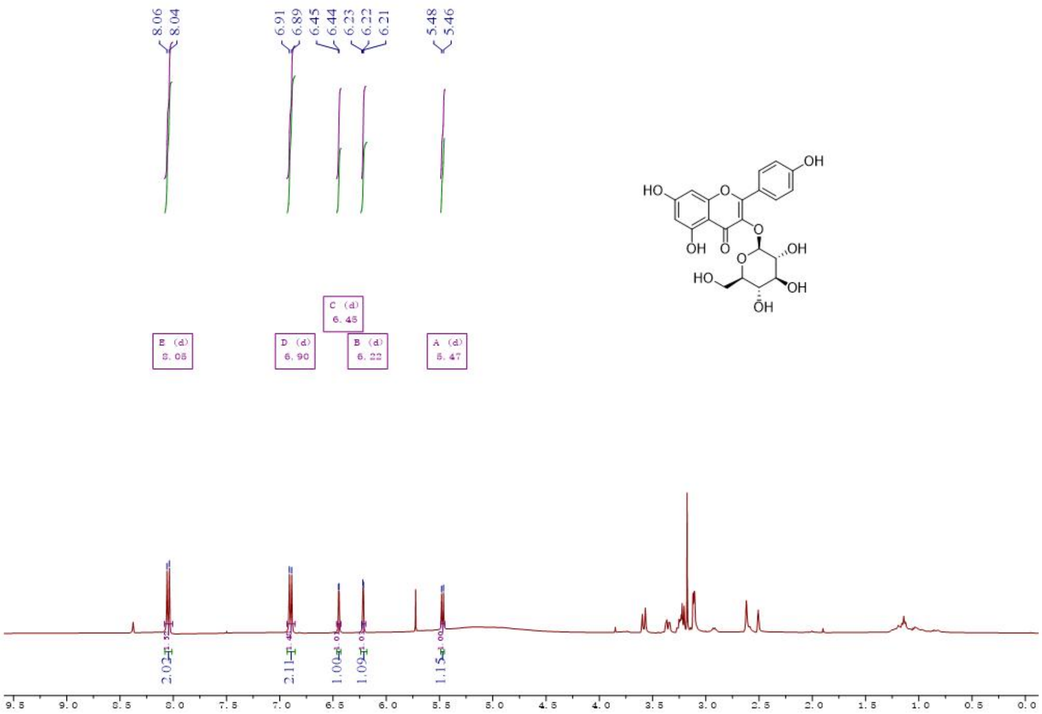

Figure S23. **^{1}H NMR (Bruker AV-400, 400 MHz, CD_3OD) of Kaempferol-3-*O*-*β*-*D*-glucopyranoside (12)**

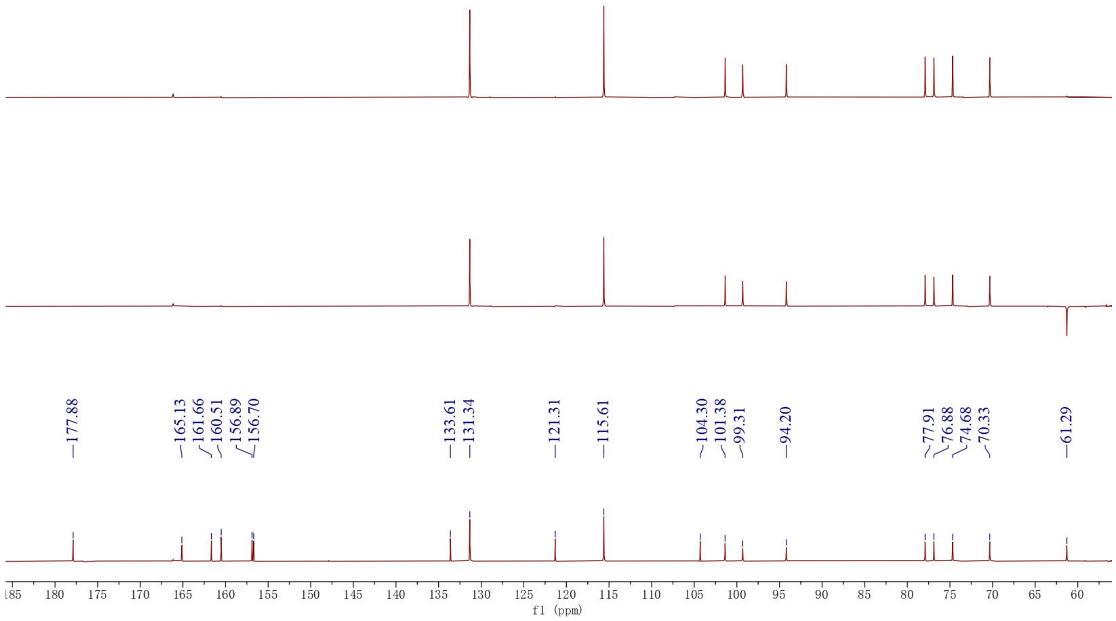

Figure S24. **^{13}C NMR (Bruker AV-400, 100 MHz, CD_3OD) of Kaempferol-3-*O*-*β*-*D*-glucopyranoside (12)**

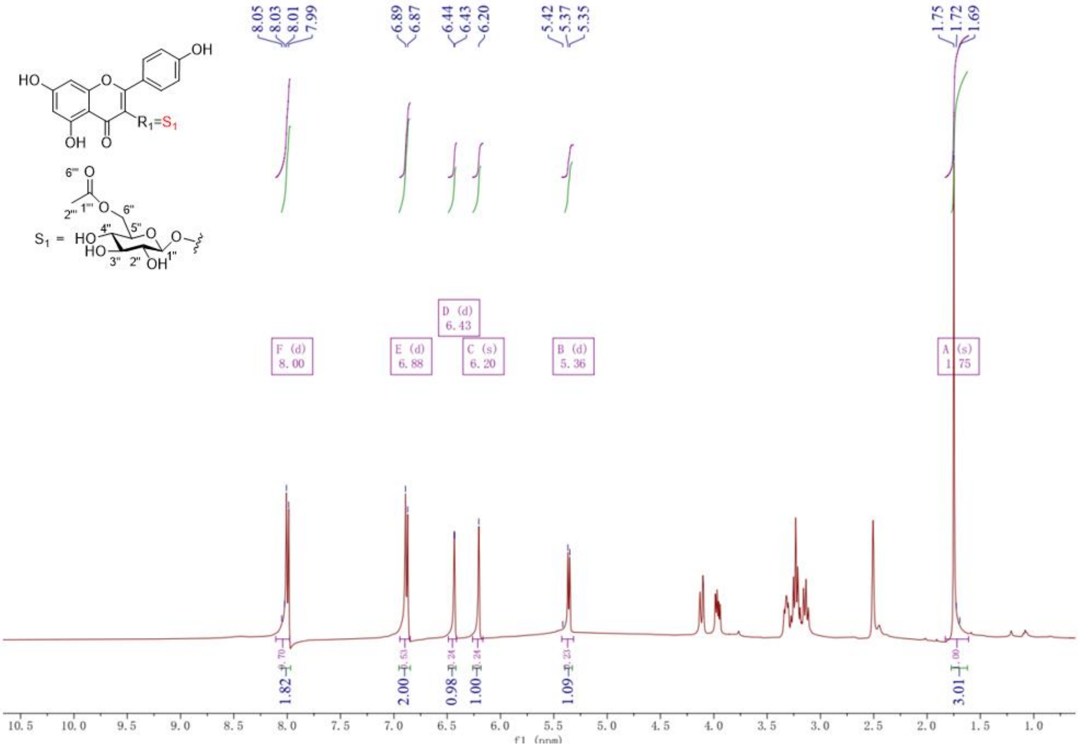

Figure S25. ^{1}H NMR (Bruker AV-400, 400 MHz, CD_3OD) of Kaempferol-3-*O*-(6''-*O*-acetyl) -*β*-*D*-glucopyranoside (13)

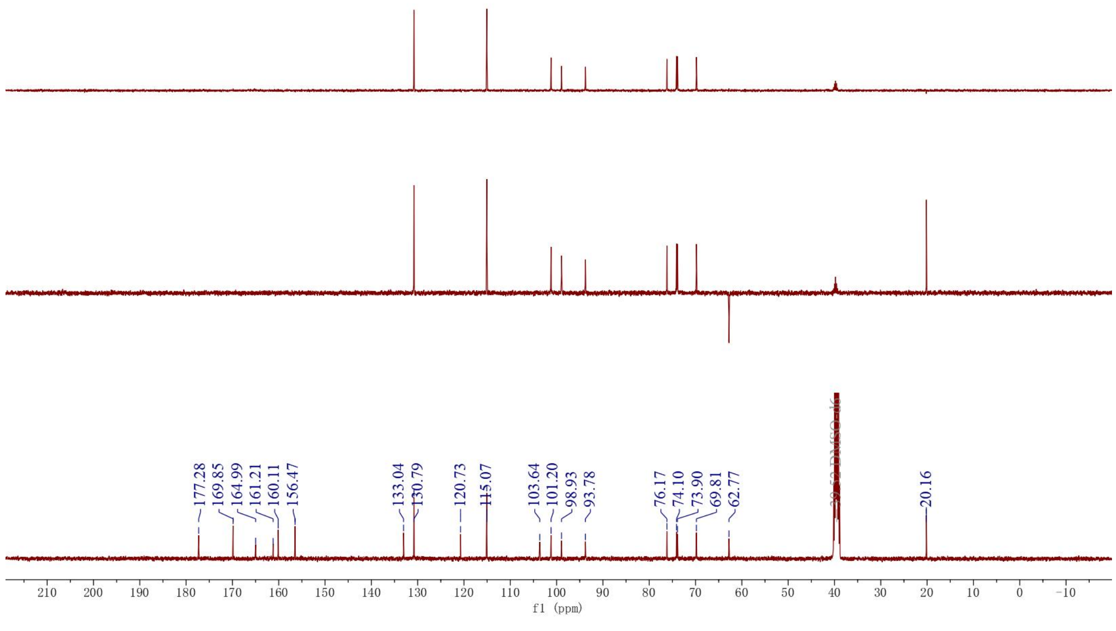

Figure S26. ^{13}C NMR (Bruker AV-400, 100 MHz, CD_3OD) of Kaempferol-3-*O*-(6''-*O*-acetyl) -*β*-*D*-glucopyranoside (13)

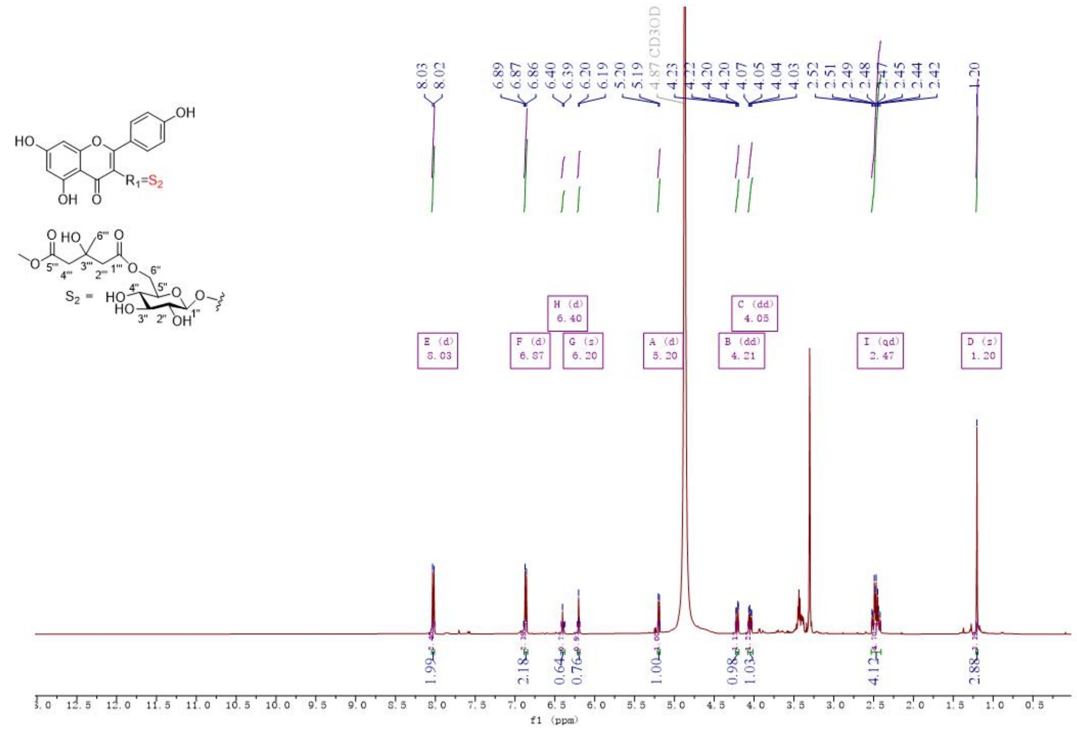

Figure S27. **^{1}H NMR (Bruker AV-400, 400 MHz, CD_3OD) of Kaempferol-3-*O*-glucosyl-6''-*O*-pentadionic acid (14)**

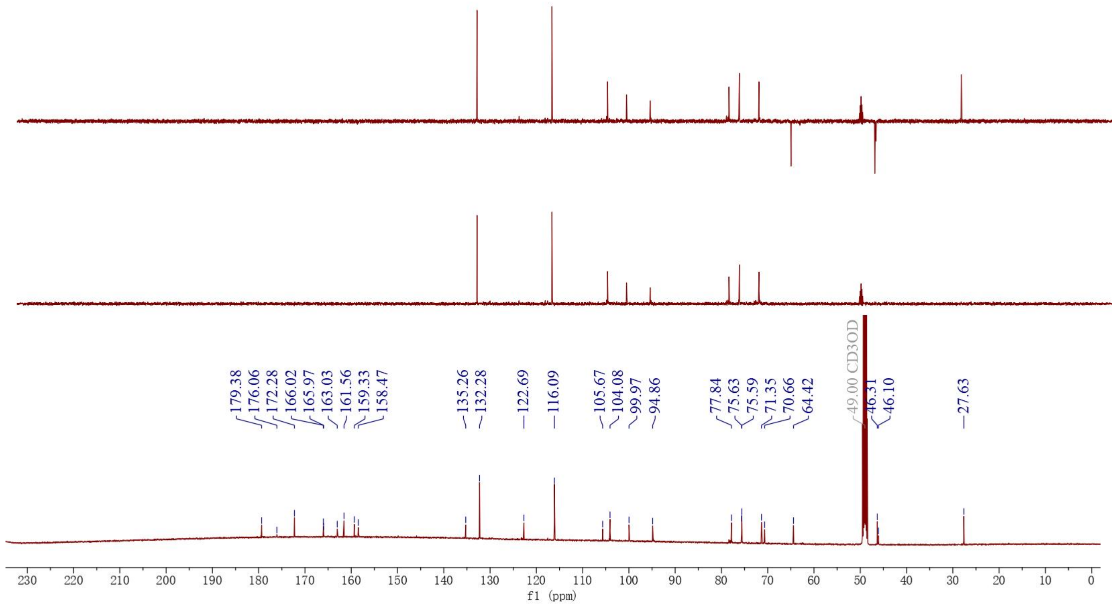

Figure S28. **^{13}C NMR (Bruker AV-400, 100 MHz, CD_3OD) of Kaempferol-3-*O*-glucosyl-6''-*O*-pentadionic acid (14)**

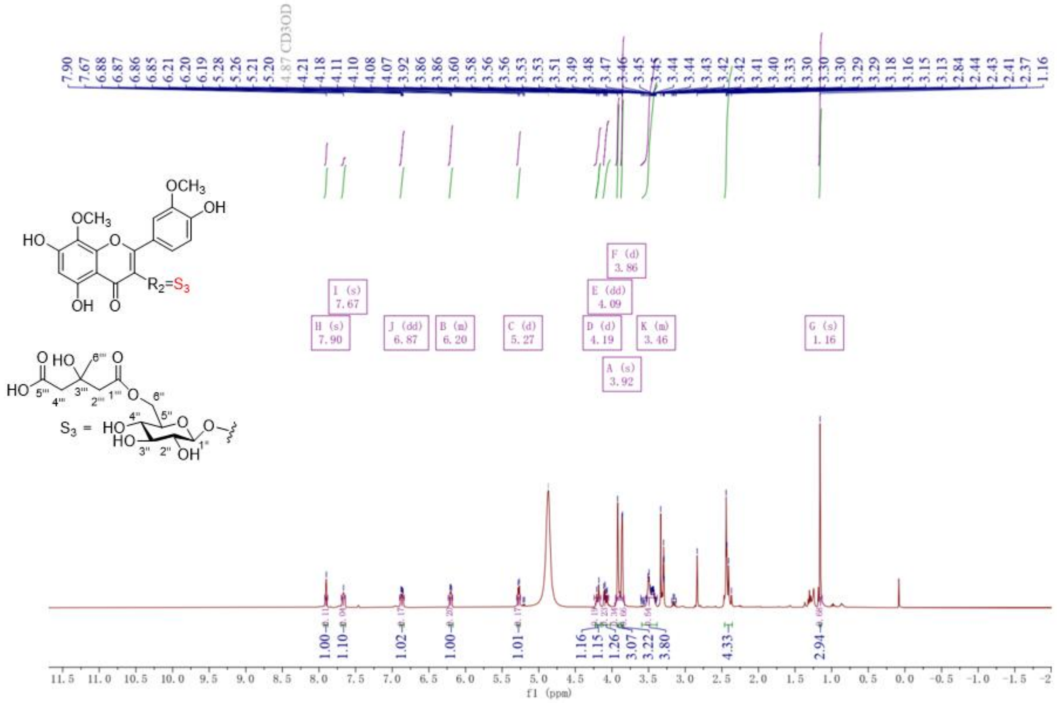

Figure S29. ^{1}H NMR (Bruker AV-400, 400 MHz, CD_3OD) of 5, 7, 4′-trihydroxy-8, 3′-dimethoxyflavone-3-*O*-6″-3-hydroxyl-3-methylglutaroyl)-*β*-*D*-glucopyranoside (15)

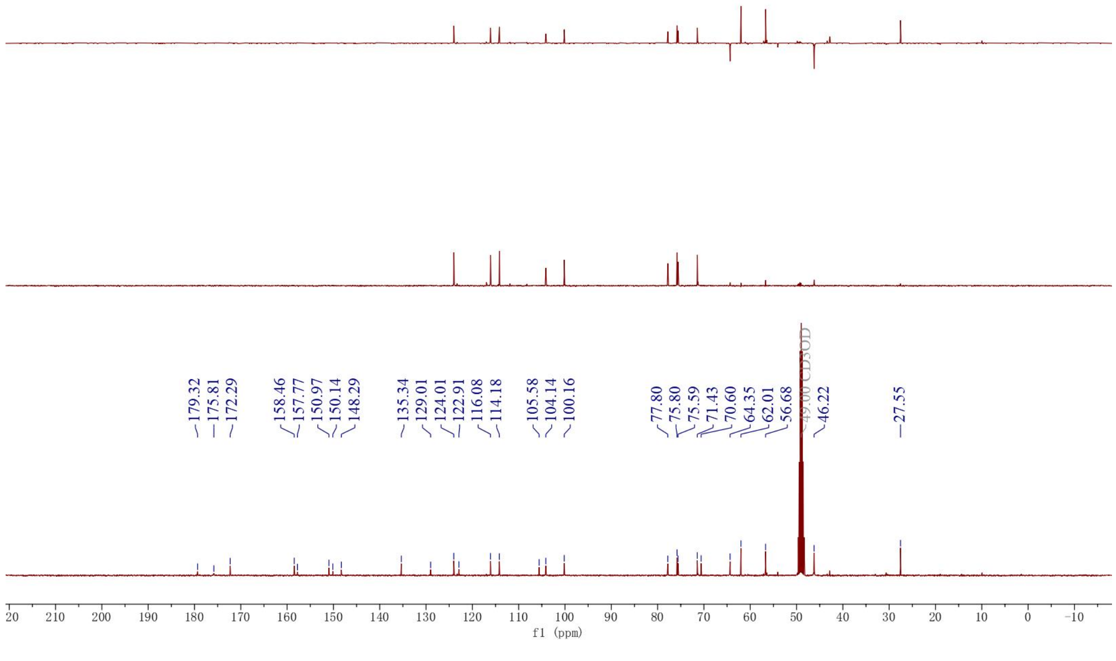

Figure S30. ^{13}C NMR (Bruker AV-400, 100 MHz, CD_3OD) of 5, 7, 4′-trihydroxy-8, 3′-dimethoxyflavone-3-*O*-6″-3-hydroxyl-3-methylglutaroyl)-*β*-*D*-glucopyranoside (15)

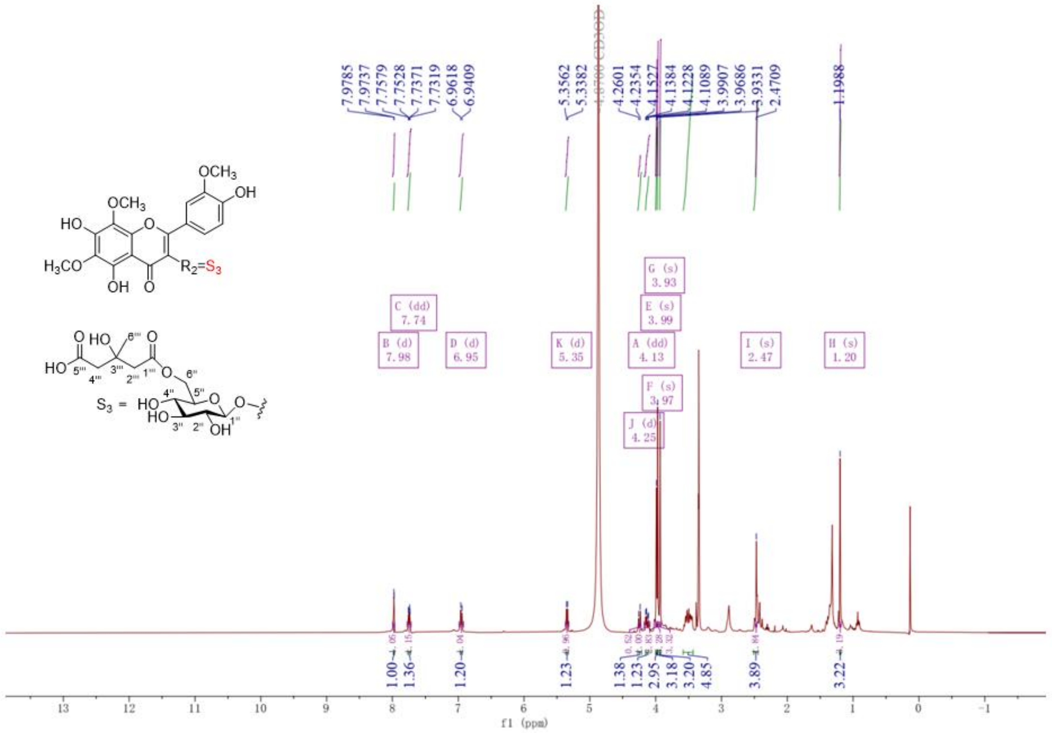

Figure S31. **^{1}H NMR (Bruker AV-400, 400 MHz, CD_3OD) of 5, 7, 4′-trihydroxy-6, 8, 3′-dimethoxyflavone-3-*O*-6″-3-hydroxyl-3-methylglutaroyl)-*β*-*D*-glucopyranoside (16)**

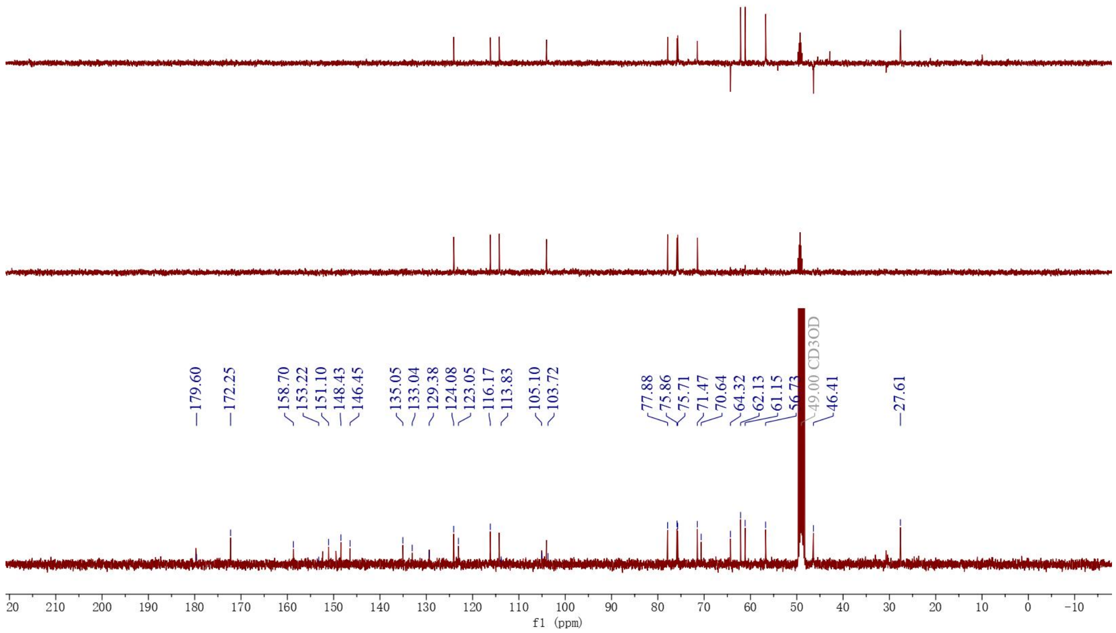

Figure S32. **^{13}C NMR (Bruker AV-400, 100 MHz, CD_3OD) of 5, 7, 4′-trihydroxy-6, 8, 3′-dimethoxyflavone-3-*O*-6″-3-hydroxyl-3-methylglutaroyl)-*β*-*D*-glucopyranoside (15)**

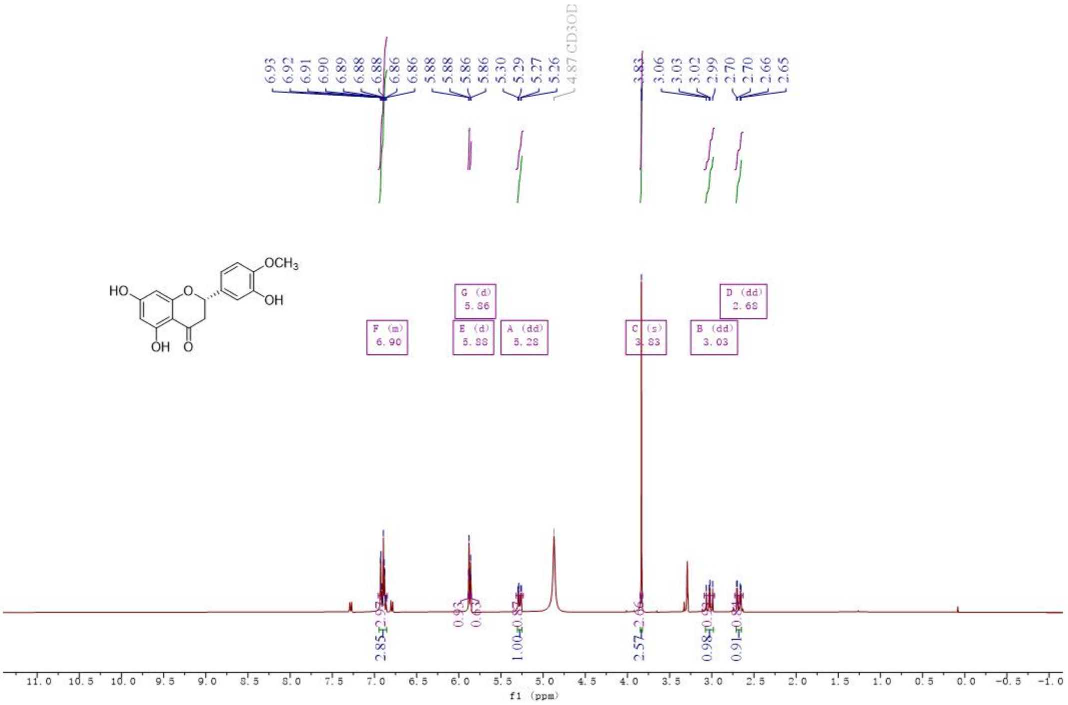

Figure S33. ^{1}H NMR (Bruker AV-400, 400 MHz, CD_3OD) of Hesperitin (17)

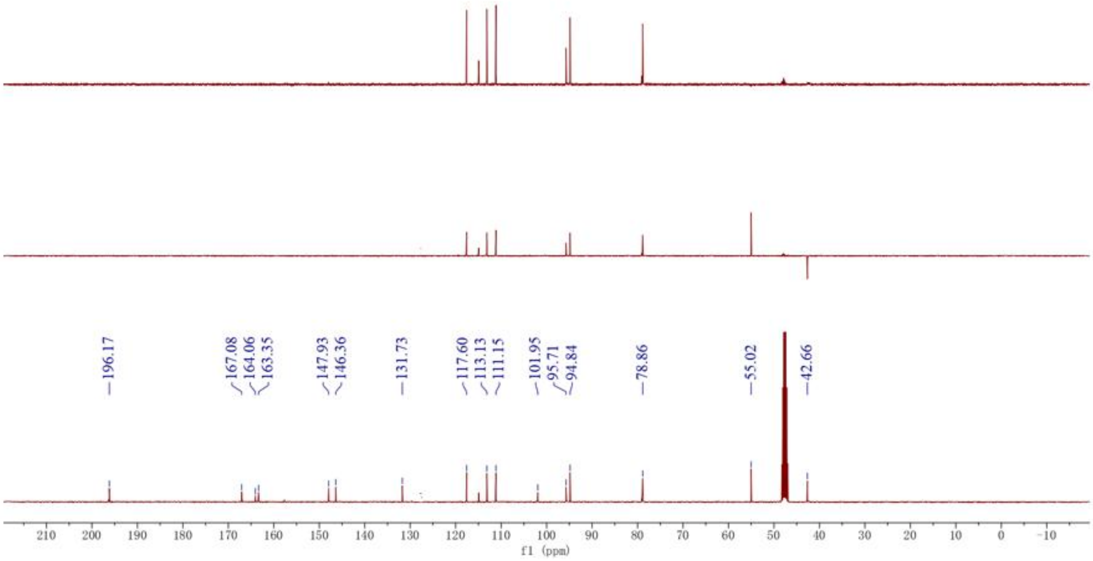

Figure S34. ^{13}C NMR (Bruker AV-400, 100 MHz, CD_3OD) of Hesperitin (17)

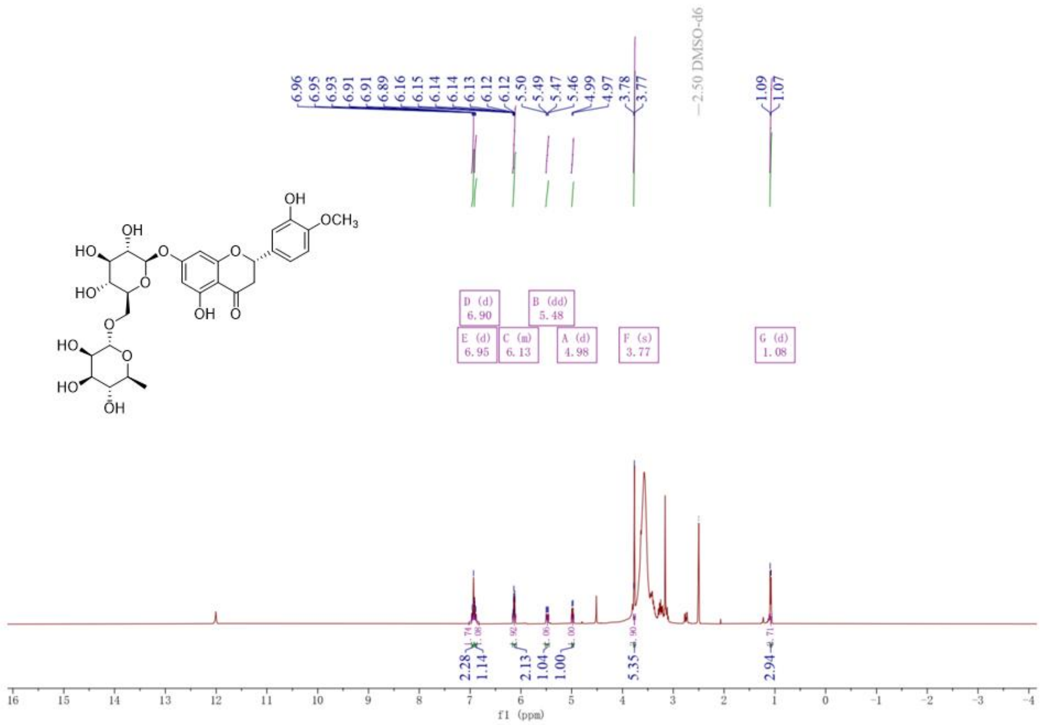

Figure S35. ^{1}H **NMR (Bruker AV-400, 400 MHz, DMSO-d$_6$) of Hesperidin (18)**

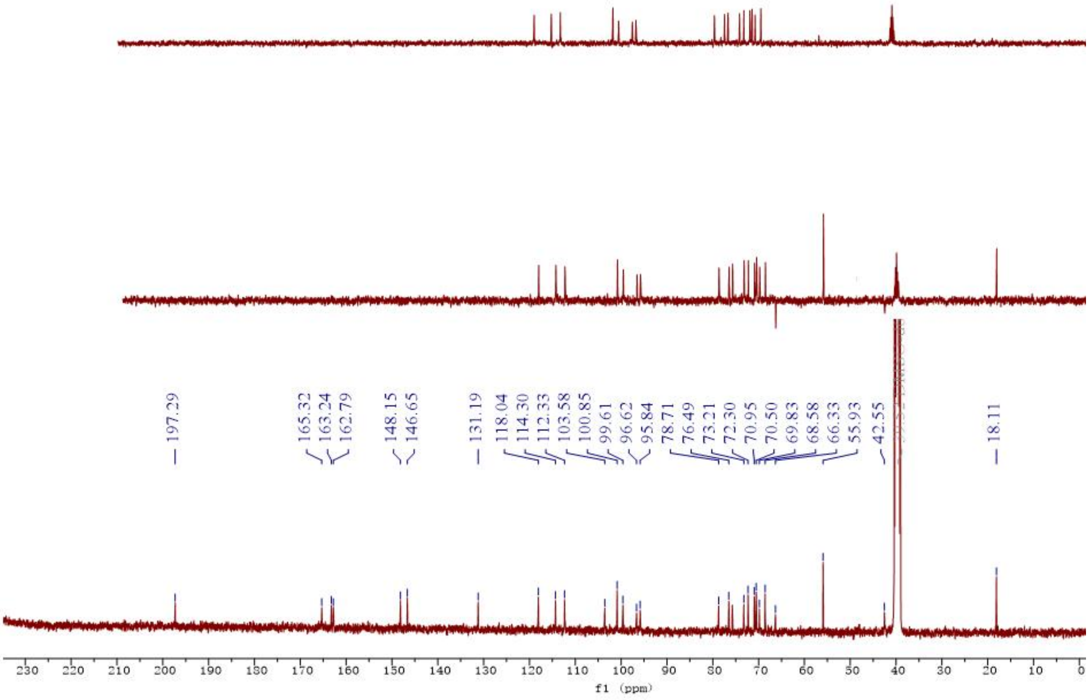

Figure S36. ^{13}C **NMR (Bruker AV-400, 100 MHz, DMSO-d$_6$) of Hesperidin (18)**

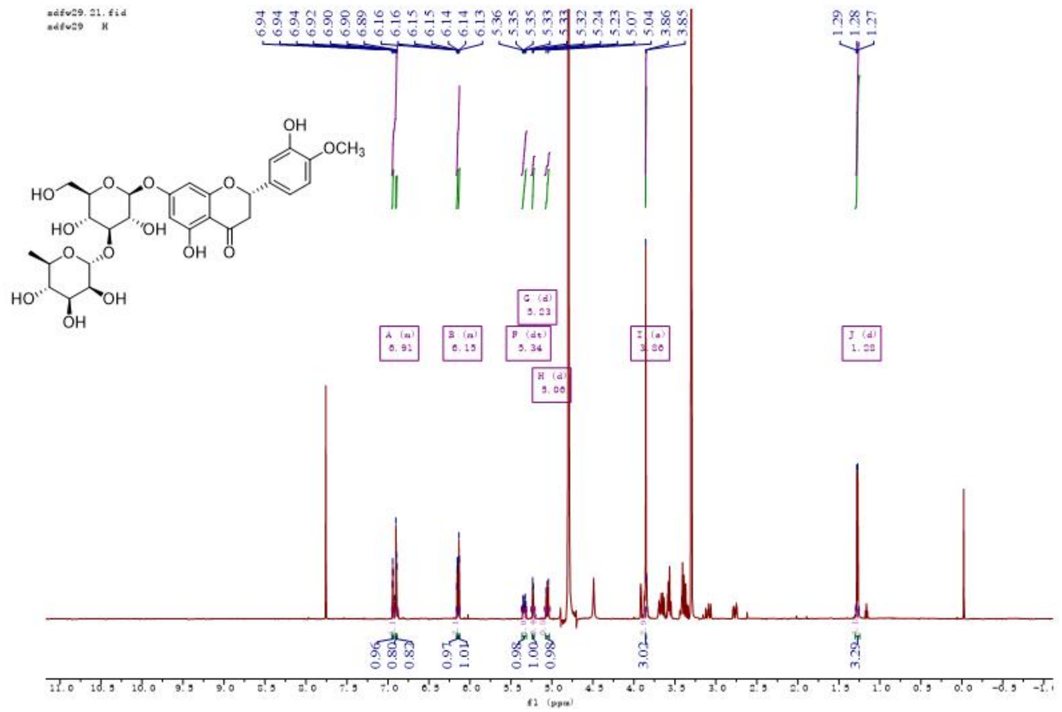

Figure S37. ^{1}H NMR (Bruker AV-500, 500 MHz, CD_3OD) of Neohesperidin (19)

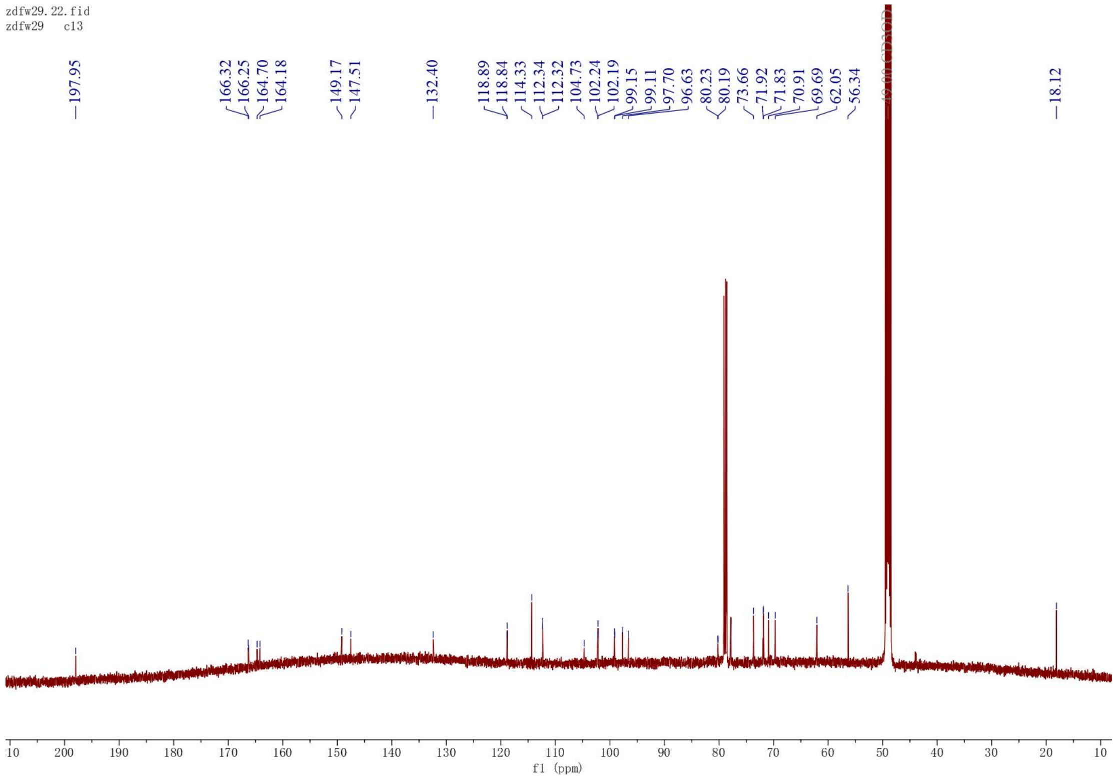

Figure S38. ^{13}C NMR (Bruker AV-500, 125 MHz, CD_3OD) of Hesperidin (19)

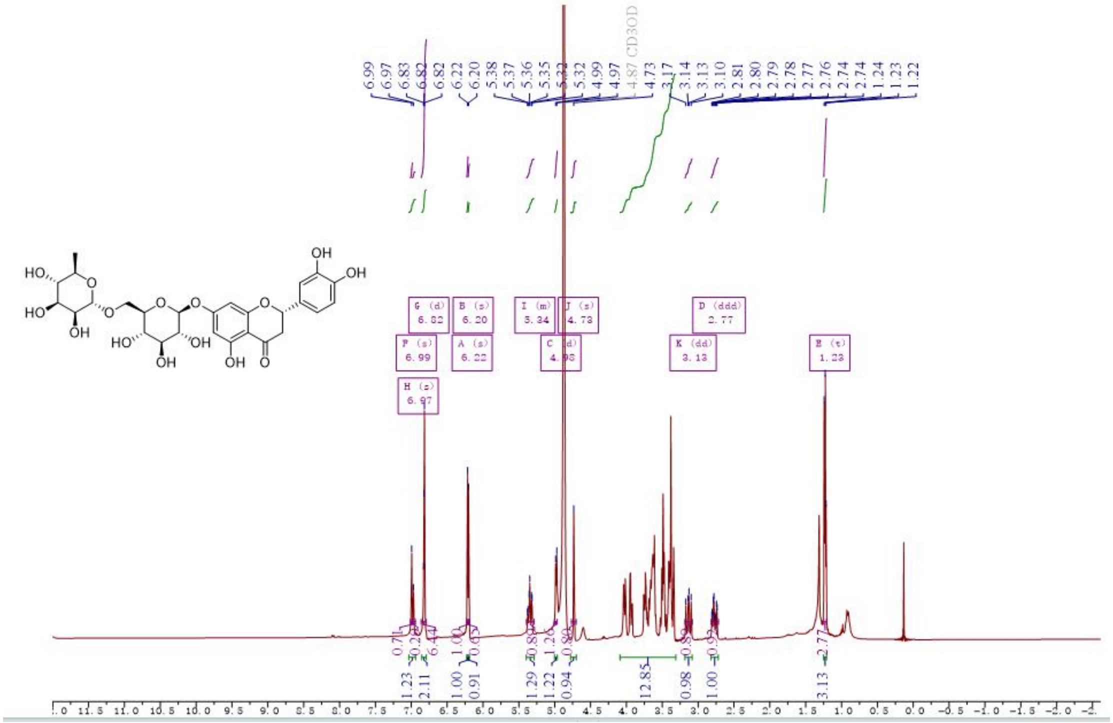

Figure S37. ^{1}H NMR (Bruker AV-400, 400 MHz, CD_3OD) of Eriodictioside (20)

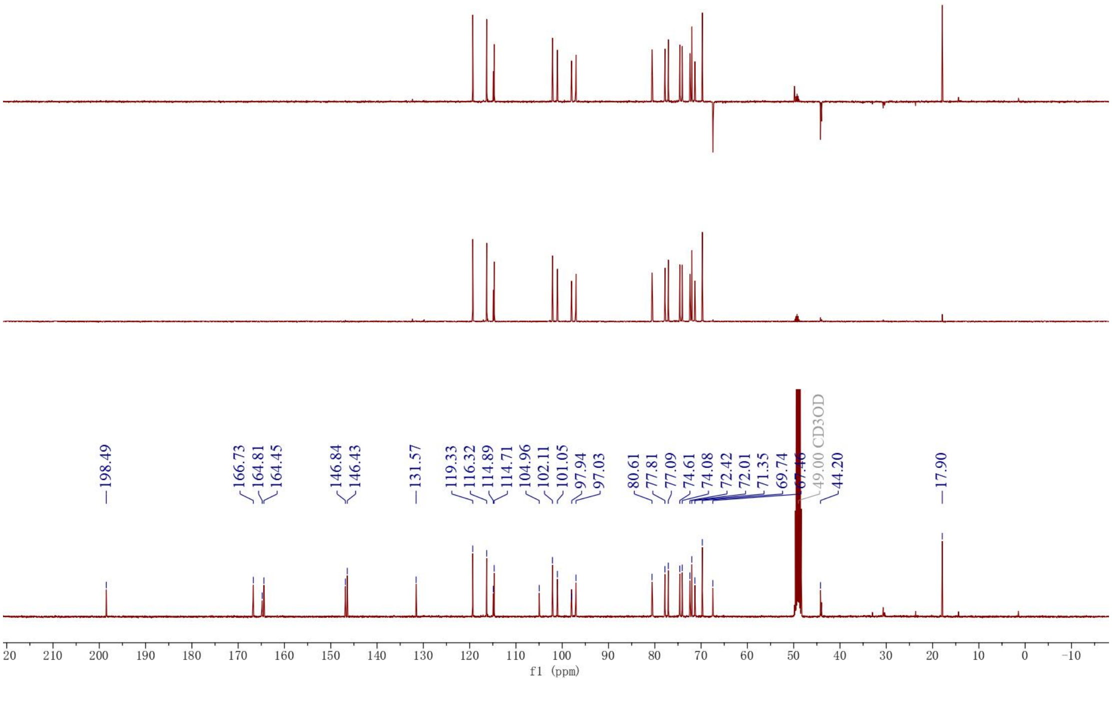

Figure S38. ^{13}C NMR (Bruker AV-400, 100 MHz, CD_3OD) of Eriodictioside (20)

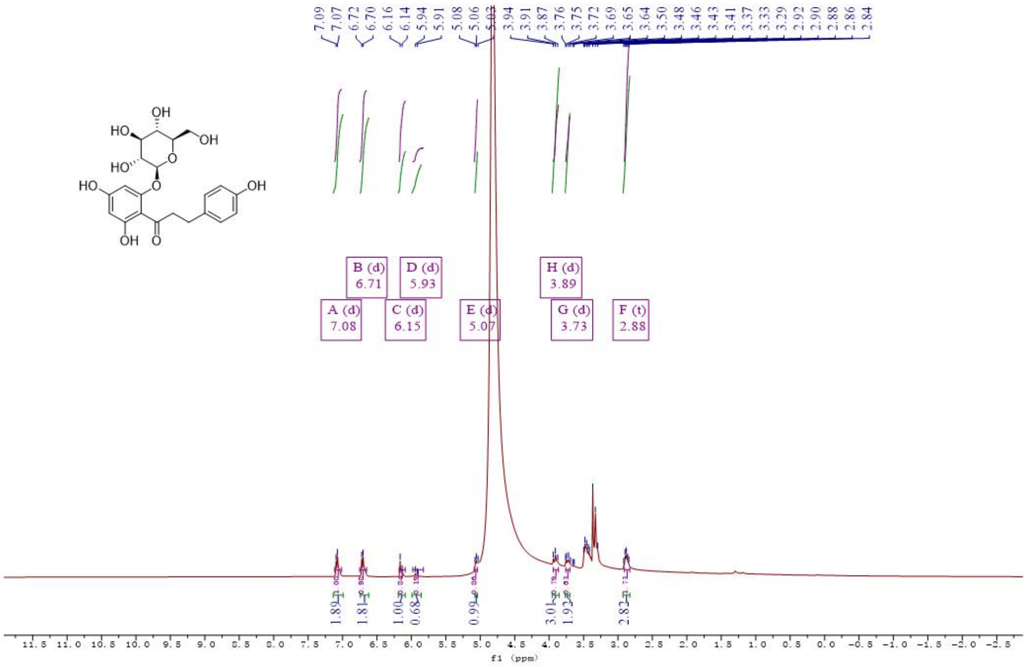

Figure S39. ^{1}H NMR (Bruker AV-400, 400 MHz, CD_3OD) of Phlorizin (21)

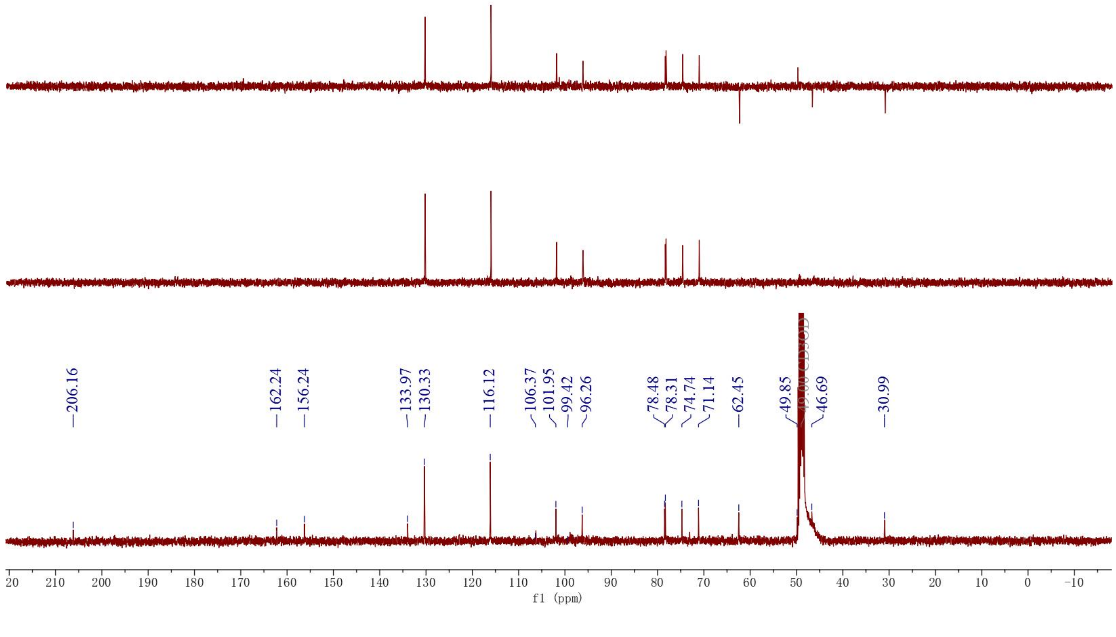

Figure S40. ^{13}C NMR (Bruker AV-400, 100 MHz, CD_3OD) of Phlorizin (21)

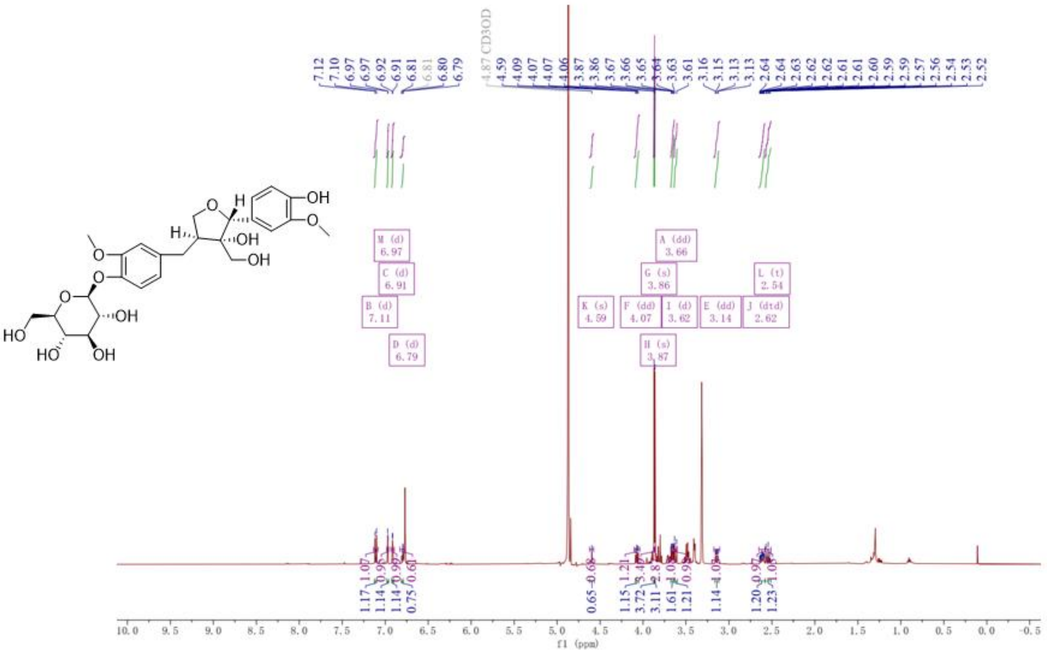

Figure S41. ^{1}H NMR (Bruker AV-400, 400 MHz, CD_3OD) of 8-Hydroxypinoresinol-4'-*O*-*β*-*D*-Glucopyranoside (22)

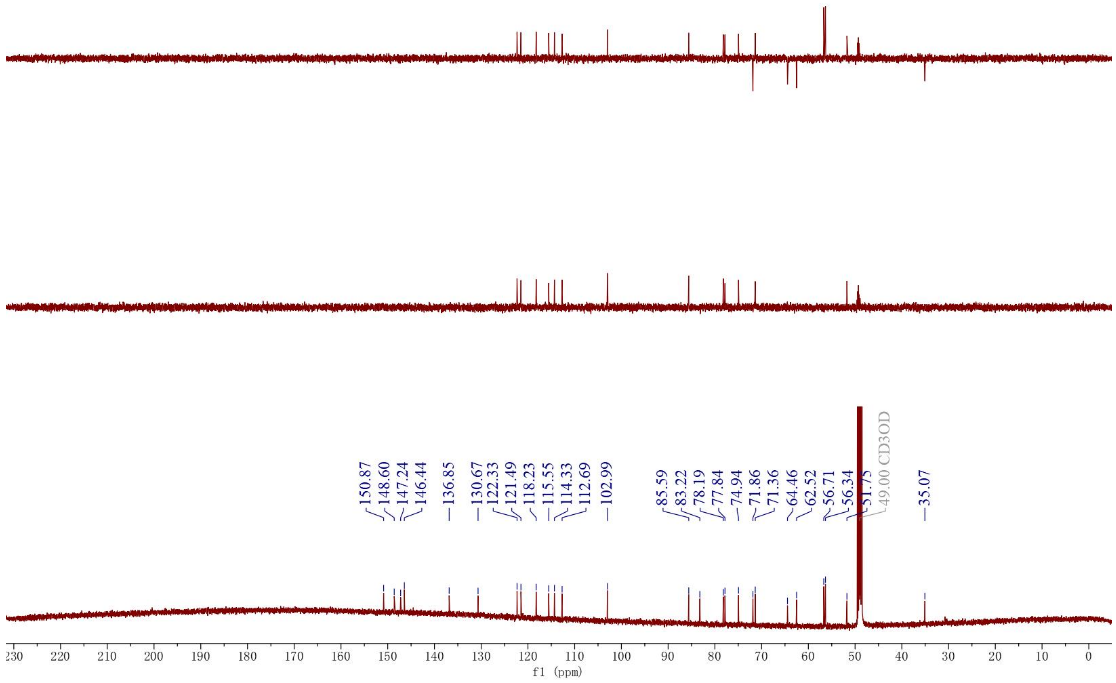

Figure S42. ^{13}C NMR (Bruker AV-400, 100 MHz, CD_3OD) of 8-Hydroxypinoresinol-4'-*O*-*β*-*D*-Glucopyranoside (22)

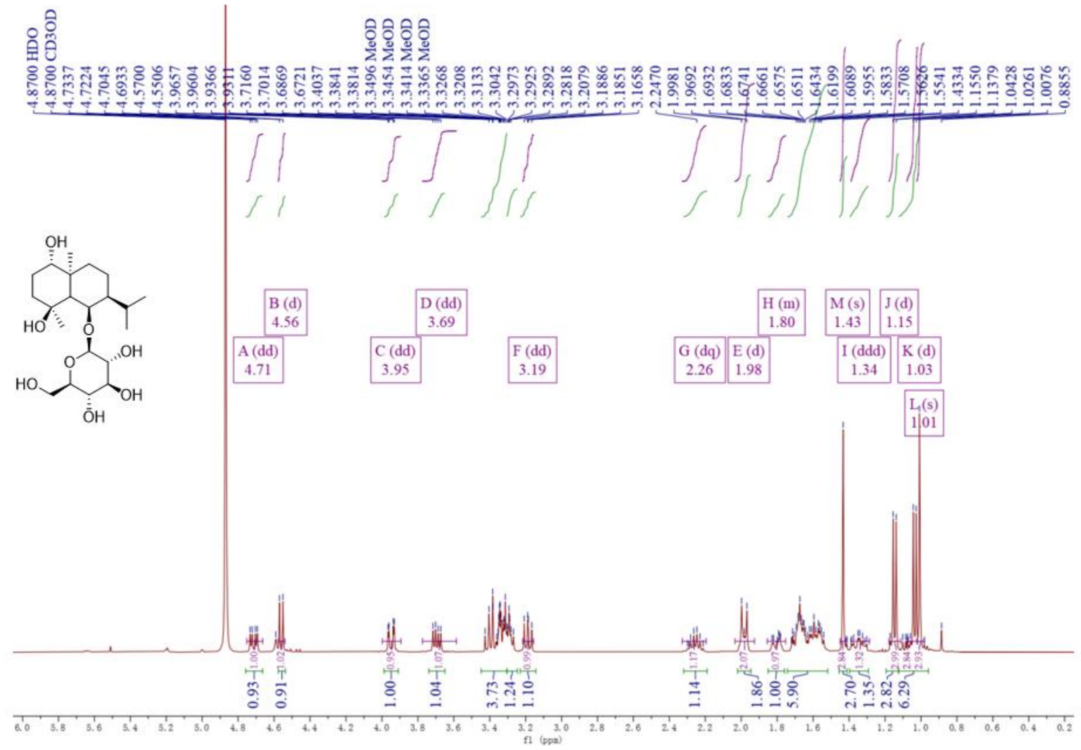

Figure S43. ^{1}H NMR (Bruker AV-400, 400 MHz, CD_3OD) of Pumilaside A (23)

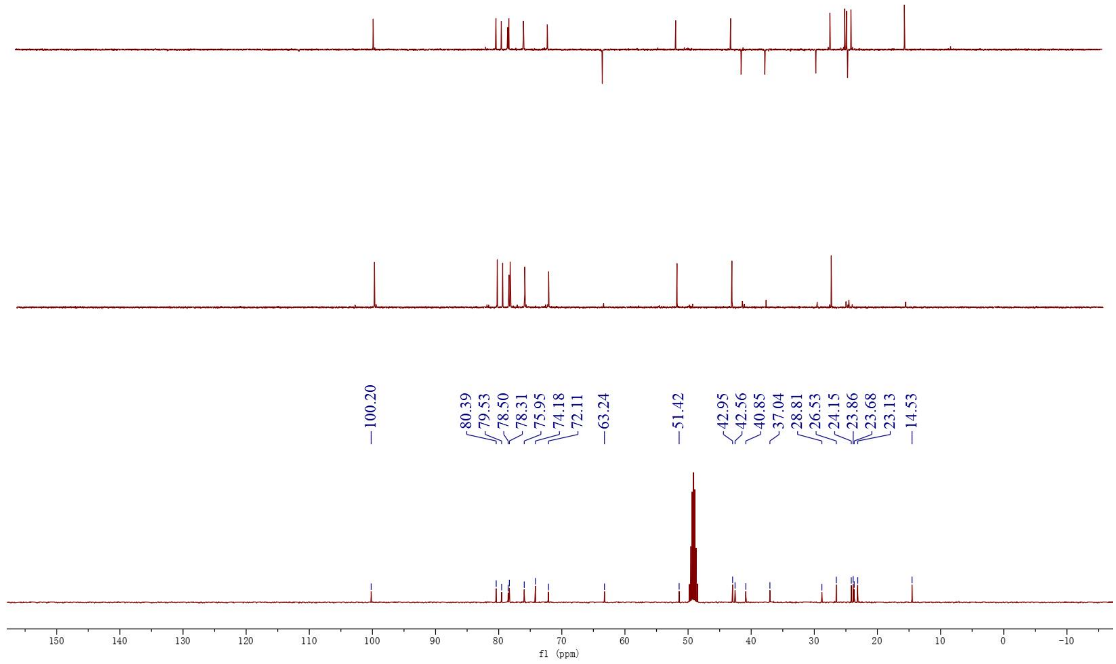

Figure S44. ^{13}C NMR (Bruker AV-400, 100 MHz, CD_3OD) of Pumilaside A (23)

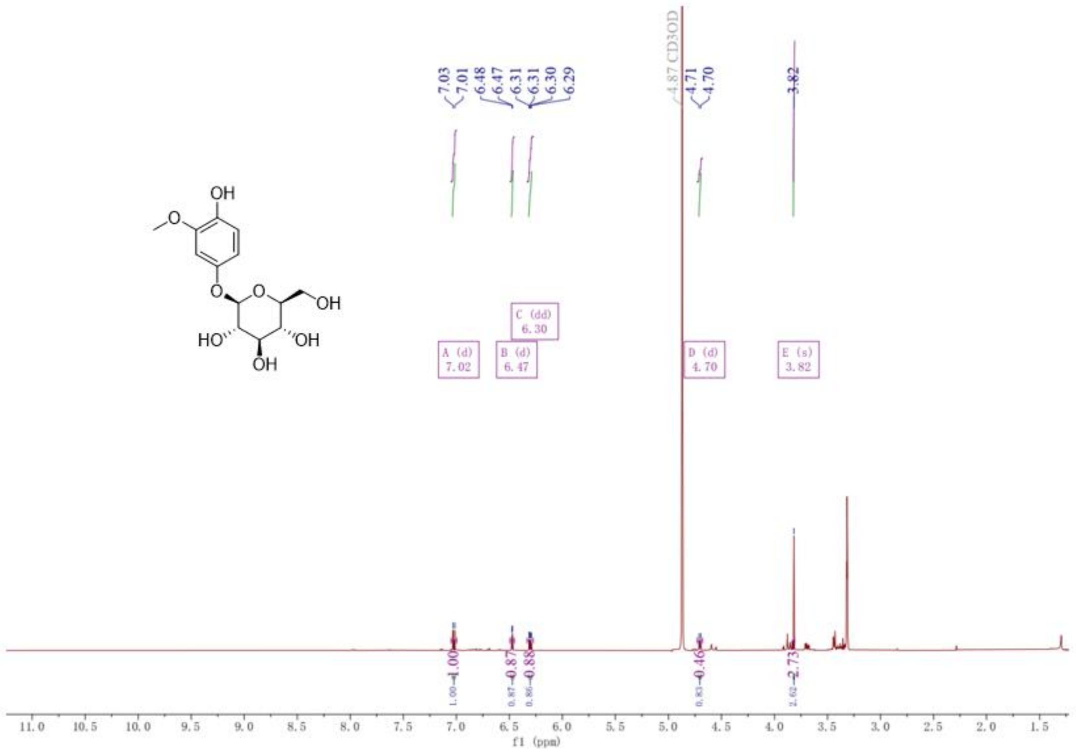

Figure S45. ^{1}H NMR (Bruker AV-400, 400 MHz, CD_3OD) of 4-hydroxy-2-methoxyphenol-1-*O*-*β*-*D*-glucopyranoside (24)

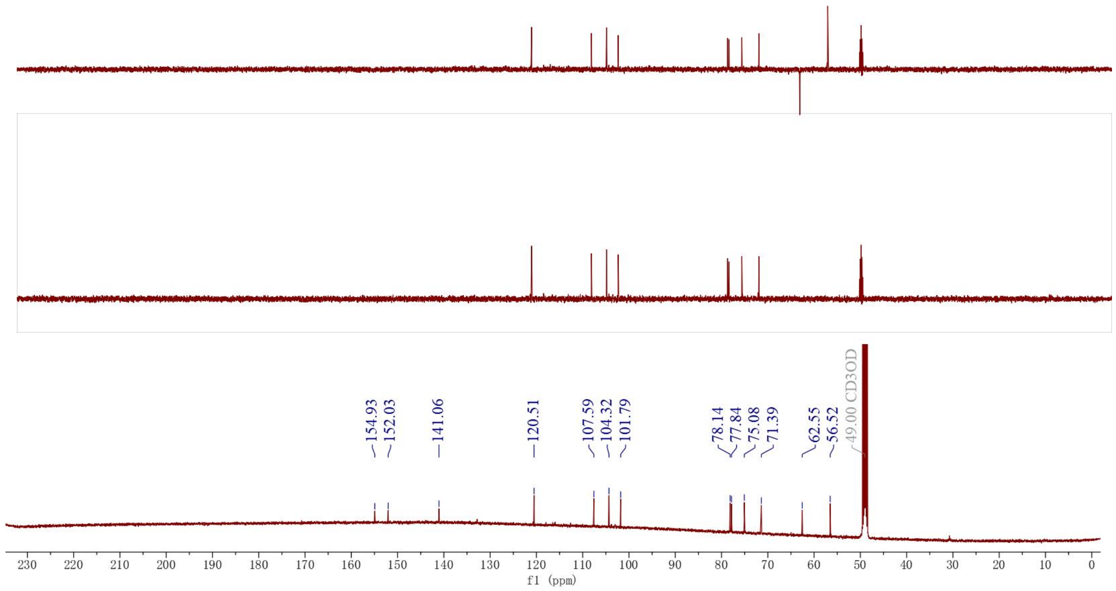

Figure S46. ^{13}C NMR (Bruker AV-400, 100 MHz, CD_3OD) of 4-hydroxy-2-methoxyphenol-1-*O*-*β*-*D*-glucopyranoside (24)

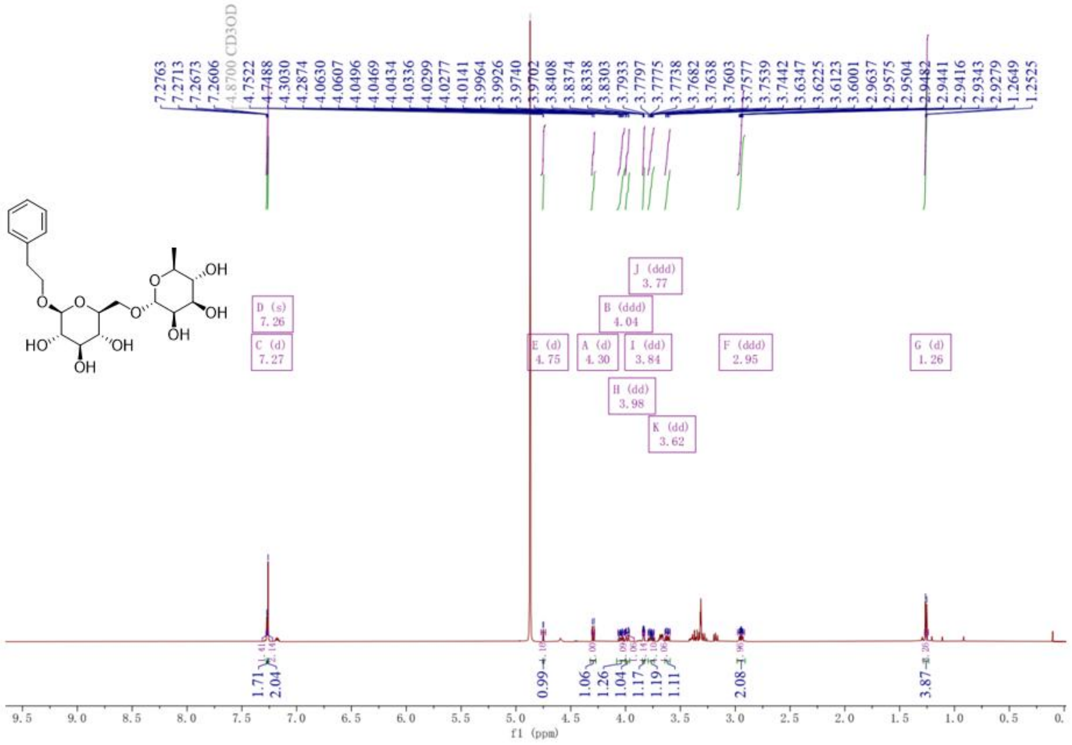

Figure S47. ^{1}H NMR (Bruker AV-400, 400 MHz, CD_3OD) of Phenylethyl-rutinoside (25)

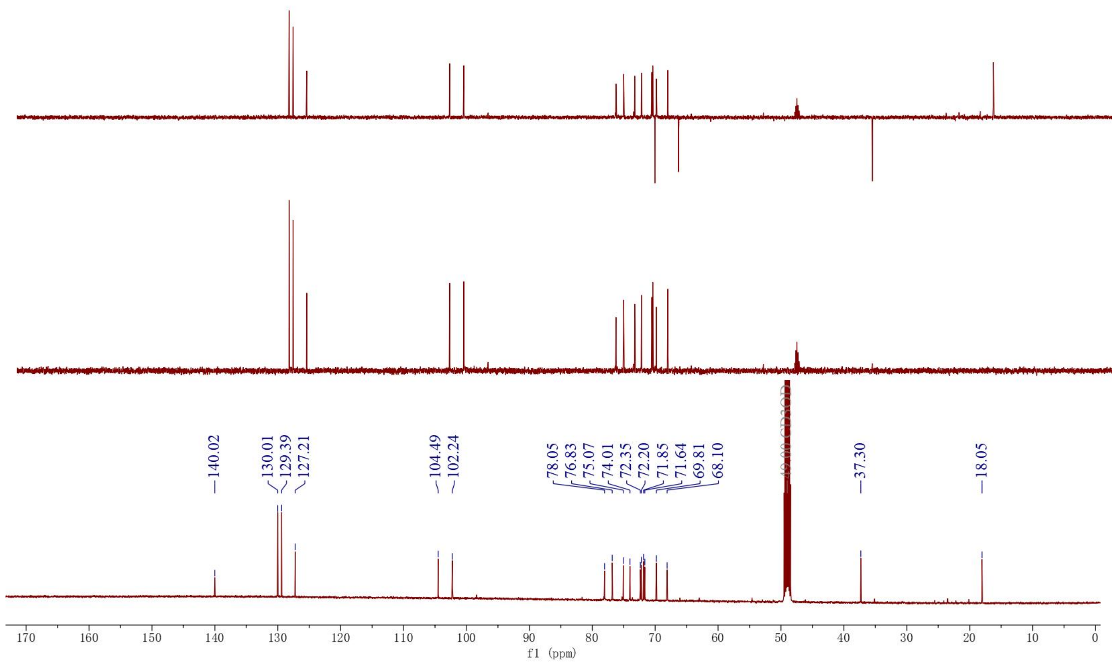

Figure S48. ^{13}C NMR (Bruker AV-400, 100 MHz, CD_3OD) of Phenylethyl-rutinoside (25)

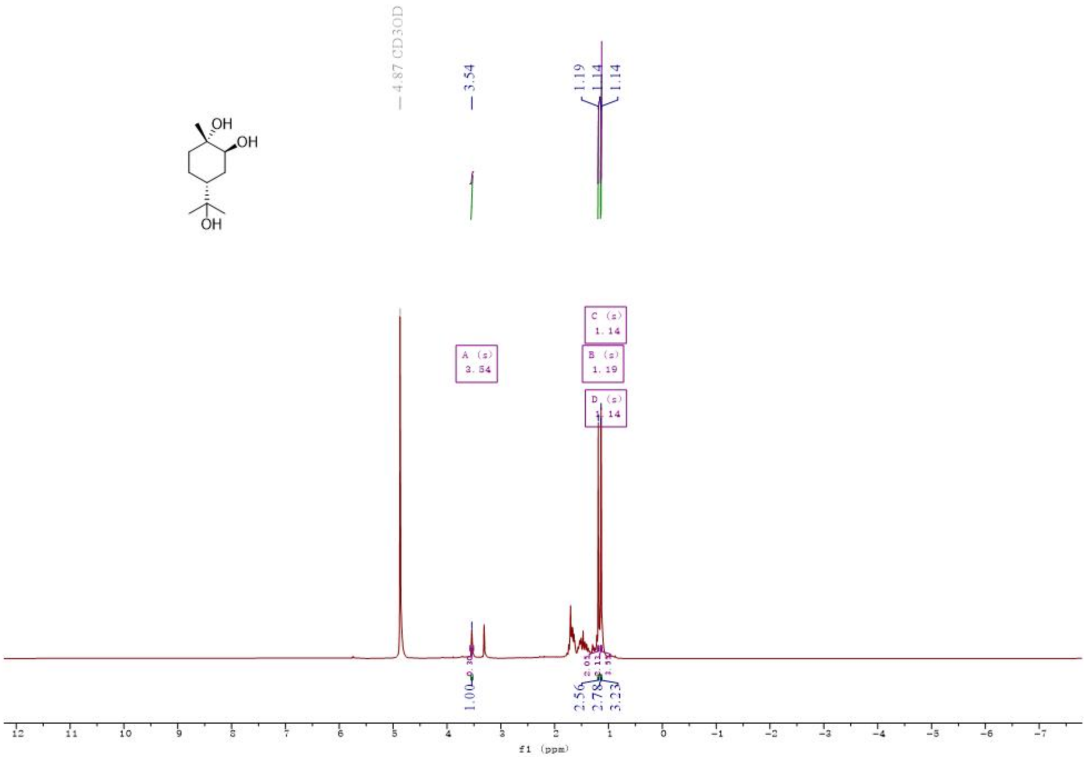

Figure S49. ^{1}**H NMR (Bruker AV-400, 400 MHz,** CD_3OD**) of Trans-*p*-menthane-1*α*,2*β*,8-triol (26)**

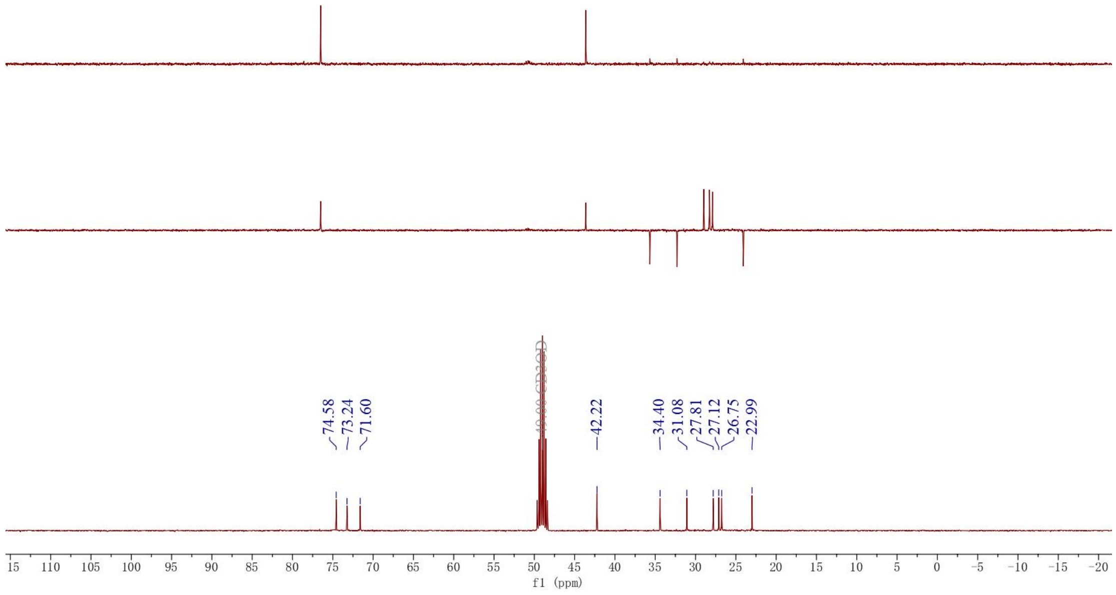

Figure S48. ^{13}**C NMR (Bruker AV-400, 100 MHz,** CD_3OD**) of Trans-*p*-menthane-1*α*,2*β*,8-triol (26)**

Callyspongidipeptide A (Calp, 1): White Powder, ^{1}H-NMR (CD_3OD, 400 MHz), *δ*: 4.22 (1H, t, *J* = 7.8 Hz, H-9), 4.08 (1H, t, *J* = 2.3 Hz, H-6), 3.60-3.53, 3.52-3.49 (2H, m, H-3), 2.35-2.02, 1.98-1.91 (2H, m, H-5), 2.20-2.16 (1H, m, H-10), 2.06-2.02, 1.98-1.91 (2H, m, H-4),1.49-1.43, 1.37-1.31 (2H, m, H-11), 1.07 (3H, t, *J* = 6.9 Hz, H-12), 0.95 (3H, t, *J* = 6.9 Hz, H-13); ^{13}C-NMR (CD_3OD, 100 MHz), *δ*: 172.4 (s, C-1), 46.2 (t, C-3), 23.2 (t, C-4), 29.6 (t, C-5), 61.3 (d, C-6), 167.6 (s, C-7), 60.0 (d, C-9), 37.1 (d, C-10), 25.5 (d, C-11), 15.5 (q, C-12), 12. 6 (q, C-13).

Hesperetin 7-*O*-*β*-*D*-glucopyranoside (Hesp, 2): yellow powder, ^{1}H-NMR (DMSO-d_6, 400 MHz) *δ*: 12.0 (1H, s, 5-OH), 9.1 (1H, s, 3'-OH), 6.95 (1H, d, *J* = 8.6 Hz, H-5'), 6.93 (1H, s, H-2'), 6.91 (1H, d, *J* = 8.6 Hz, H-6'), 6.14 (1H, d, *J* = 2.3 Hz, H-8), 6.12 (1H, d, *J* = 2.3 Hz, H-6), 5.52 (1H, dd, *J* = 12.2, 3.1 Hz, H-2), 3.77 (3H, s, H-OCH_3), 3.42 (1H, dd, *J* = 17.4, 12.0 Hz, H-3a), 2.77 (1H, dd, *J* = 17.4, 3.0 Hz, H-3b); ^{13}C-NMR (DMSO-*d_6*, 100 MHz) *δ*: 78.5 (C-2), 42.1 (C-3), 197.1 (C-4), 162.7 (C-5), 96.5 (C-6), 165.2 (C-7), 95.5 (C-8), 162.7 (C-9), 103.3 (C-10), 130.9 (C-1'), 114.1 (C-2'), 146.5 (C-3'), 148.0 (C-4'), 112.0 (C-5'), 117.9 (C-6'), 55.7 (C-OCH_3), 99.6 (C-1''), 73.0 (C-2''), 77.1 (C-3''), 69.5 (C-4''), 76.3 (C-5''), 60.6 (C-6'').

Limonin (3): White Powder, ^{1}H-NMR (DMSO-*d_6*, 400 MHz) *δ*: 7.73 (1H, s, H-21), 7.67 (1H, s, H-23), 6.52 (1H, s, H-22), 5.49 (1H, s, H-17), 4.93 (1H, d, *J* = 13.0 Hz, H-19*β*), 4.49 (1H, d, *J* = 13.0 Hz, H-19*α*), 4.12 (2H, s, H-1, H-15), 3.13 (1H, d, *J* = 15.0 Hz, H-2*β*), 2.78 (1H, d, *J* = 15.0 Hz, H-2*α*), 2.60 (1H, dd, *J* = 14.7, 3.7 Hz, H-6*β*), 2.56 (1H, d, *J* = 12.6 Hz, H-9), 2.51 (1H, m, H-5), 2.29 (1H, dd, *J* = 14.7, 2.6 Hz, H-6*α*), 1.73 (2H, m, H-11), 1.26 (2H, m, H-12), 1.20 (3H, s, H-28), 1.12 (3H, s, H-18), 1.03(3H, s, H-29), 1.01(3H, s, H-30); ^{13}C-NMR (DMSO-d_6, 100 MHz) *δ*: 78.4 (C-1), 35.7 (C-2), 170.2 (C-3), 79.5 (C-4), 58.0 (C-5), 36.2 (C-6), 208.0 (C-7), 50.3 (C-8), 46.5 (C-9), 45.3 (C-10), 17.5 (C-11), 29.2 (C-12), 37.6 (C-13), 66.7 (C-14), 53.7 (C-15), 167.3 (C-16), 77.4 (C-17), 29.8 (C-18), 64.8 (C-19), 120.2 (C-20), 141.7 (C-21), 110.2 (C-22), 143.4 (C-23), 21.4 (C-28), 19.7 (C-29), 17.0 (C-30).

Obacunone 17-*O*-*β*-D-glucopyranoside, (4): White Powder, ^{1}H-NMR (CD_3OD, 400 MHz) *δ*: 6.47 (1H, d, *J* = 12.8 Hz, H-1), 5.98 (1H, d, *J* = 12.8 Hz, H-2), 3.15 (1H, m, H-5), 2.92 (1H, m, H-6a), 2.37 (1H, m, H-6b), 2.59 (1H, m, H-9), 1.83 (2H, m, H-11), 2.11 (1H, m, H-12a), 1.74 (1H, m, H-12b), 2.91 (1H, s, H-15), 5.45 (1H, s, H-17), 1.46 (3H, s, 18), 1.14 (3H, s, H-19), 7.74 (1H, br s, H-21), 6.65 (1H, br s, H-22), 7.39 (1H, br s, H-23), 0.88 (3H, s, H-24), 1.49 (3H, s, H-25), 1.39 (3H, s, H-26), 4.35 (1H, d, *J* = 7.8 Hz, H-1'), 3.14 (1H, m, H-2'), 3.19 (1H, m, H-3'), 3.27 (1H, m, H-4'), 3.32 (1H, m, H-5'), 3.65, 3.50 (2H, m, H-6'); ^{13}C-NMR (CD_3OD, 100 MHz) *δ*: 152.6 (C-1), 120.0 (C-2), 169.1 (C-3), 85.7

(C-4), 52.1 (C-5), 41.8 (C-6), 212.9 (C-7), 53.6 (C-8), 48.8 (C-9), 46.2 (C-10), 19.1 (C-11), 28.6 (C-12), 46.0 (C-13), 71.2 (C-14), 61.2 (C-15), 175.8 (C-16), 79.9 (C-17), 26.4 (C-18), 15.9 (C-19), 127.0 (C-20), 143.7 (C-21), 114.0 (C-22), 142.0 (C-23), 20.8 (C-24), 30.2 (C-25), 23.4 (C-26), 105.7 (C-1'), 75.8 (C-2'), 77.4 (C-3'), 71.8 (C-4'), 78.4 (C-5'), 62.9 (C-6').

Diosmetin, (5): yellow powder, ^{1}H-NMR (CD_3OD, 400 MHz) δ: 7.48 (1H, dd, J = 8.5, 2.3 Hz, H-6'), 7.46 (1H, d, J = 2.3 Hz, H-2'), 7.36 (1H, d, J = 8.5 Hz, H-5'), 7.04 (1H, s, H-3), 6.55 (1H, s, H-8), 6.19 (1H, s, H-6), 3.93 (3H, s, H-OCH_3); ^{13}C-NMR (CD_3OD, 100 MHz) δ: 165.9 (C-2), 105.3 (C-3), 183.8 (C-4), 159.4 (C-5), 100.2 (C-6), 166.1 (C-7), 95.0 (C-8), 162.8 (C-9), 104.4 (C-10), 120.0 (C-1'), 112.6 (C-2'), 148.2 (C-3'), 152.6 (C-4'), 113.8 (C-5'), 124.9 (C-6'), 56.7 (C-OCH_3).

Luteolin, (6): yellow powder, ^{1}H-NMR (CD_3OD, 400 MHz) δ_H: 7.95 (d, J = 2.5 Hz, H-2'), 7.43 (dd, J = 8.0, 2.5 Hz, H-6'), 6.89 (dd, J = 8.0, 2.5 Hz, H-5'), 6.69 (s, H-3), 6.46 (d, J = 2.5 Hz, H-8), 6.21 (d, J = 2.5 Hz, H-6); ^{13}C-NMR (CD_3OD, 100 MHz) δ_C: 163.7 (C-2), 103.5 (C-3), 181.4 (C-4), 157.1 (C-5), 98.6 (C-6), 164.0 (C-7), 93.6 (C-8), 161.3 (C-9), 103.5 (C-10), 121.3 (C-1'), 114.3 (C-2'), 145.6 (C-3'), 149.5 (C-4'), 115.8 (C-5'), 118.9 (C-6').

Isoquercetin, (7): yellow powder, ^{1}H-NMR (CD_3OD, 400 MHz) δ: 7.71 (1 H, s, H-2′), 7.58 (1 H, d, J = 8.1 Hz, H-5′), 6.86 (1 H, d, J = 8.5 Hz, H-6′), 6.37 (1 H, s, H-8), 6.18 (1 H, s, H-6), 5.24 (1 H, d, J = 7.4 Hz, H-1″), 3.72 ~3.34 (6 H, m, Glu-H); ^{13}C-NMR (CD_3OD, 100 MHz) δ: 179.5 (C-4), 166.1 (C-7), 163.0 (C-5), 159.0 (C-2), 158.4 (C-9) , 149.8 (C-4′), 145.9 (C-3′), 135.6 (C-3), 123.2 (C-1′), 123.0 (C-6'), 117.6 (C-3′), 116.0 (C-5′), 105.6 (C-10), 104.1 (C-1''), 99.9 (C-6), 94.7 (C-8), 78.4 (C-5''), 78.1 (C-3''), 75.7 (C-2''), 71.2 (C-4''), 62.5 (C-6'').

Nobiletin, (8): yellow powder, ^{1}H-NMR (CD_3OD, 400 MHz) δ: 7.64 (1H, dd, J = 8.5, 2.2 Hz, H-6'), 7.53 (1H, d, J = 2.2 Hz, H-2'), 7.12 (d, J = 8.6 Hz, H-5'), 6.68 (1H, s, H-3), 4.12, 4.04, 3.94, 3.93, 3.92, 3.90 (18H, H-6 × OCH_3); ^{13}C-NMR (CD_3OD, 100 MHz) δ: 163.6 (C-2), 107.0 (C-3), 179.6 (C-4), 149.4 (C-5), 145.6 (C-6), 153.4 (C-7), 139.6 (C-8), 149.2 (C-9), 115.4 (C-10), 124.8 (C-1'), 110.3 (C-2'), 150.9 (C-3'), 153.9 (C-4'), 112.8 (C-5'), 121.2 (C-6'), 62.7, 62.6, 62.2, 62.1, 56.6, 56.5 (6 × OCH_3).

Isosakuranetin, (9): yellow powder, ^{1}H-NMR (CD_3OD, 400 MHz) δ: 7.41 (2H, d, J = 8.6 Hz, H-2', 6'), 6.97 (2H, d, J = 8.6 Hz, H-3', 5'), 5.94 (1H, br s, H-8), 5.92 (1H, br s, H-6), 5.38 (1H, dd, J = 12.9, 3.1 Hz, H-2), 3.83 (3H, s, H-OCH_3), 3.08 (1H, dd, J = 17.2, 13.2 Hz, H-3α), 2.74 (1H, dd, J =

17.2, 3.2 Hz, H-3*β*); ^{13}C-NMR (CD_3OD, 100 MHz) *δ*: 80.3 (C-2), 43.9 (C-3), 197.4 (C-4), 165.2 (C-5), 97.1 (C-6), 168.2 (C-7), 96.2 (C-8), 164.6 (C-9), 103.3 (C-10), 132.0 (C-1'), 128.8 (C-2', 6'), 114.9 (C-3', 5'), 161.2 (C-4'), 55.7 (C-OCH_3).

Epicatechin, (10): yellow powder, ^{1}H-NMR (CD_3OD, 400 MHz) *δ*: 7.02 (1H, d, *J* = 1.9 Hz, H-2'), 6.85 (1H, d, *J* = 8.2, 2.0 Hz, H-6'), 6.82 (1H, br s, H-5'), 5.99 (1H, d, *J* = 2.2 Hz, H-6), 5.97 (1H, d, *J* = 2.2 Hz, H-8), 4.23 (1H, br s, H-3), 2.91 (1H, dd, *J* = 16.7, 4.7 Hz, H-4*α*), 2.78 (1H, dd, *J* = 16.8, 3.1Hz, H-4*β*); ^{13}C-NMR (CD_3OD, 100 MHz) *δ*: 79.8 (C-2), 67.5 (C-3), 29.2 (C-4), 157.6 (C-5), 95.9 (C-6), 157.3 (C-7), 96.4 (C-8), 157.9 (C-9), 100.1 (C-10), 132.3 (C-1'), 115.3 (C-2'), 145.7 (C-3'), 145.9 (C-4'), 115.9 (C-5'), 119.4 (C-6').

Kaempferol, (11): yellow powder, ^{1}H-NMR (CD_3OD, 400 MHz) δ_H: 8.09 (2H, d, *J* = 8.6 Hz, H-2', 6'), 6.92 (2H, d, *J* = 8.6 Hz, H-3', 5'), 6.40 (1H, d, *J* = 2.5 Hz, H-8), 6.19 (1H,d, *J* = 2.5 Hz, H-6); ^{13}C-NMR (CD_3OD, 100 MHz) δ_C: 158.1 (C-2), 136.9 (C-3), 177.2 (C-4), 160.3 (C-5), 99.1 (C-6), 165.4 (C-7), 94.3 (C-8), 158.1 (C-9), 104.3 (C-10), 123.5 (C-1'), 130.5 (C-2', 6'), 116.1 (C-3', 5'), 162.3 (C-4').

Kaempferol-3-*O*-*β*-*D*-glucopyranoside, (12): yellow powder, ^{1}H-NMR (CD_3OD, 400 MHz) δ_H: 8.05 (2H, d, *J* = 8.6 Hz, H-2', 6'), 6.88 (2H, d, *J* = 8.6 Hz, H-3', 5'), 6.38 (1H, d, *J* = 2.0 Hz, H-8), 6.20 (1H, d, *J* = 2.0 Hz, H-6), 5.23 (1H, d, *J* = 6.7 Hz, H-1''); ^{13}C-NMR (CD_3OD, 100 MHz) δ_C: 158.4 (C-2), 135.5 (C-3), 179.5 (C-4), 163.0 (C-5), 99.9 (C-6), 166.0 (C-7), 94.8 (C-8), 159.1 (C-9), 105.7 (C-10), 122.8 (C-1'), 132.3 (C-2', 6'), 116.1 (C-3', 5'), 161.5 (C-4'), 104.2 (C-1''), 75.7 (C-2''), 78.0 (C-3''), 71.4 (C-4''), 78.3 (C-5''), 62.7 (C-6'').

Kaempferol-3-*O*-(6''-*O*-acetyl) -*β*-*D*-glucopyranoside, (13): yellow powder, ^{1}H-NMR (CD_3OD, 400 MHz) δ_H: 8.00 (2H, d, *J* = 8.8 Hz, H-2', 6'), 6.88 (2H, d, *J* = 8.8 Hz, H-3', 5'), 6.43 (1H, d, *J* = 1.8 Hz, H-8), 6.20 (1H, d, *J* = 1.8 Hz, H-6), 5.36 (1H, d, *J* = 7.1 Hz, H-1''), 1.75 (3H, s, H-CH_3); ^{13}C-NMR (CD_3OD, 100 MHz) δ_C: 156.9 (C-2), 133.0 (C-3), 177.3 (C-4), 161.2 (C-5), 98.9 (C-6), 165.0 (C-7), 93.8 (C-8), 156.5 (C-9), 103.6 (C-10), 120.7 (C-1'), 130.8 (C-2', 6'), 115.1 (C-3', 5'), 160.1 (C-4'), 101.2 (C-1''), 74.1 (C-2''), 76.2 (C-3''), 69.8 (C-4''), 73.9 (C-5''), 62.8 (C-6''), 169.9 (C-CH_3CO), 20.2 (C-CH_3).

Kaempferol-3-*O*-glucosyl-6''-*O*-pentadionic acid, (14): yellow oil, ^{1}H-NMR (CD_3OD, 400 MHz) δ_H: 8.05 (2H, d, *J* = 8.9 Hz, H-2', 6'), 6.89 (2H, d, *J* = 8.9 Hz, H-3', 5'), 6.43 (1H, d, *J* = 2.0 Hz, H-8), 6.23 (1H, d, *J* = 2.0 Hz, H-6), 5.43 (1H, d, *J* = 7.2 Hz, H-1''), 3.63 (3H, s, 5'''-OCH_3), 1.21 (3H, s,

6'''-CH$_3$); ^{13}C-NMR (CD$_3$OD, 100 MHz) δ_C: 159.3 (C-2), 135.2 (C-3), 179.4 (C-4), 163.1 (C-5), 100.0 (C-6), 166.1 (C-7), 94.9 (C-8), 158.5 (C-9), 105.7 (C-10), 122.8 (C-1'), 132.3 (C-2', 6'), 116.1 (C-3', 5'), 161.6 (C-4'), 103.9 (C-1''), 75.7 (C-2'', 5''), 77.9 (C-3''), 71.5 (C-4''), 64.6 (C-6''), 172.2 (C-1'''), 46.3 (C-2'''), 70.7 (C-3'''), 45.9 (C-4'''), 173.1 (C-5'''), 27.7 (C-6'''), 52.0 ($C_{5'''}$-OCH$_3$).

5, 7, 4′-trihydroxy-8, 3′-dimethoxyflavone-3-*O*-6″-3-hydroxyl-3-methylglutaroyl)-*β*-*D*-glucop -yranoside, (15): yellow oil, ^{1}H-NMR (CD$_3$OD, 400 MHz) δ_H: 7.90 (1H, s, H-2'), 7.67 (1H, d, J = 8.2 Hz, H-6'), 6.86 (1H, d, J = 8.2 Hz, H-5'), 5.27 (1H, d, J = 7.0 Hz, H-1''), 4.20 (1H, d, J = 11.7 Hz, H-6''a), 4.09 (1H, m, H-6''b), 3.92 (3H, s, 8-OCH$_3$), 3.86 (3H, d, 3'-OCH$_3$), 3.59~3.45 (4H, m, H-2'', 3'', 4'', 5''), 2.46 (4H, m, H-2''', 4'''), 1.16 (3H, s, H-6'''); ^{13}C-NMR (CD$_3$OD, 100 MHz) δ_C: 158.5 (C-2), 135.3 (C-3), 179.3 (C-4), 157.7 (C-5), 100.1 (C-6), 158.4 (C-7), 129.0 (C-8), 62.0 (C_8-OCH$_3$), 149.7 (C-9), 105.5 (C-10), 122.9 (C-1'), 114.1 (C-2'), 148.3 (C-3'), 56.6 ($C_{3'}$-OCH3), 150.9 (C-4'), 116.1 (C-5'), 124.0 (C-6'), 104.1 (C-1''), 71.4 (C-2''), 77.8 (C-3''), 70.6 (C-4''), 73.8 (C-5''), 64.3 (C-6''), 172.2 (C-1'''), 46.2 (C-2'''), 46.2 (C-4'''), 175.8 (C-5'''), 27.5 (C-6''').

5, 7, 4′-trihydroxy-6, 8, 3′-dimethoxyflavone-3-*O*-6″-3-hydroxyl-3-methylglutaroyl)-*β*-*D*-gluc -opyranoside, (16): yellow oil, ^{1}H-NMR (CD$_3$OD, 400 MHz) δ_H: 7.98 (1H, d, J = 1.9 Hz, H-1'), 7.56 (1H, dd, J = 6.8, 1.6Hz, H-6'), 6.9 (1H, d, J = 6.8 Hz), 5.35 (1H, d, J = 7.2 Hz), 4.25 (1H, d, J = 11.9 Hz, H-6''a), 4.13 (1H, dd, J = 11.9, 5.7 Hz, H-6''b), 3.93 (3H, s, 6-OCH$_3$), 3.97 (3H, s, 8-OCH$_3$), 3.99 (3H, s, 3''-OCH$_3$), 3.58~3.42 (4H, m, H-2'', 3'', 4'', 5''), 2.47 (4H, m, H-2'', 4''), 1.20 (3H, s, H-6'''); ^{13}C-NMR (CD$_3$OD, 100 MHz) δ_C: 158.7 (C-2), 135.0 (C-3), 179.6 (C-4), 153.2 (C-5), 133.0 (C-6), 149.5 (C-7), 129.3 (C-8), 146.4 (C-9), 105.1 (C-10), 123.1 (C-1'), 113.8 (C-2'), 148.4 (C-3'), 151.1 (C-4'), 116.2 (C-5'), 124.1 (C-6'), 103.7 (C-1''), 75.8 (C-2''), 77.9 (C-3''), 70.6 (C-4''), 75.7 (C-5''), 64.3 (C-6''), 172.3 (C-1'''), 46.5 (C-2'''), 70.6 (C-3'''), 46.4 (C-4'''), 174.5 (C-5'''), 27.6 (C-6''').

Hesperitin, (17): yellow powder, ^{1}H-NMR (CD$_3$OD, 400 MHz) δ: 6.94~6.85 (3H, m, H-2', 5', 6'), 5.88 (1H, d, J = 2.0 Hz, H-8), 5.86 (1H, d, J = 2.0 Hz, H-6), 5.28 (1H, dd, J = 12.7, 3.2 Hz, H-2), 3.83 (3H, s, H-OCH$_3$), 3.03 (1H, dd, J = 17.2, 12.6 Hz, H-3α), 2.68 (1H, dd, J = 17.2, 3.1 Hz, H-3β); ^{13}C-NMR (CD$_3$OD, 100MHz) δ: 78.9 (C-2), 42.7 (C-3), 196.2 (C-4), 164.1 (C-5), 95.7 (C-6), 167.1 (C-7), 94.8 (C-8), 163.4 (C-9), 101.9 (C-10), 131.7 (C-1'), 113.1 (C-2'), 146.4 (C-3'), 147.9 (C-4'), 111.2 (C-5'), 117.6 (C-6'), 55.0 (C-OCH$_3$).

Hesperidin, (18): yellow powder, ^{1}H-NMR (DMSO-d_6, 400 MHz) δ: 6.95 (1H, m, H-6'), 6.90 (1H, m, H-2'), 6.13 (1H, d, J = 8.5 Hz, H-5'), 6.13 (1H, d, J = 2.1 Hz, H-6), 5.48 (1H, d, J = 2.1 Hz, H-8),

4.98 (1H, dd, J = 12.8, 3.0 Hz, H-2), 4.80 (1H, d, J = 7.4 Hz, H-1''), 4.52 (1H, br s, H-1'''), 3.77 (3H, s, H-OCH_3), 1.08 (3H, s, H-6'''); ^{13}C-NMR (DMSO-d_6, 100 MHz) δ: 78.1 (C-2), 42.6 (C-3), 197.3 (C-4), 163.2 (C-5), 99.6 (C-6), 165.3 (C-7), 96.6 (C-8), 162.8 (C-9), 103.6 (C-10), 131.2 (C-1'), 114.3 (C-2'), 148.2 (C-3'), 146.7 (C-4'), 112.3 (C-5'), 118.0 (C-6'), 55.9 (C-OCH_3), 100.9 (C-1''), 75.7 (C-2''), 76.5 (C-3''), 70.5 (C-4''), 78.7 (C-5''), 66.3 (C-6''), 99.6 (C-1'''), 72.1 (C-2'''), 73.2 (C-3'''), 71.0 (C-4'''), 69.8 (C-5'''), 18.1 (C-6''').

Neohesperidin, (19): yellow powder, ^{1}H-NMR (CD_3OD, 500 MHz) δ: 6.91 (3H, m, H-2', 5', 6'), 6.15 (2H, m, H-6, 8), 5.34 (1H, m, H-2), 5.23 (1H, d, J = 5.2 Hz, H-1''), 5.06 (1H, d, J = 2.8 Hz, H-1'''), 3.86 (3H, s, H-OCH_3), 1.28 (3H, d, J = 6.2 Hz, H-CH_3); ^{13}C-NMR (CD_3OD, 125 MHz) δ: 77.8 (C-2), 43.7 (C-3), 197.9 (C-4), 162.8 (C-5), 97.7 (C-6), 164.7 (C-7), 96.6 (C-8), 162.3 (C-9), 104.7 (C-10), 132.4 (C-1'), 114.3 (C-2'), 147.5 (C-3'), 149.1 (C-4'), 112.3 (C-5'), 118.8 (C-6'), 56.3 (C-OCH_3), 100.9 (C-1''), 75.7 (C-2''), 76.5 (C-3''), 70.5 (C-4''), 78.7 (C-5''), 66.3 (C-6''), 99.6 (C-1'''), 72.1 (C-2'''), 73.2 (C-3'''), 71.0 (C-4'''), 69.8 (C-5'''), 18.1 (C-6''').

Eriodictioside, (20): yellow powder, ^{1}H-NMR (CD_3OD, 400 MHz) δ_H: 6.99, 6.97 (1H, s, H-2'), 6.82 (2H, br s, H-5', 6'), 6.22 (1H, br s, H-8), 6.20 (1H, br s, H-6), 5.34 (1H, m, H-2), 4.98, 4.97 (1H, d, J = 7.0 Hz, H-1''), 4.73 (1H, s, H-1 '''), 4.10~3.30 (m, 10H, H of sugar moiety), 3.13 (1H, dd, J = 14.1, 12.7 Hz, H-3a), 3.11 (1H, dd, J = 14.0, 12.7 Hz, H-3a), 2.77 (1H, dd, J = 14.0, 2.8 Hz, H-3b), 2.75 (1H, dd, J = 14.0, 2.7 Hz, H-3b), 1.23 (3H, d, J = 6.2 Hz, H-6'''); ^{13}C-NMR (CD_3OD, 100 MHz) δ_C: 80.6, 80.5 (C-2), 44.2, 44.0 (C-3), 198.5, 198.4 (C-4), 164.9, 164.8 (C-5), 98.0, 97.9 (C-6), 166.8, 166.7 (C-7), 97.1, 97.0 (C-8), 164.5, 164.3 (C-9), 105.0 (C-10), 131.6, 131.5 (C-1'), 114.9, 114.7 (C-2'), 146.4 (C-3'), 146.9, 146.8 (C-4'), 116.3 (C-5'), 119.3 (C-6'), 101.1, 101.0 (C-1''), 74.6 (C-2''), 77.8 (C-3''), 72.4, 72.3 (C-4''), 77.1 (C-5''), 67.5, 67.4 (C-6''), 102.1, 102.0 (C-1'''), 71.4, 71.3 (C-2'''), 72.4, 72.3 (C-3'''), 74.1 (C-4'''), 69.7 (C-5'''), 17.9 (C-6''').

Phlorizin, (21): white powder, ^{1}H-NMR (CD_3OD, 400 MHz) δ_H: 7.08 (2H, d, J = 8.2 Hz, H-2, 6), 6.71 (2H, d, J = 8.1 Hz, H-3, 5), 6.16 (1H, d, J = 1.8 Hz, H-3'), 5.96 (1H, d, J = 1.8 Hz, H-5'), 5.84 (2H, br s, H-6, 8), 5.07 (1H, d, J = 6.9 Hz, H-1''), 3.93~3.31 (6H, m, H-2''~6''), 3.48 (1H, d, J =7.8 Hz, H-α), 2.89 (2H, t, J = 7.8 Hz, H-β); ^{13}C-NMR (CD_3OD, 100 MHz) δ_C: 133.9 (C-1), 130.3 (C-2, 6), 116.1 (d, C-3, 5), 156.2 (C-4), 106.4 (C-1'), 162.2 (C-2'), 96.2 (C-3'), 167.1 (C-4'), 99.4 (C-5'), 165.5 (C-6'), 46.7 (C-α), 31.0 (C-β), 206.2 (C=O), 101.9 (C-1''), 74.7 (C-2''), 78.3 (C-3''), 71.1 (C-4''), 78.5 (C-5''), 62.4 (C-6'').

8-Hydroxypinoresinol-4'-*O*-*β*-*D*-Glucopyranoside, (22): white powder, ^{1}H-NMR (CD_3OD, 400 MHz) δ_H: 7.11 (1H, d, J = 8.2 Hz, H-5'), 6.97 (1H, br s, H-2), 6.91 (1H, d, J = 2.0 Hz, H-2'), 6.79 (1H, dd, J = 8.0 Hz, 2.0 Hz), 6.75 (1H, br s, H-5), 6.77 (1H, br s, H-6), 4.85 (1H, d, J = 8.0 Hz, H-1''), 4.84

(1H, s, H-7), 4.07 (1H, dd, J = 8.5, 6.5 Hz, H-9'a), 3.66 (1H, dd, 8.5, J = 3.2 Hz, H-9'b), 3.81 (1H, d, J = 12.0 Hz, H-9a), 3.62 (1H, J = 12.0 Hz, H-9b), 3.87 (3H, s, 3-OCH_3), 3.87 (3H, s, 3'-OCH_3), 3.14 (1H, dd, J = 12.4, 3.4 Hz, H-7'), 2.56 (1H, dd, J = 12.4, 12.0 Hz, H-7'b), 2.62 (1H, m, H-8'); ^{13}C-NMR (CD_3OD, 100 MHz) δ_C: 130.7 (C-1), 112.7 (C-2), 148.6 (C-3), 147.2 (C-4), 115.6 (C-5), 121.5 (C-6), 85.6 (C-7), 83.2 (C-8), 64.5 (C-9), 136.9 (C-1'), 114.3 (C-2'), 150.9 (C-3'), 146.4 (C-4'), 118.2 (C-5'), 122.3 (C-6'), 35.1 (C-7'), 51.7 (C-8'), 71.9 (C-9'), 56.7 (C_3-OCH_3), 56.3 (3'-OCH_3), 103.0 (C-1''), 74.9 (C-2''), 77.8 (C-3''), 71.4 (C-4''), 78.2 (C-5''), 65.5 (C-6'').

Pumilaside A (**23**): colorless powder, ESI-MS *m/z* 441 [M + Na]$^+$, $C_{21}H_{38}O_8$, ^{1}H-NMR (CD_3OD, 400 MHz) δ_H: 4.71 (1H, dd, J = 11.7, 4.5 Hz, H-6), 4.56 (1H, d, J = 7.8 Hz, H-1'), 3.95 (1H, dd, J = 11.7, 2.1 Hz, H-6'a), 3.69 (1H, dd, J = 11.7, 5.9 Hz, H-6'b), 1.98 (1H, d, J = 11.6 Hz, H-5), 1.43 (3H, s, H-15), 1.15 (3H, d, J = 6.8 Hz, H-13), 1.03(3H, d, J = 6.7 Hz, H-12), 1.01 (3H, s, H-14); ^{13}C-NMR (CD_3OD, 100 MHz) δ_C: 80.4 (C-1), 28.8 (C-2), 40.8 (C-3), 74.2 (C-4), 51.4 (C-5), 79.5 (C-6), 42.6 (C-7), 23.7 (C-8), 37.0 (C-9), 42.9 (C-10), 26.5 (C-11), 23.1 (C-12), 23.9 (C-13), 14.5 (C-14), 24.2 (C-15), 100.2 (C-1'), 75.9 (C-2'), 78.5 (C-3'), 72.1 (C-4'), 78.3 (C-5'), 63.2 (C-6').

4-hydroxy-2-methoxyphenol-1-*O*-*β*-*D*-glucopyranoside, (24): white powder, ^{1}H-NMR (CD_3OD, 500 MHz) δ_H: 7.02 (1H, d, J = 8.7 Hz, H-6), 6.47 (1H, d, J = 2.7 Hz, H-3), 6.30 (1H, dd, J = 8.7, 2.7 Hz, H-5), 4.70 (1H, d, J = 7.7 Hz, H-1'), 3.82 (3H, s, 2-OCH_3); ^{13}C-NMR (CD_3OD, 125 MHz) δ_C: 141.1 (C-1), 152.1 (C-2), 104.3 (C-3), 155.0 (C-4), 107.6 (C-5), 120.5 (C-6), 101.8 (C-1'), 75.1 (C-2'), 77.8 (C-3'), 71.4 (C-4'), 78.1 (C-5'), 62.6 (C-6'), 56.8 (C_2-OCH_3).

Phenylethyl-rutinoside, (25): white powder, ^{1}H-NMR (CD_3OD, 500 MHz) δ_H: 7.25~7.28 (4H, m, H-2, 3, 5, 6), 7.18 (1H, m, H-4), 4.76 (1H, d, J = 1.7 Hz, H-1''), 4.30 (1H, d, J = 7.8 Hz, H-1'), 4.04 (1H, ddd, 9.8, 8.0, 6.8 Hz, H-8α), 3.77 (1H, dd, J = 11.2, 1.9 Hz, H-8β), 3.98 (1H, dd, J = 11.2, 1.9 Hz, H-6'α), 3.62 (1H, dd, J = 11.2, 1.9 Hz, H-6'β), 2.95 (2H, ddd, J = 8.1, 6.7, 3.1 Hz, H-7), 1.25 (3H, d, J = 6.2 Hz, H-6''); ^{13}C-NMR (CD_3OD, 125 MHz) δ_C: 140.0 (C-1), 129.4 (C-2, 6), 130.0 (C-3, 5), 127.2 (C-4), 37.3 (C-7), 71.9 (C-8), 104.5 (C-1'), 75.1 (C-2'), 78.1 (C-3'), 71.6 (C-4'), 77.1 (C-5'), 68.1 (C-6'), 102.2 (C-1''), 72.2 (C-2''), 72.4 (C-3''), 74.0 (C-4''), 69.8 (C-5''), 18.0 (C-6'').

Trans-*p*-menthane-1*α*,2*β*,8-triol, (26):white oil, ^{1}H-NMR（CD_3OD，400 MHz） δ: 3.55 (1H, br s, H-2α)，1.20 (s, 3H, CH_3-10), 1.18 (s, 3H, CH_3-9), 1.16 (s, 3H, CH_3-7); ^{13}C-NMR (CD_3OD, 100 MHz) δ: 71.6 (C-1), 74.6 (C-2), 31.1 (C-3), 42.2 (C-4), 22.9 (C-5), 34.4 (C-6), 27.8 (C-7), 73.2 (C-8), 27.1 (C-9), 26.8 (C-10).

Table 1. KEGG enrichment results

ID	Term	Count	P-Value
hsa05200	Pathways in cancer	36	5.36245E-21
hsa05205	Proteoglycans in cancer	20	4.82522E-14
hsa04668	TNF signaling pathway	16	3.44101E-13
hsa05206	MicroRNAs in cancer	22	1.17013E-12
hsa04151	PI3K-Akt signaling pathway	23	2.4144E-12
hsa05222	Small cell lung cancer	14	3.79883E-12
hsa04370	VEGF signaling pathway	12	8.90809E-12
hsa01521	EGFR tyrosine kinase inhibitor resistance	13	1.1499E-11
hsa05417	Lipid and atherosclerosis	18	1.88765E-11
hsa04066	HIF-1 signaling pathway	14	3.02282E-11
hsa04914	Progesterone-mediated oocyte maturation	14	3.8229E-11
hsa05203	Viral carcinogenesis	17	9.02266E-11
hsa05215	Prostate cancer	13	1.34732E-10
hsa04926	Relaxin signaling pathway	14	2.87331E-10
hsa05230	Central carbon metabolism in cancer	11	1.26657E-09
hsa05207	Chemical carcinogenesis - receptor activation	16	1.79264E-09
hsa01522	Endocrine resistance	12	2.41312E-09
hsa04218	Cellular senescence	14	3.02201E-09
hsa04110	Cell cycle	14	3.26743E-09
hsa05167	Kaposi sarcoma-associated herpesvirus infection	15	4.87784E-09
hsa05200	Pathways in cancer	36	5.36245E-21

Table 2. The Vina Scores for molecular docking

Protein	Calp	Hesp
PI3K (6hog)	-8.1	-9.1
AKT (2x18)	-8.6	-8.8
